# Fine Mapping of Dominant *X*-Linked Incompatibility Alleles in *Drosophila* Hybrids

**DOI:** 10.1371/journal.pgen.1004270

**Published:** 2014-04-17

**Authors:** Daniel R. Matute, Jackie Gavin-Smyth

**Affiliations:** 1 Department of Human Genetics, The University of Chicago, Chicago, Illinois, United States of America; 2 The Chicago Fellows Program, The University of Chicago, Chicago, Illinois, United States of America; 3 Ecology and Evolution, The University of Chicago, Chicago, Illinois, United States of America; University of California Davis, United States of America

## Abstract

Sex chromosomes have a large effect on reproductive isolation and play an important role in hybrid inviability. In *Drosophila* hybrids, *X*-linked genes have pronounced deleterious effects on fitness in male hybrids, which have only one *X* chromosome. Several studies have succeeded at locating and identifying recessive *X*-linked alleles involved in hybrid inviability. Nonetheless, the density of dominant *X*-linked alleles involved in interspecific hybrid viability remains largely unknown. In this report, we study the effects of a panel of small fragments of the *D. melanogaster X*-chromosome carried on the *D. melanogaster Y*-chromosome in three kinds of hybrid males: *D. melanogaster/D. santomea, D. melanogaster/D. simulans* and *D. melanogaster/D. mauritiana*. *D. santomea* and *D. melanogaster* diverged over 10 million years ago, while *D. simulans* (and *D. mauritiana*) diverged from *D. melanogaster* over 3 million years ago. We find that the *X*-chromosome from *D. melanogaster* carries dominant alleles that are lethal in *mel/san*, *mel/sim*, and *mel/mau* hybrids, and more of these alleles are revealed in the most divergent cross. We then compare these effects on hybrid viability with two *D. melanogaster* intraspecific crosses. Unlike the interspecific crosses, we found no *X*-linked alleles that cause lethality in intraspecific crosses. Our results reveal the existence of dominant alleles on the *X*-chromosome of *D. melanogaster* which cause lethality in three different interspecific hybrids. These alleles only cause inviability in hybrid males, yet have little effect in hybrid females. This suggests that *X*-linked elements that cause hybrid inviability in males might not do so in hybrid females due to differing sex chromosome interactions.

## Author Summary

The inviability or sterility of interspecific hybrids is one of the mechanisms of reproductive isolation that keep species apart. In this report, we use the genetic tools of *Drosophila melanogaster* to assess the cytological locations and relative frequency of dominant *X*-linked alleles involved in hybrid inviability in three different interspecific crosses. We map the genomic regions of the *D. melanogaster X*-chromosome that cause inviability in hybrids produced by *D. melanogaster* females crossed to males of three other *Drosophila* species: *D. simulans*, *D. mauritiana* and *D. santomea*. For each hybrid inviability allele we identified, we characterized the developmental defects that occur in the inviable hybrids. Our results show that the effect of these *X*-linked lethal regions is lineage-specific, as is the total number of alleles that cause hybrid inviability. These results can be expanded and will allow for the exact identification of *X*-linked *D. melanogaster* alleles in these three different hybrid contexts.

## Introduction

One of the most intensely studied forms of reproductive isolation is intrinsic postzygotic isolation: the inviability or sterility of interspecific hybrids that arises during development. The genetic mechanisms underlying this type of reproductive isolation are thought to be irreversible in evolutionary time [1], [2]. The study of postzygotic isolating mechanisms can reveal the molecular changes that have arisen between species [3], [4]. There is both theoretical and empirical evidence for the role of postzygotic isolation in completing the process of speciation through the action of natural selection [5]–[7], as enhanced prezygotic isolation might evolve as a byproduct of maladaptive hybridization, thus furthering the speciation process [6], [8].

In the Dobzhansky-Muller model (DM model) of the evolution of reproductive isolation, the genetic basis of hybrid breakdown involves (at minimum) two loci with an ancestral genotype of *A_1_A_1_B_1_B_1_*. The ancestral species splits into two descendant species that eventually acquire genotypes *A_1_A_1_B_2_B_2_* and *A_2_A_2_B_1_B_1_* through natural selection, meiotic drive or, less likely, genetic drift. This model posits that postzygotic isolation arises in allopatry as a collateral effect of the evolutionary divergence between these two isolated populations. In this case, although species having genotypes *A_1_A_1_B_2_B_2_* and *A_2_A_2_B_1_B_1_* at two loci are fit, the hybrid progeny will have a genotype *A_1_A_2_B_1_B_2_* and therefore might be unfit: either sterile or inviable because the *A_2_* and *B_2_* alleles do not interact properly [1], [3], [9], [10]. The DM model presents a general mechanism for the evolution of postzygotic isolation, and explains a substantial proportion of the cases in which we know the genetic basis of hybrid breakdown [for exceptions see 11], [12], [ reviewed in 13].

Concerted mapping efforts have localized a number of hybrid incompatibility genes (those involved in Dobzhansky-Muller incompatibilities, or DMI) to small chromosomal regions [reviewed in 4], [13], [14] and have yielded some general patterns about the biology of genes involved in reproductive isolation. The first general pattern of hybrid inviability, Haldane's rule, pre-dates genetic mapping. In a wide variety of organisms, if hybrids of only one of the sexes are inviable or sterile, it will be the heterogametic sex [1], [15], [16]. Second, many (but not all) of the genes that cause hybrid breakdown have evolved under the influence of natural selection or meiotic drive, suggesting that rapid evolution within species leads to the evolution of DMI in hybrids [17]–[19]. Third, the *X*-chromosome, when compared with the autosomes, plays a disproportionately large role in postzygotic isolation [1], [20]. Fourth, mapping results have shown that the predictions from the DM model hold at the genomic level, and that the number of genes involved in hybrid inviability evolves faster than the accumulation of neutral genetic differences between species [21]–[23]. Finally, hybrid incompatibilities are asymmetric (i.e, *A_2_* is incompatible with *B_2_*, but *A_1_* may be compatible with *B_1_*). These asymmetries often result from DMIs involving uniparentally inherited genetic factors such as cytoplasmic–nuclear interactions [24]–[29].

Of these five patterns, two—Haldane's rule and the large effect of the *X*-chromosome on hybrid inviability—can be explained by the hemizygosity of the sex chromosome and the dominance theory ([30]–[33]; see [34] for alternative explanations of Haldane's rule in animals lacking a heterogametic sex). In *Drosophila*, *X*-linked genes can have a disproportional effect on hybrid fitness because the heterogametic hybrid males suffer from both the dominant and recessive deleterious *X*-linked alleles [35]–[37]. In the homogametic females, however, the deleterious effects of recessive alleles are masked by the presence of a second *X*-chromosome. In *Drosophila* hybrids, several studies have suggested the presence of recessive alleles in one of the *X*-chromosomes that can cause hybrid inviability in females when uncovered with a genetic lesion [38]. Surprisingly, in some of these crosses, hybrid males are viable despite all the recessive factors from one *X*-chromosome being fully exposed [31], [39]. One hypothesis for why males, but not females, can survive in these cases is that epistatic interactions *between* the two *X*-chromosomes lead to inviability in females. This idea, first formalized by Orr [30], states that since female hybrids carry two *X*-chromosomes, they suffer twice as many interactions involving the sex chromosomes but as the alleles involved in hybrid breakdown are on average recessive, the heterogametic sex is still much more prone to suffer hybrid breakdown. The hypothesis that the homogametic sex (females in *Drosophila*) suffers from negative epistatic interactions between *X*-chromosomes remains largely untested (but see [40], [41]). One of the prerequisites of such interactions is the existence of dominant partners on one of the *X*-chromosomes that could potentially cause hybrid inviability in females but not in males. The aim of this study is to determine if this sex-specific epistasis is present in interspecific hybrids and reveal dominant partners in the interactions.

*Drosophila melanogaster* is particularly useful for the study of the genetic architecture of hybrid inviability because of its armamentarium of genetic tools that can be used to establish the identity and density of alleles involved in DMIs. To date, two studies have used deficiencies in *D. melanogaster*, cytological aberrations in which a chromosomal segment is deleted, to reveal recessive alleles from the paternal species that cause inviability in *D. melanogaster/D. simulans* hybrids due to interactions with dominant partners in the *D. melanogaster* genome. Coyne et al. [38] found three lethal regions in the *D. simulans* genome with only one of those alleles located on the *X*-chromosome. Matute et al. [22] identified a total of 11 *D. simulans* recessive alleles, including two on the *D. simulans X*-chromosome (*X^sim^*) that interacted with the *D. melanogaster* genome. In parallel, Matute et al. [22] also aimed to dissect causes of inviability in hybrids between the more diverged species *D. santomea* and *D. melanogaster* and determined that at least 71 genomic regions were involved in hybrid inviability. The results from that study indicated at least 13 recessive alleles residing on the *D. santomea X*-chromosome (*X^san^*) cause hybrid inviability.

However, these deficiency-mapping efforts focused on identifying a single hybrid inviability allele from what certainly could be complex epistatic interactions (involving three or more loci; [3], [19], [42]–[45]). These studies both localized recessive alleles in the genome of the paternal species (either *D. simulans* or *D. santomea*) that are involved in DMIs, but did not explore their possible partners in the maternal genome (*D. melanogaster*). QTL-mapping and introgression-based approaches share the same drawback: even though they reveal a portrait of the genes involved in hybrid breakdown (e.g., [46], [47]), they do not reveal the full nature of the genetic architecture of hybrid inviability as they do not identify the specific epistatic interactions leading to reduced fitness of hybrids. Three studies have aimed not only to identify single alleles that contribute to hybrid inviability but also to determine the interacting partners of such alleles. Presgraves [48] pursued a genome-wide identification of *D. simulans* autosomal recessive alleles that cause lethality in male *D. melanogaster/D. simulans* hybrids (*mel/sim*). In this case, if the *D. simulans X*-chromosome was present (as in hybrid females), the autosomal recessive alleles did not cause hybrid inviability, suggesting the existence of epistatic recessive partners on the *D. melanogaster X*-chromosome (*X^mel^*). This initial screening allowed for the identification of two autosomal nuclear pore proteins (*Nup96-98* [49] and *Nup160* [50] that in conjunction with unidentified recessive alleles in *X^mel^* cause inviability in *mel/sim* hybrids.

Sawamura and Yamamoto [40] used a *D. melanogaster* translocation from the *X*-chromosome attached to the *Y*-chromosome [*Dp(1;Y)* translocation; Precise genotype: *Ts(1Lt;Ylt)Zhr/Dp(1;Y)y^+^;*.40] to identify a dominant *X*-linked allele that causes lethality in hybrid *sim/mel* sons, and named it *zhr* (zygotic hybrid rescue). Fine functional analyses, also aided by the use of *Dp(1;Y)* translocations have revealed that *zhr* is a repetitive 359 bp DNA satellite, derived in *D. melanogaster* and absent in *D. simulans*, that causes hybrid inviability by causing heterochromatin packing problems which in turn leads to mitotic defects early in embryogenesis [51].

Finally, Cattani and Presgraves [41] expanded the results from Coyne et al. [38], and identified a candidate dominant factor on *X^mel^* that could cause hybrid inviability when interacting with one of the *D. mauritiana X*-linked recessive alleles. Their results point to the existence of one dominant factor that interacts with at least one recessive factor in the heterochromatic region of the *D. mauritiana X*-chromosome to cause hybrid lethality. These two mapping efforts have uncovered most of the known *X*-linked dominant DMI partners in *Drosophila*.

Here, we explore the possibility of negative epistatic interactions between *X*-chromosomes in several interspecific hybrids by taking advantage of a comprehensive tiling set of duplications of the *D. melanogaster X*-chromosome attached to the *Y*-chromosome [52]. In this report, we show the possibility of producing *D. melanogaster/D. santomea* hybrid males. Second, we examine inviable hybrid males from several crosses (*D. melanogaster/D. santomea*, *D. melanogaster/D. simulans* and *D. melanogaster/D. mauritiana*) to study the effect of small regions of the *D. melanogaster X*-chromosome in an across-species comparative manner; this revealed significant differences in the frequency, developmental timing and lineage specificity of epistatic interactions between *X*-chromosomes in *Drosophila* hybrids.

## Results

### Hybrid *mel/san* males are viable if they carry a *D. santomea X*-chromosome

**Figure 1:**
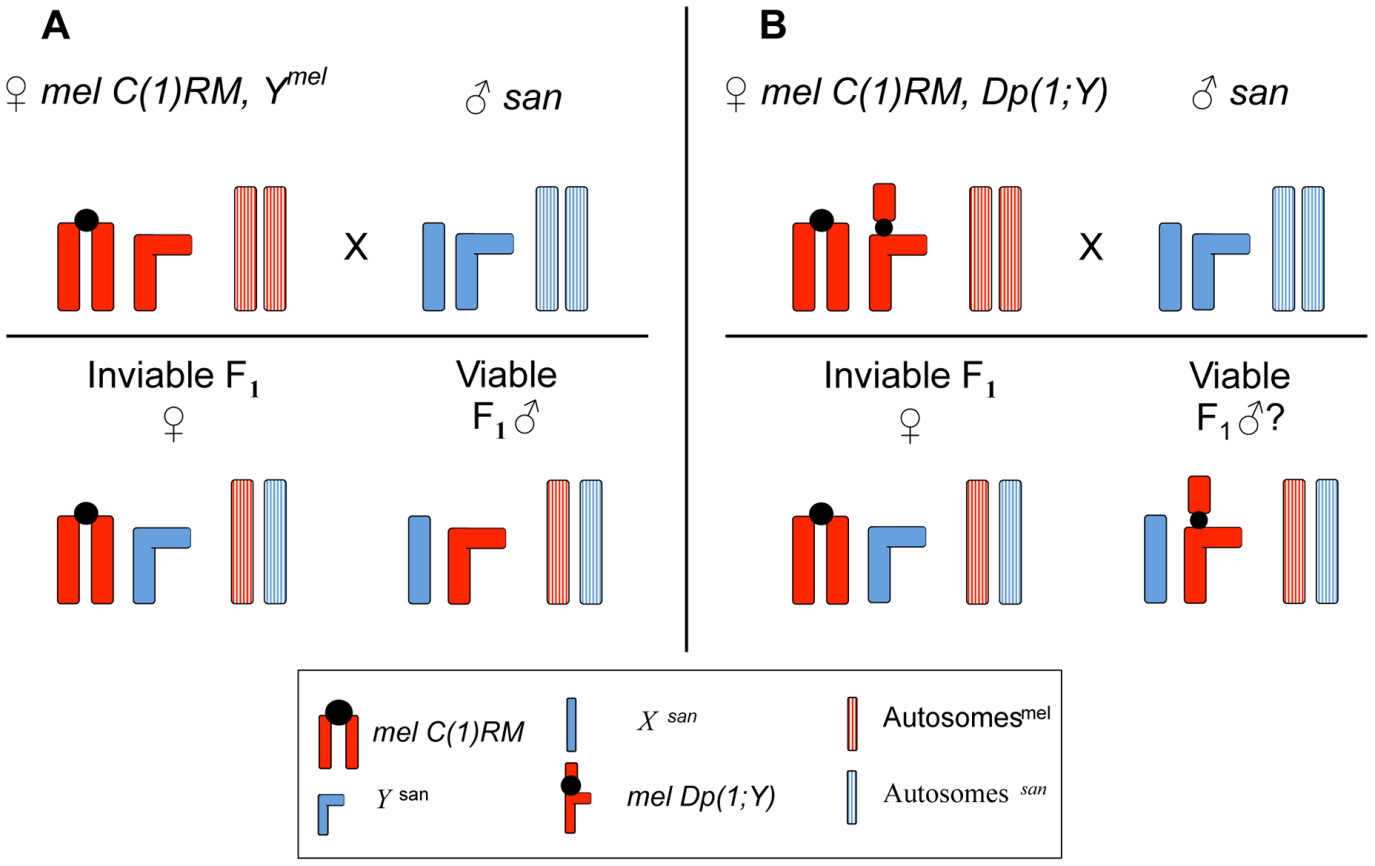
Crossing scheme to study the effect of *X*-linked chromosomal duplications in *mel/san* hybrid males. A. Crosses between *D. melanogaster* females carrying an attached-*X* [*C(1)RM*] chromosome and *D. santomea* males produce dead females and viable adult hybrid males. (The *Y^mel^*-chromosome has no effect on hybrid male viability, see text.) B. *D. melanogaster* females carrying an attached-*X* [*C(1)RM*] chromosome and a compound *Y*-chromosome (i.e, an *X*-chromosome duplication attached to the *Y^mel^*-chromosome) are viable and fertile. These females can be crossed to *D. santomea* males, and even though all the females die as embryos, males survive if the *Dp(1;Y^mel^)* does not carry dominant (or semi-dominant) lethal alleles. We were able to tile 72% of the whole euchromatic *X*-chromosome from *D. melanogaster*. Results for this screening are shown in Figure 3.

The cross of wild-type *D. melanogaster* females to wild-type *D. santomea* males produces only sterile adult female progeny while males with the genotype *X^mel^/Y^san^* die as embryos [47]. The reciprocal cross does not produce any progeny as premating isolation seems to be complete in that direction [53]. Recently, we discovered that when *mel* attached-*X* females with a Compound Reversed Metacentric chromosome, *C(1)RM/0*, are crossed to *D. santomea* males, they produce progeny entirely composed of adult hybrid males with the genotype *X^san^/0* while the hybrid females die as embryos (Figure 1). We also observed that the cross of a second type of attached-*X* females, *mel C(1)RM/Y^mel^*, to *D. santomea* males produces solely *X^san^/Y^mel^* hybrid males. Crosses involving an alternative *mel* attached-*X*, Compound (1) Double X or *C(1)DX*, produce identical results. Figure S1 shows two morphological traits of these previously undescribed hybrid males, number of teeth in the sex combs and abdominal pigmentation.

Because we can produce hybrid males with and without *Y^mel^*, we asked first whether this chromosome had any affect on hybrid male viability, or longevity. We first compared *X^san^/Y^mel^* to *X^san^/0* hybrid males. (These males were generated by *mel* attached-*X* females carrying a *Y^mel^* (either *C(1)RM/Y^mel^* or *C(1)DX/Y^mel^*)×*D. santomea* males and attached-*X mel* females carrying only the homocompound chromosome *(C(1)RM/0* or *C(1)DX/0)*×*D. santomea* males respectively.) Males of these two genotypes showed no differences in viability at any developmental stage (Figure S2). The two types of hybrid males were both sterile and showed similar longevity (Figure S2; Tables S1 and S2). Despite the morphological defects of these hybrid males, particularly in abdominal segments, they survive almost as long as virgin males from both parental species (Figure S2). The fitness of the *X^san^/0* and *X^san^/Y^mel^* hybrids is effectively zero as both are sterile, however, these results demonstrate that there are no lethal epistatic interactions between *X^san^* and *Y^mel^*, between the *D. santomea* autosomes and *Y^mel^*, or between *X^san^* and the cytoplasmic elements, including mitochondrial genes or maternally deposited gene products, of *D. melanogaster*.

We then excluded the possibility that the *mel/san* males could actually be feminized or sexually chimeric. We assessed the presence of male-specific structures on both sides of the body in *X^san^/0* males produced from the cross *mel C(1)RM/0*×*san*. The *mel/san* hybrid males are bilaterally symmetrical, with sex combs, testes and genital arches on both sides. More specifically, the number of teeth in their sex-combs is not significantly different between left and right (Paired Wilcoxon signed rank test with continuity correction on sex comb teeth, left vs. right: V = 4, P = 0.850) and did not have any feminized features (Figure S1). All hybrid males were sterile and had atrophied testes lacking motile sperm. These results indicate that these hybrids are true males are not gynandromorphs or otherwise sexually chimeric.

Following [31], we were also able to perform interspecific crosses between *mel C(1)RM/0* females and *D. simulans* (*sim*), or *D. mauritiana* (*mau*) males. These crosses produce viable hybrid *X^sim^/0* or *X^mau^/0* males. We were also able to produce *X^sim^*/*Y^mel^* and *X^mau^/Y^mel^* hybrid males by crossing *mel C(1)RM/Y^mel^* to *sim* or *mau* males, respectively. *X^sim^*/*Y^mel^* and *X^sim^/0*, as well as *X^mau^*/*Y^mel^* and *X^mau^/0* males show similar viability and longevity to the pure parental species regardless of the genetic background of the attached-*X D. melanogaster* stock (Tables S1 and S2). The existence of these viable hybrid males, similar to the existence of *mel/san* hybrids, indicates there no lethal epistatic interactions exist between *X^sim^* (or *X^mau^*) and *Y^mel^*, between *Y^mel^* and the *D. simulans* (or *D. mauritiana*) autosomes, or between *X^sim^* (or *X^mau^*) and the cytoplasm of *D. melanogaster* (Figure S3 and S4).

**Figure 2:**
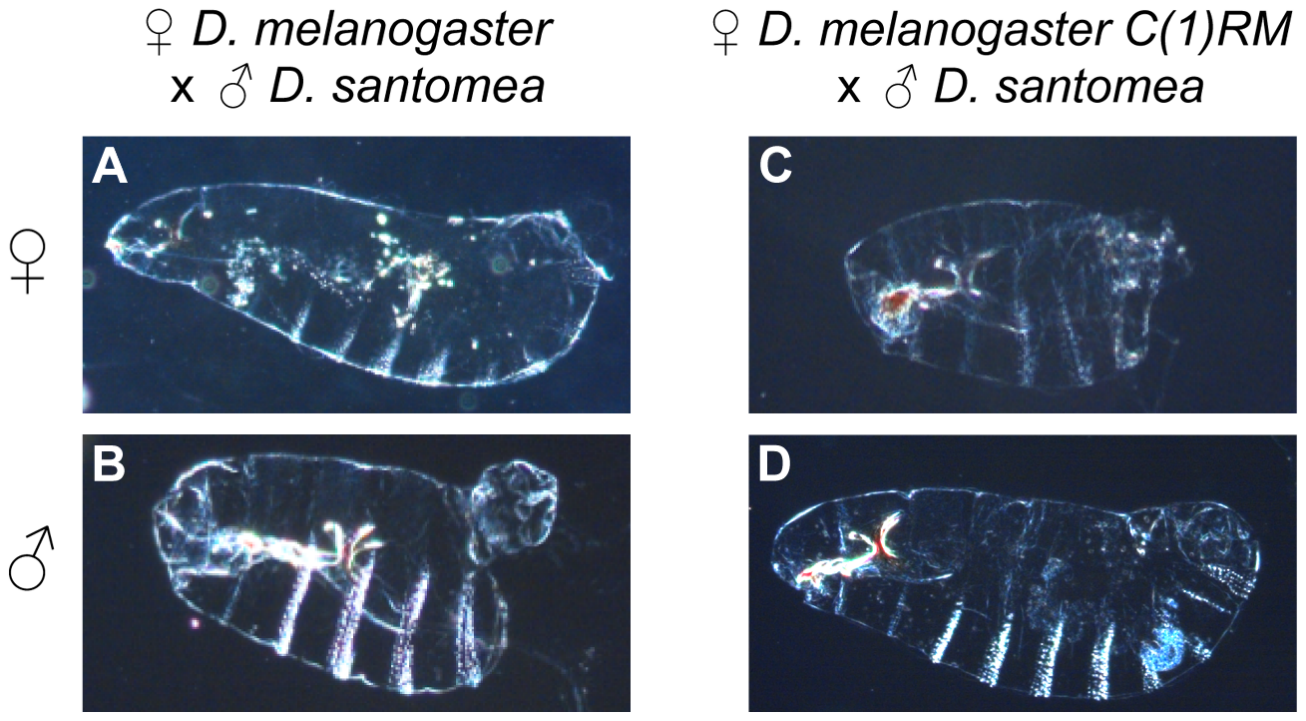
Developmental defects observed in two interspecific crosses between *D. melanogaster* and *D. santomea*. In crosses of wild-type *D. melanogaster* females and *D. santomea* males, the majority of females (*X^mel^/X^san^*) emerge as adults. Those that fail to hatch from embryogenesis manifest cuticular defects typified by (A). Hybrid male embryos (*X^mel^/Y^san^*) from this cross usually die and show severe abdominal ablations (B). In crosses between *D. melanogaster C(1)RM* ♀ and *D. santomea* ♂, the majority of hybrid females embryos (*X^mel^X^mel^/Y^san^*) fail to hatch and show abdominal ablations (C), while hybrid males (*X^san^/0*) usually survive. Those that fail to hatch are typified by (D).

In all three interspecific crosses involving *mel C(1)RM/Y^mel^* (and *mel C(1)RM/0*) females, hybrid females that carry the two *X^mel^* rarely survive to adulthood. In the case of *mel C(1)RM/0*×*san* crosses, hybrid females (*X^mel^X^mel^/Y^san^*) predominantly die as embryos, the same developmental stage at which wild-type hybrid males (*X^mel^/Y^san^*) die. Hybrid female embryos carrying only the *C(1)RM* chromosome manifest an abdominal ablation in the posterior, very similar to that which we described for wild-type hybrid males who carry an *X^mel^* [53]. This phenotype is present in 71% of the hybrid (*X^mel^X^mel^/Y^san^*) females, comparable to the 67% frequency seen in wild-type *X^mel^*/*Y^san^* males, (Figure 2, [53]). In the case of [*C(1)RM/0*×*sim*], and [*C(1)RM/0*×*mau*] crosses, the hybrid females carrying the compound attached-*X^mel^* chromosome are able to survive through their larval stages but do not transition into pupae, similar to *mel/sim* (and *mel/mau*) wild-type hybrid males [54]–[56]. These results indicate that either one or two copies of *X^mel^* chromosome in the absence of another *X*-chromosome can induce hybrid inviability regardless of the sex of the hybrid in the three interspecific crosses.

To determine which factors residing on *X^mel^* are involved in hybrid inviability we then undertook a duplication mapping screen to identify the regions of *X^mel^* that cause lethality in males carrying a *D. santomea*, *D. simulans* or *D. mauritiana X*-chromosome and a fragment of *X^mel^* attached to the *Y^mel^* [*Dp(1;Y)*]. Our goal was to identify the dominant regions on *X^mel^* that can cause hybrid lethality in the presence of the *X*-chromosome from another species. All fly stocks are listed in Tables S3 and S4.

### The *X*-chromosome from *D. melanogaster* contains dominant alleles that cause hybrid inviability in *mel/san* hybrid males

As the duplication mapping approach [40], [41] has no balancer sibling or other internal controls, this study is limited to the identification of alleles that cause complete (rather than partial) lethality. We used two different criteria to describe dominant lethals. First, we used a qualitative cut-off: an allele was classified as lethal if fewer than 10% of individuals hatched or molted to the next developmental stage. This approach is limited because the cut-off is arbitrary, but our data were resilient to more quantitative analyses (see Methods). Second, the duplication had to cause lethality in both attached-*X* genetic backgrounds; *mel C(1)RM* and *mel C(1)DX*. This approach therefore does not detect putative semi-lethal alleles or those that can cause significant, but incomplete, reductions in viability. To exclude pre-mating isolation from our observations, we only included data from matings in which we observed inseminated females. Twenty females from each of three replicates were dissected for each cross and their reproductive tracts were inspected for the presence of sperm, either motile or dead. Table S5 shows insemination rates for the three interspecific crosses and the two intraspecific crosses, involving *mel C(1)RM, Dp(1;Y)* females. To further exclude postmating-prezygotic isolation between the two species involved in the cross, herein we only include data from matings that produced embryonic progeny.

When *C(1)RM, Dp(1;Y^mel^)* females hybridize with *D. santomea* males, four genotypes are produced: [*Y^san^*/*Dp(1;Y^mel^)*], [*C(1)RM*/*X^san^*], [*C(1)RM*/*Y^san^*], and [*X^san^/Y^mel^*, *Dp(1;Y)*]. Embryos with the genotype [*Y^san^*/*Y^mel^, Dp(1;Y)*], also called nullo-*X* embryos, do not complete cellularization in the early blastoderm stage and fail to differentiate any discernible larval cuticle [57]. Thus, on a gross phenotypic level, they are indistinguishable from unfertilized eggs. Hybrid metafemales embryos, with 3 *X*-chromosomes *C(1)RM*/*X^san^*, also routinely fail to hatch. These females cannot be differentiated from hybrid males as both carry a wild-type *yellow* allele and have black mouthparts. Adult hybrid metafemales are not to be expected as, even in pure species crosses within *D. melanogaster*, the frequency of metafemale survival to eclosion is less than 0.2% of total females [58]. In this study male (*X^san^/Dp(1;Y)*) embryonic lethality was assessed in a qualitative way (i.e, an *X^mel^* region was considered lethal if less than 10% of the total progeny hatched to L_1_), such that the lack of distinction between hybrid males and metafemales was not a substantial concern. Adult hybrid metafemale or attached *X^mel^*/*Y^san^* females were never recovered in any of the crosses (but see [59]–[62] for studies on viability of hybrid *mel/sim* metafemales). The remaining two genotypes from these crosses are [*C(1)RM/Y^san^*] females, of which nearly all die as embryos. A majority of these animals manifest abdominal ablations, and are distinguished by their lack of black pigment in their larval mouth parts. The majority of males of the final genotype, [*X^san^*/*Dp(1;Y^mel^)*] males, hatch into L_1_ and develop into adults, unless the duplication carried on the *Y^mel^* chromosome contains a dominant lethal allele which induces hybrid inviability. When *C(1)RM, Dp(1;Y^mel^)* females are hybridized with *D. simulans* or *D. mauritiana* males, four analogous genotypes are produced with identical survivability.

**Figure 3:**
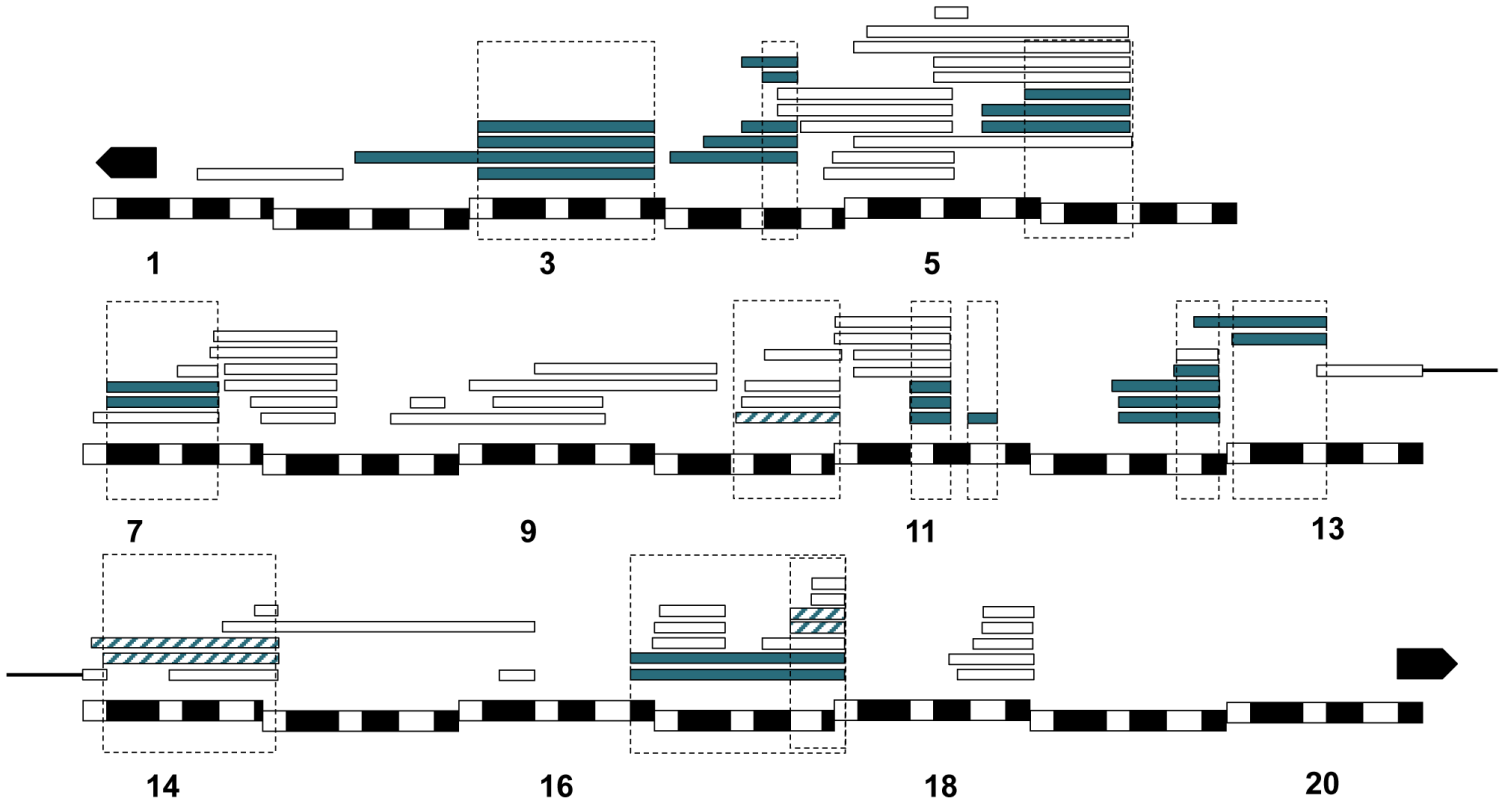
Mapping results from *mel/san* crosses. We crossed *D. melanogaster C(1)RM/Dp(1;Y)* females to *D. santomea* males and assessed what proportion of the males were dead at different stages of development. All the hybridizations that produced progeny are represented by a rectangle. The *X^mel^* regions [*Dp(1;Y)*] that caused hybrid inviability are shown in two shades of blue depending on where they caused hybrid inviability: solid blue (embryonic lethality), and blue stripes (pupal lethality). Chromosomal segments that did not cause lethality are not colored. Please note the region on 17 contains two lethal regions.

In total, thirty-one duplications carrying twelve distinct chromosomal regions in *X^mel^* caused hybrid lethality at some developmental stage in *C(1)RM, Dp(1;Y^mel^)*×*san* crosses (Figure 3, Table S6). Out of these twelve regions, nine caused complete embryonic lethality, none caused male larval lethality and three caused male pupal lethality.

We followed the same approach using a different *D. melanogaster* background and a different attached-*X* chromosome; *C(1)DX*. In addition to the twelve lethal regions identified in the *C(1)RM* background, we detected one additional locus that induces hybrid lethality in the *C(1)DX*/*Dp(1;Y^mel^)*×*san* crosses (Table S7). The lethality of this additional region, [18F4-19A2; 19A2], in the *C(1)DX* genetic background is likely due to an increased sensitivity resulting in higher overall lethality rates. We therefore focused only on the twelve duplications that caused lethality in both backgrounds. Figure 3 shows the cytological position of each of the twelve *X^mel^*-linked lethal alleles.

In wild-type crosses involving *D. melanogaster*×*D. santomea*, we found that the hybrid males, which carry the full *X^mel^*-chromosome (*X^mel^/Y^san^*), die as embryos with profound abdominal ablations [53]. While some hybrid females also manifest abdominal segment pattern defects, they are not as severe, suggesting the presence of the *X^san^* chromosome ameliorates the patterning defects. Hybrid female embryos carrying only *X^mel^*-chromosomes (genotype attached-*X C(1)RM/Y^san^*) have a much higher penetrance of the abdominal ablation phenotype than the *X^mel^X^mel^/X^san^* metafemale embryos (Figure 2). Parallel analysis with an alternative attached-*X* chromosome, *C(1)DX*, shows that 64.6% (SEM = 4.1%) of *C(1)DX*/*Y^san^* female embryos manifest severe abdominal ablations. These results indicate that *X^mel^* carries at least one allele that induces abdominal ablations in both wild-type hybrid males and in *X^mel^X^mel^/Y^san^* hybrid females. This excludes a simple *X*-chromosome dosage effect as the cause of lethality in the wild-type hybrid male. The incomplete inviability of *X^mel^/X^san^* females in crosses between wild type *D. melanogaster* females and *D. san* males indicates that the allele or alleles on *X^mel^* must act semi-dominantly. As 71.5% (SEM 9.3%) of *C(1)DX/X^san^* metafemales have complete abdominal structures, this implies that in both the wild-type hybrid females and hybrid metafemales, *X^san^* can serve to partially rescue the ablation phenotype, but not the embryonic lethality of hybrid metafemales.

**Figure 4:**
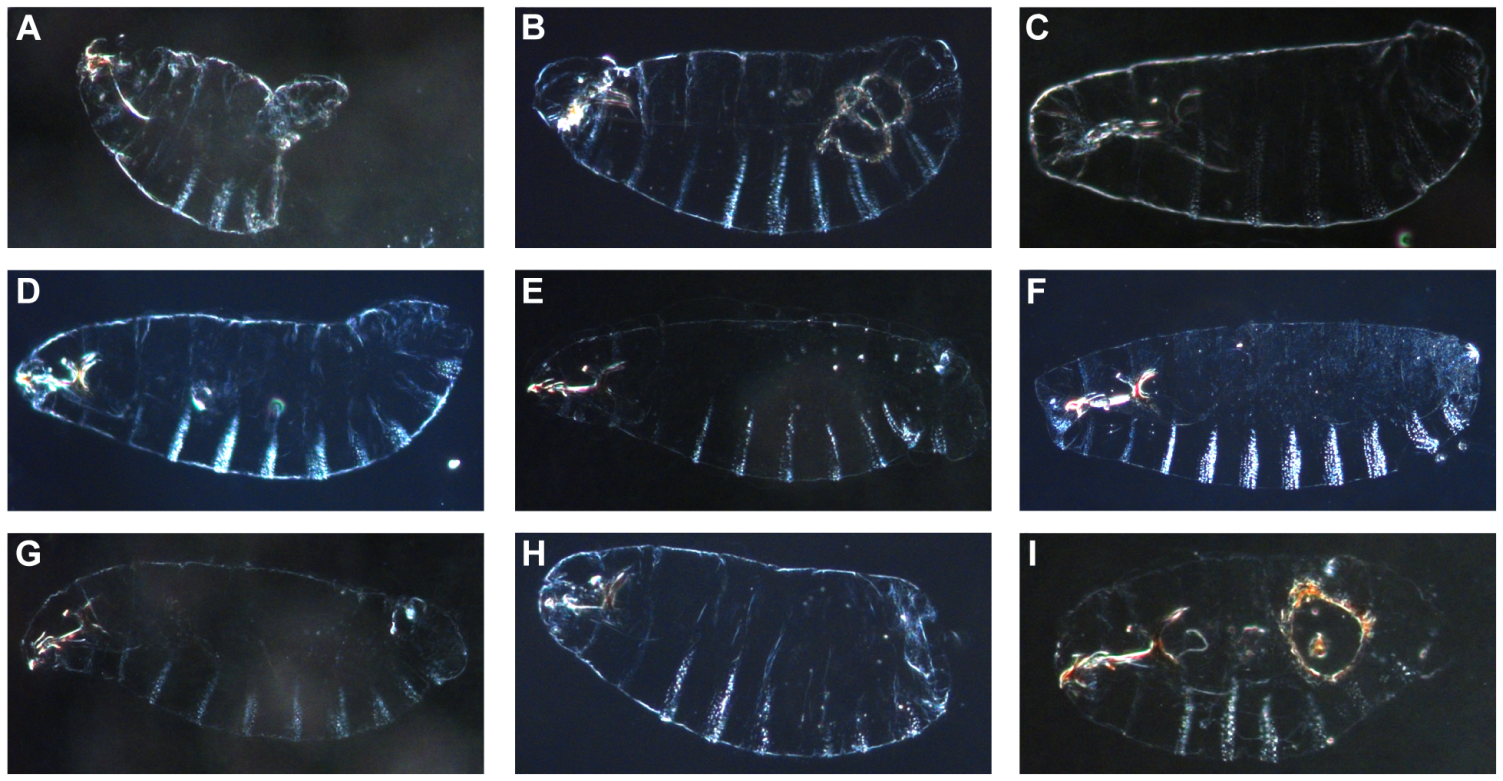
Cuticular defects observed in the nine lethal regions from the *D. melanogaster X*-chromosome. A. Males carrying the 3A–3D region show the abdominal ablation observed in *X^mel^/Y^san^* hybrid males and *X^mel^X^mel^/Y^san^* hybrid females. B–I. Hybrid males that carry any of the other eight embryonic lethal *X^mel^*-linked regions show different phenotypes and the abdominal ablation is more rare than in *X^mel^/Y^san^* males. The genotypes (and the cytological bands of the duplication) of each of the shown males are: A. *Dp(1;Y)BSC75* (*X*:2C1-3E4). B. *Dp(1;Y)BSC159* (*X*:4A5-4D7). C. *Dp(1;Y)BSC289* (*X*:5E1-6C7). D. *Dp(1;Y)BSC176* (*X*:7B2-7D18). E. *Dp(1;Y)BSC126* (*X*:11C2-11D1). F. *Dp(1;Y)BSC327* (*X*:11D5-11E8). G. *Dp(1;Y)BSC186* (*X*:12C1-12F4). H. *Dp(1;Y)BSC269* (*X*:12E9-13C5). I. *Dp(1;Y)BSC11* (*X*:16F6-18A7).

We then searched for the region or regions of *X^mel^* responsible for this abdominal ablation phenotype. We analyzed the cuticular phenotypes of the *Dp(1;Y^mel^)*/*X^san^* male embryos which failed to hatch to discern whether one or more alleles residing on *X^mel^* caused this developmental perturbation. The cuticular phenotypes of these nine genotypes are shown in Figure 4. A typological analysis shows the major morphological differences between these nine genotypes. The allele(s) which induce abdominal ablation and hybrid inviability appears to reside on the tip of the *X^mel^*-chromosome, as that cuticular defect manifests significantly more frequently when this region is present (Figure S4). All the other regions from *X^mel^* that cause embryonic hybrid inviability have a substantially reduced frequency of abdominal ablations (Figure 4, Figure S4) but frequent head involution and segmentation defects.

**Figure 5:**
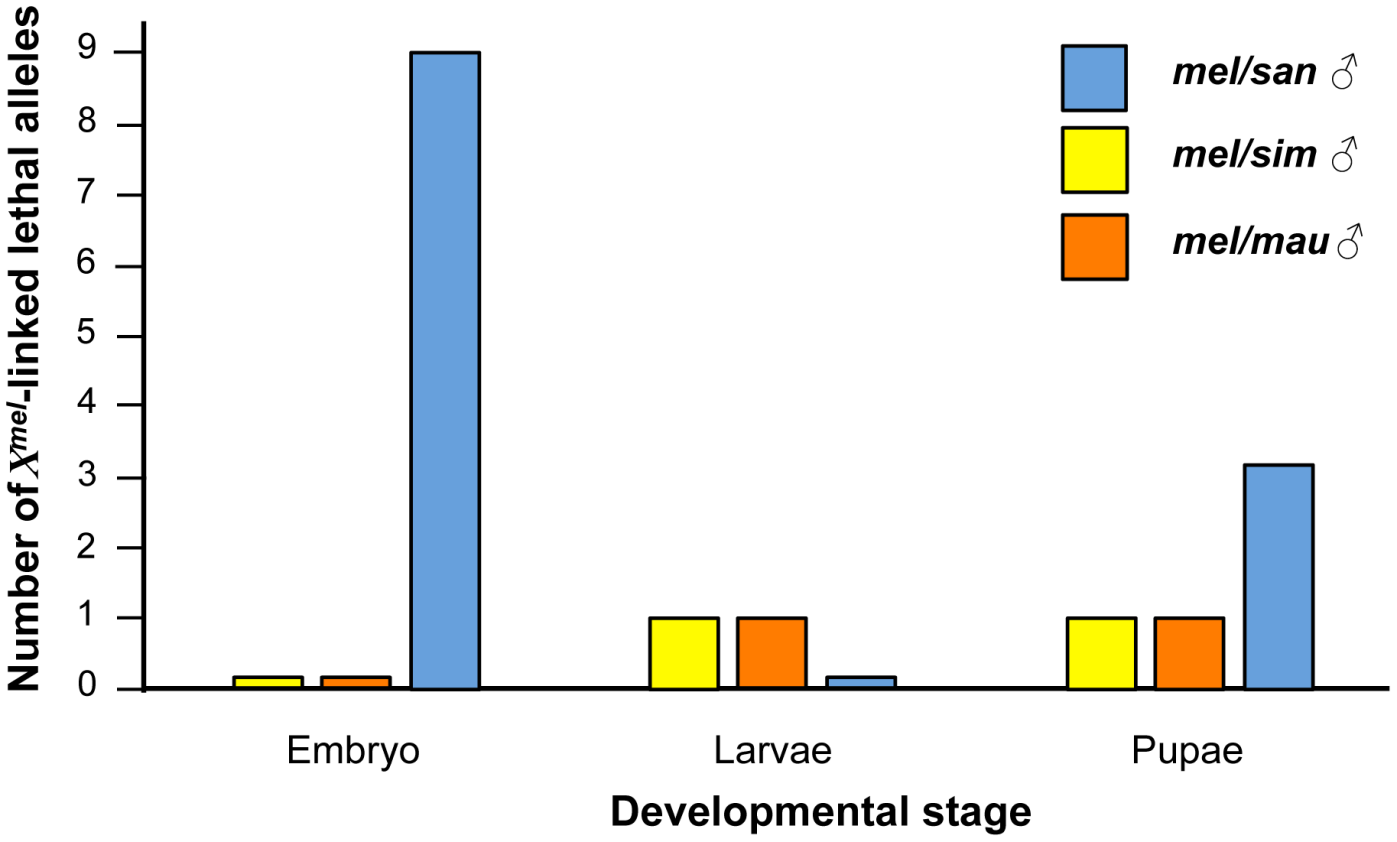
Some developmental stages are more prone to show hybrid inviability than others in *mel/san* hybrids. *X^mel^* alleles that cause hybrid inviability in *mel/san* hybrid males are not uniformly distributed across development, and are more likely to act either at embryonic or pupal stages (Blue: *mel/san* hybrids; Orange: *mel/mau* hybrids; Yellow: *mel/sim* hybrids). The uniformity of the effects was assessed neither in *mel/sim* nor in *mel/mau* hybrids given the scarcity of *X^mel^* lethals in these crosses.

We could assess whether alleles caused hybrid inviability in *Dp(1;Y^mel^)*/*X^san^* hybrid males at a particular developmental stage, or whether their effects were uniformly distributed across embryonic, larval, and pupal stages. We found 9 alleles causing embryonic lethality, with none causing larval lethality, and three causing pupal lethality (Figure 5). The distribution of alleles involved in inviability at different stages of development in *Dp(1;Y^mel^)*/*X^san^* hybrid males significantly departs from the expectation that alleles causing hybrid lethality would be uniformly distributed across all stages of development (Figure 5, comparing the observed frequency of lethal alleles at each developmental stage with a uniform distribution of lethals across development (i.e., 4 lethals at each stage; χ^2^ = 10.5, df = 2, P = 5.25×10^−3^). These results suggest that in *Dp(1;Y^mel^)*/*X^san^* hybrid males, the very early and very late stages are more prone to failures in proper development.

### The *X*-chromosome from *D. melanogaster* contains dominant alleles that cause hybrid inviability in *mel/mau* and *mel/sim* hybrid males

We followed a similar approach to identify *X^mel^*-linked lethal alleles in *mel/mau* and *mel/sim* hybrids. We crossed females from the *C(1)RM*, *Dp(1;Y)* and *C(1)DX, Dp(1;Y)* panels to males of *D. mauritiana* and *D. simulans*. In the same way we assessed the *mel/san* hybrids, we measured hatching rates and male viability at different developmental stages for these two interspecific crosses.

**Figure 6:**
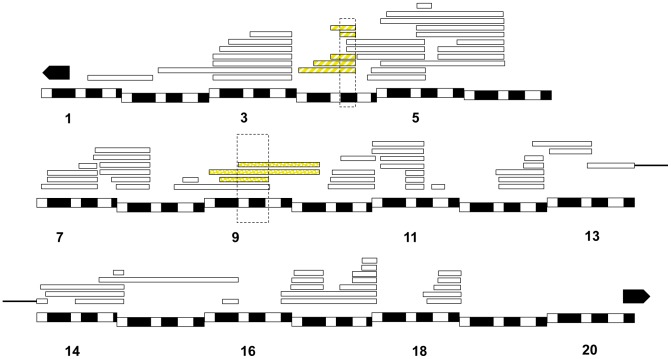
Relative frequency of hybrid lethal alleles in the *D. melanogaster X*-chromosome in hybrid males with *D. simulans*. Two regions from *X^mel^* cause hybrid lethality in *mel/sim* hybrid males (one of them encompassing *Hmr^mel^* and *CG11160^mel^*). The two regions are shared with *mel/mau* hybrid males and also cause hybrid lethality in postembryonic stages. The chromosomal segment that causes larval lethality is dotted, while the region that causes pupal lethality is striped. Chromosomal segments that did not cause lethality are not colored.

**Figure 7:**
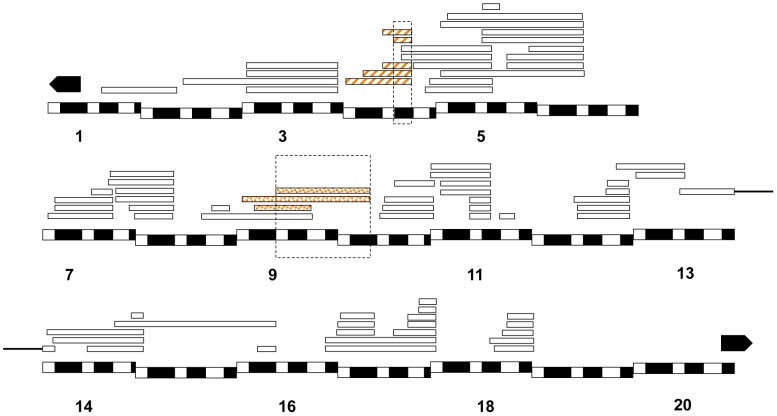
Relative frequency of hybrid lethal alleles in the *D. melanogaster X*-chromosome in hybrid males with *D. mauritiana*. Only two regions from *X^mel^* were lethal in *mel/mau* crosses (one encompassing *CG11160^mel^ and Hmr^mel^*). Both of these lethal regions act during postembryonic development The chromosomal segment that causes larval lethality is dotted, while the region that causes pupal lethality is striped. Chromosomal segments that did not cause lethality are not colored.

In *Dp(1;Y^mel^)/X^sim^* males, we found that two unique regions (eight *X^mel^* fragments) that caused larval lethality in both *mel* attached-X backgrounds (Figure 6). Similarly, in *mel/mau* males, we found two unique regions (eight *X^mel^* fragments) that caused hybrid inviability (Figure 7). One region, between 4C and 4D, causes adult hybrid inviability in all the three assayed hybrids. The region between 9C and 10B causes lethality in both *mel/sim* and in *mel/mau* hybrids. This region contains *Hmr^mel^* (*hybrid male rescue*), which is known to induce hybrid male lethality in *mel/sim* and *mel/mau* crosses; as well as *CG11160^mel^*, an allele suggested to be lethality-inducing in a previous mapping effort in *mel/mau* hybrids [38]. With the current mapping resolution, however, it is not possible to discern whether there is a single lethal allele or whether both alleles are lethal. Surprisingly, a larger duplication that contains the region between 8D and 9E (8D9-8E4; 9E2; Stock: 29782) does not cause lethality in either attached-*X* background. This result suggests that the large duplication might mask recessive alleles on *X^san^* that are required for the lethal epistatic interaction.

To exclude dominant lethal effects of the *Dp(1;Y)* duplications assayed, we examined their effects in intraspecific crosses. We assayed two more crosses: *D. melanogaster C(1)RM, Dp(1;Y)*×*D. melanogaster* Malawi-6-3 and *D. melanogaster* Malawi-9-2. The male and female progeny counts produced in these crosses are shown in Table S8. We did not find any fragment of *X^mel^* that cause embryonic, larval or pupal lethality when carried by *Y^mel^* in these intraspecific crosses in either females or males, indicating no dominant epistatic interactions or lethal dosage effects of the *X^mel^* pieces attached to the *Y^mel^* chromosome are present in the pure species background.

### Rate of evolution of hybrid incompatibilities

We assessed whether the number of dominant alleles causing hybrid inviability followed the predictions of the snowball effect hypothesis of hybrid incompatibilities: the rate of accumulation of hybrid incompatibilities grows faster than linearly with divergence [3], [21]. We used previous genome-wide estimates of the number of silent substitutions per site between *D. melanogaster* and *D. santomea* (Ks*_mel-san_* = 0.24, [22]) and between *D. melanogaster* and *D. simulans* (Ks*_mel-sim_* = 0.11, [22]). Since *D. mauritiana* and *D. simulans* are equidistant from *D. melanogaster* [63], we assumed Ks*_mel-mau_* = Ks*_mel-sim_* = 0.11. Finally since we did not know the number of non-synonymous substitutions between the attached-*X* stocks and the two tested *D. melanogaster* lines, we conservatively used the maximum value of π ever reported for *D. melanogaster* populations (π = 0.03, [64], [65]). We fitted two models, a model in which the number of hybrid incompatibilities grew linearly with molecular synonymous divergence and a model in which the number of incompatibilities grew as a quadratic function of synonymous divergence. This analysis is methodologically similar to previous attempts [22], [23] but focuses on the dominant partners of the negative epistatic interactions and not on recessive (hemizygous) partners. Our analysis shows that the quadratic model explains the data much better than the linear model (Quadratic model: AIC –Akaike Information Criterion-: 6.795; Linear model: AIC: 24.75, Figure S5). This result indicates that the number of lethal alleles on *X^mel^* in crosses between species with different divergence times follows the snowball theory and suggests that, similar to observations of recessive hybrid lethal alleles, the relative frequency of dominant hybrid lethal alleles also increases faster than linearly in hybrid crosses.

## Discussion

### *D. melanogaster/D. santomea* hybrid males are viable if they carry the *X*-chromosome from *D. santomea*

The viability of male *D. melanogaster/D. santomea* hybrid males provides several clues to the broader genetic architecture of hybrid inviability between these two species. In general, the possibility of producing these males indicates that there are no lethal incompatibilities between *X^san^* and *Y^mel^*, or between *X^san^* and the *mel* cytoplasmic elements. More specifically, these hybrid males allowed us to address the question of whether *X^mel^* harbors dominant alleles involved in hybrid inviability.

The production of *X^san^/0* and *X^san^/Y^mel^* hybrid males also sheds some light on previous results generated by pole cell transfers of *D. yakuba* (the sister species of *D. santomea*) into *D. melanogaster* mutants that carried no pole cells [66]. Sanchez and Santamaria [66] reported that it was possible to produce progeny between *D. melanogaster* females with gametes carrying the genome of *D. yakuba* (as a result of pole cell transfer) and *D. melanogaster* males. This hybridization is equivalent to a ♀ *D. yakuba*×♂ *D. melanogaster* cross, which has never succeeded with wild-type animals. These crosses produced viable hybrid individuals of both sexes. The *yak/mel* and *mel/san* male hybrids are not directly comparable at the cellular level because the two kinds of hybrids have different cytoplasmic elements. Nonetheless, there are certain elements that the two hybrids share. Both of them have a haploid *D. melanogaster* autosomal genome. Most relevant to the present analysis, the hybrid males from both crosses carry an *X*-chromosome from either *D. santomea* or *D. yakuba* and lack the *X*-chromosome from *D. melanogaster*. These results indicate that the architecture of hybrid inviability in hybrids between *D. melanogaster* and *D. santomea* might be similar to that in hybrids between *D. yakuba* and *D. melanogaster*; both crosses produce viable heterozygote females (in which recessive alleles on one *X*-chromosome are effectively masked by the other), and males *only* with the non-*mel X*-chromosome (although the cross that could produce *X^mel^Y^yak^* males has not successfully been attempted). Since *D. santomea* and *D. yakuba* are closely related, with a divergence time of 0.4 million years [67], [68], it is reasonable to expect that a substantial proportion of the loci that interact with *mel* alleles to cause hybrid inviability originated along the lineage leading to both *san* and *yak* and are shared between the two species.

### *D. melanogaster/D. santomea* hybrid males and some hybrid females suffer from dominant or semi-dominant lethality alleles on *X^mel^*

The difference between the hybrid males from the ♀ *D. melanogaster*× ♂ *D. santomea* cross (*X^mel^/Y^san^*) and the males from the *D. melanogaster* ♀ *C(1)RM/Y^mel^*× ♂ *D. santomea* cross (*X^san^/Y^mel^*) is the identity of the sex chromosomes the males carry: in the former case they carry *X^mel^* and in the latter they carry *X^san^*. Hybrid males from both crosses carry a set of autosomes from each of the parental species. These results indicate that *X^mel^* carries deleterious alleles that cause lethality in *mel/san* hybrid males, but that *X^san^* does not have the same lethality effect.

The developmental defects that the hybrid males show in the presence of some *X^mel^* fragments indicate that one (or more) genetic factors on *X^mel^* lead to the characteristic abdominal ablation phenotype in *mel/san* hybrids [53]. This phenotype is present in both hybrid males that have the *X^mel^* and in hybrid females with two attached *X^mel^* and no *X^san^*, and in a small but consistently observed fraction of the *X^mel^/X^san^* females. Therefore the phenotypic determinant must be dominant or semi-dominant on *X^mel^*. We mapped the determinant(s) to the tip of *X^mel^* (between cytological bands 3A and 3D). In the absence of this determinant element, other *X^mel^*-linked elements can cause hybrid inviability as well as different embryonic patterning defects at later developmental stages (Figure 4, Figure S4).

The sex specific lethality found in *mel/san* hybrid males is distinct from any known Mendelian sex-specific lethal mutations previously discovered in a pure species; it is dominant/semi-dominant and only partially rescued by the presence of the *X^san^* chromosome. Our report shows that like hybrid sterility, hybrid inviability can be sex specific because the genetic background of females is different from that of males [30]. Namely, females, or more generally individuals from the homogametic sex, might experience negative epistatic interactions between sex chromosomes, a type of epistasis that the heterogametic sex will usually not experience. On the other hand, the heterogametic sex will suffer more from deleterious recessive alleles on the *X*-chromosome. An alternative scenario that also leads to sex-specificity in hybrid inviability is the effect of sex-specific lethal alleles. Several mapping efforts in *D. melanogaster* have uncovered the existence of alleles that cause lethality in only one sex. The majority of male sex lethal (MSL) mutations discovered via mutagenesis screening are both autosomal and recessive [69], [70] and are involved in the regulation of dosage compensation in pure species males [71], [72]. The expression of all dosage compensation genes, is negatively regulated by the *X*-linked sex determining master gene, *Sex-lethal* (*Sxl* [reviewed in 71]). *SXL* is a binary switch gene that controls all aspects of *Drosophila* sexual dimorphism. In wild-type animals, *SXL* is active in females and inactive in males [73]. Notably, different *Sxl* alleles can induce male or female specific lethality [73]–[75], which makes *Sxl* one of the prime candidates to cause sex-specific lethality. In our screening, none of the lethal duplications overlap with *Sxl* (cytological position in the *X^mel^*-chromosome: 6F3–6F5), suggesting that the presence of fragments containing *Sxl* ^mel^ does not to lethal doses of the feminizing allele. In *Drosophila* hybrids, some interplay between the dosage compensation and sex determination factors might contribute, or at least be correlated, to the inviability of hybrid males [61], [62], [76]. Nonetheless, the literature currently does not pose a consensus model for how these deleterious effects manifest in lethality [61], [76].

### *D. melanogaster/D. simulans* and *D. melanogaster/D. mauritiana* hybrid males have fewer dominant DMI partners on *X^mel^*

The relative density of dominant factors that cause hybrid inviability on *X^mel^* in *mel/sim* and *mel/mau* hybrids is lower than that in *mel/san* hybrids. This is to be expected given that the phylogenetic distance between these species is lower than between *mel* and *san* (*K_s_* between *mel* and *sim* = 0.11; *K_s_* between *mel* and *san* = 0.24, [22]).

We did not find any dominant *X^mel^*-linked alleles that cause embryonic hybrid lethality in either *mau or sim* hybrids, but found two regions that cause larval and pupal inviability (Figure 6 and 7). These duplication-mapping results are congruent with the mapping effort conducted by Cattani and Presgraves [41] which found that a single allele on *X^mel^*, *CG11160*, might cause pupal lethality in *mel/mau* hybrids, but only when it is allowed to interact with recessive alleles in the heterochromatic region of *X^mau^*. In this study, two overlapping duplications of *X^mel^* region containing both *CG11160* and *Hmr* causes inviability in *mel/mau* and in *mel/sim* hybrid males, but the current mapping resolution does not allow us to distinguish between their potential contributions to hybrid inviability.

### Epistatic interactions between *X*-chromosomes might contribute to hybrid breakdown

Sawamura and Yamamoto [40] were the first to report that it is possible to have negative epistatic interactions between *X*-chromosomes that lead to inviability in hybrid females. Cattani and Presgraves [41] took a systematic approach and tiled a large proportion of the *X*-chromosome and identified one dominant lethal allele on *X^mel^*. In this report, we demonstrate that these interactions might not be as rare as previously thought [77] and might constitute an understudied phenomenon in the genetics of hybrid breakdown.

Six key findings, from this study and others, shed light on the causal role of sex chromosomes in inviability in interspecific crosses involving *D. melanogaster* and *D. santomea*: *i)* hybrid females that carry one *X*-chromosome from each species (*X^mel^/X^san^*) are usually, but not always, viable [22], *ii*) hybrid males carrying *X^mel^/Y^san^* are inviable [53], *iii)* hybrid males carrying *X^san^/Y^mel^* are viable (Figure S1), *iv)* hybrid females carrying *X^mel^X^mel^/Y^san^* are inviable (Figure 2), *v)* hemizygosity for 13 different regions of *X^san^* causes inviability in hybrid females as revealed by deficiency mapping [22], and *vi)* the presence of 12 isolated *X^mel^*-linked regions cause inviability in *mel/san* hybrid males (9 of them induce embryonic lethality and 3 of them induce pupal lethality; Figure 5, Figure S6). These results indicate that the two *X*-chromosomes are heavily implicated in the inviability of *mel/san* hybrids.

Our current results present a conundrum in light of the discovery of viable hybrid *X^san^/Y^mel^* males. The *X*-chromosome from *D. santomea* contains 13 chromosomal regions that cause hybrid inviability when hemizygous in females [22], but apparently allow for viable hemizygous hybrid males in the absence of any *X^mel^* homologous alleles. The same phenomenon (alleles causing inviability in hemizygous females are not lethal in hemizygous males) is observed in *mel/sim* and *mel/mau* hybrids as well. Additionally, pieces of *X^mel^* lead to inviability in hybrid males carrying the paternal species *X*-chromosome, but an intact *X^mel^* in hybrid females carrying the paternal species *X*-chromosome does not. All of these results can be explained if epistatic interactions between alleles on the *X*-chromosomes contribute to hybrid breakdown. In the males carrying fragments of *X^mel^*, all the recessive alleles from *X^san^* that are not masked by the *mel Dp(1;Y)* will be fully expressed, but unlike *X^san^/0* hybrid males, these males will suffer from the epistatic interactions between the fragment of *X^mel^* carried on *Dp (1;Y)* and the exposed recessive alleles from *X^san^*. *X^mel^X^san^* hybrid females, on the other hand, will not experience these epistatic interactions because when the complete *X^mel^* is present, it will mask *all* the recessive alleles from *X^san^*.

The viability of *mel/san* hybrid males was hypothesized to be evidence against the existence of alleles involved in hybrid inviability on the *X* chromosome of *D. santomea* discovered by lethal deficiencies in hybrid females [77], [78]; in hybrid males, these alleles would be unmasked and would be free to interact in DMI with dominant alleles on *D. melanogaster* autosomes. However, in hybrid males, these alleles would not act in DMI with alleles on *X^mel^*. Here we report the existence of dominant hybrid lethal alleles on *X^mel^* that interact with alleles on *X^san^*. This in turn provides a simple explanation of why *X^san^*-carrying hybrid males survive to adulthood while partly hemizygous hybrid females do not. If the epistatic partner of *X^mel^*-linked lethal dominant alleles resided in the *X^san^*-chromosome, then hybrid females would be inviable if the *X^san^* alleles are dominant and viable if they are recessive; they cause inviability only when not masked by an *X^mel^* counterpart. The position of the regions identified in this report, and the position of the regions identified by deficiency mapping are shown in Figure S6. It is provocative to think these alleles could reside in the chromosomal regions uncovered by Matute et al. [22] but this hypothesis has not been tested.

Our approach to identifying *X^mel^*-linked alleles by using large fragments of *X^mel^* is conservative and underestimates the relative frequency of these elements in two ways. First, each duplication could include more than one allele involved in hybrid inviability. Second, if it is true that *X^mel^* lethal alleles require recessive DMI partners on *X^san^*, then large *X^mel^* duplications will mask recessive components that are required to cause hybrid inviability (see Caveats). This in turn means that there could be more alleles on *X^mel^* that contribute to hybrid inviability that remain to be identified.

### Caveats

The results here presented come with five caveats. First, the lethal alleles identified in the duplication mapping screen could be the result of hybrid-specific lethal dosage effects caused by the presence of *Dp (1;Y^mel^)* in *mel/san* hybrid males. However, simple dosage effects are unlikely to be involved in inviability as no lethal trisomies in pure-species females, or lethal dosage effects in hybrid males were observed in intraspecific crosses. It is possible, however, that hybrid individuals are more susceptible to dosage effects than pure-species individuals. If such dosage effects exist, they must therefore be specific to the hybrid background and mediated by the negative epistasis arising in the hybrids. In that case they would constitute a subcase of DMI mediated by gene dosage rather than physical interactions. The role that dosage compensation can have in *mel/san* hybrid males that also carry a *X^mel^*-chromosome duplication is beyond the scope of this study (but see [67] for a full genetic test of the role of dosage compensation in *mel/sim* hybrid inviability).

The second caveat is that this method only presents a minimum estimate of *X^mel^* –linked lethal alleles involved in hybrid inviability. As described above, our mapping used large duplications and each chromosomal segment might include more than one dominant lethal allele, and might mask recessive DMI alleles on *X^san^*. A third caveat is that in the male viability analyses, we only counted duplications that caused a *total* reduction of male viability. Since duplication mapping does not have internal controls, we did not count *X^mel^* regions that cause only a partial reduction in viability. We used an arbitrarily stringent 10% viability cut-off to classify alleles as lethal. It is worth nothing that more sophisticated and quantitatively-framed analyses are possible but they will require more statistical power (i.e., more replicates per cross) than that presented here (described in Methods). A fourth caveat is that all the interactions we detected occur in a genetic background in which besides the *mel Dp (1;Y)* duplication, the regions around *yellow* (1Lt-1B5), and *X^mel^* heterochromatin elements are also present (20F3- h29). The presence of these components precludes the study of *X^san^* recessive alleles in these chromosomal regions.

The final caveat is that since we could not produce hybrid males without *Y^san^*, we could not explore the effect of this chromosome on hybrid viability. This is important because *X^mel^*/*Y^san^* males and hybrid females with attached-X *X^mel^X^mel^*/*Y^san^* both die at the embryonic stage. We have shown that *X^mel^* carries dominant lethals that could cause hybrid inviability in these animals, but we have not tested whether *Y^san^* contributes to hybrid inviability. Hybrid inviability could be caused by negative epistasis between *Y^san^* and *X^mel^*, between *Y^san^* and the *D. melanogaster* autosomes, or between *Y^san^* and the *D. melanogaster* cytoplasmic factors. The study of *Y^san^* would require the development of a *D. santomea* stock with both a attached *X*-chromosome and a *Y*-linked *X* duplication [i.e, *C(1)RM/C(1;Y)*], as has been done for *D. melanogaster* [79], [80] and *D. simulans* [81]. Females from this stock could be crossed with *D. melanogaster* males. The cross would produce *X^san^Y^san^/Y^mel^* embryos whose viability could be compared with that of *X^san^/Y^mel^* embryos (assuming that carrying two *Y*-chromosomes is not deleterious in the hybrids). So far, though, *D. santomea* females have shown complete premating isolation from *D. melanogaster* males, indicating that even if the *Y*-linked *X* duplications existed, premating isolation might hamper the possibility of studying this hybridization.

### Conclusions

The *X*-chromosome has a large effect on hybrid breakdown, especially in the heterogametic sex. Orr [30], for example, proposed that since the heterogametic sex is subject to the dominant and recessive deleterious effects of the hemizygous sex chromosome, *Drosophila* males are more prone to hybrid inviability and sterility. This, however, does not mean that sex-linked alleles do not affect hybrid females (the homozygous sex). Nonetheless, the identification of dominant hybrid inviability alleles on the *X*-chromosome has been challenging (but see [40], [82]–[84]). Our results provide a fine-grained snapshot of the localization and relative frequency of dominant alleles involved in DMI in different hybrid crosses on the *D. melanogaster X*-chromosome; we infer that they interact with recessive alleles on *X^san^* in the homogametic females since these dominant alleles do not cause inviability in hybrid males. Furthermore, these epistatic interactions are lineage specific, since crosses between *D. melanogaster* and different species display different numbers of alleles that cause hybrid inviability in different regions. In accordance with the snowball effect theory for the rate of evolution of hybrid incompatibilities, more *X^mel^*-linked alleles are involved in DMI in *mel/san* hybrids than in *mel/sim* or *mel/mau* hybrids. These results are complementary to the previous results showing the snowball effect holds true in recessive allele partners in *Drosophila* [22]. This report suggests that snowball theory also holds for the dominant components of DMI.

It is likely that future studies using finer mapping techniques with more and smaller duplications will find more hybrid incompatibility alleles on *X^mel^* than we have. Regardless of the actual abundance of hybrid lethal alleles on *X^mel^*, the results shown here provide evidence of negative epistatic interactions between *X*-chromosomes of hybrids that can affect the homogametic sex, but not the heterogametic sex. This reveals the existence of an even larger *X*-effect in hybrid breakdown than was previously ascertained.

## Materials and Methods

### *Drosophila* stocks

We recently discovered that crossing *mel* attached-*X* females [85]–[87] to *D. santomea* males produces viable offspring, all of which are sterile males carrying the *X^san^* chromosome. The attached-*X* females can also carry a *Y^mel^* chromosome, but remain morphologically female since sex is determined by the *X*:Autosome ratio [88], and produce attached-*X* gametes and *Y^mel^* gametes. When these females are crossed with *D. santomea* males, the viable F_1_ hybrid males will carry an *X^san^* and a *Y^mel^*, Figure 1, Panel A.) Attached-*X mel* females produce viable hybrid males when crossed to three other different species, *D. simulans*, *D. sechellia*, and *D. mauritiana* [31]. For all experiments in this report (unless explicitly stated, we used two different attached-*X* stocks: *C(1)RM* (Compound (1) Reversed Metacentric; two *X* chromosomes in normal sequence attached proximally to the same centromere; [86], [89], [90] and *C(1)DX* (Compound (1) Double X, Muller [87], [91]). We took advantage of the *mel* panel of small *X*-chromosome fragments attached to the *Y*-chromosome (*Dp(1;Y)*s) generated by Cook and colleagues [46], to study the genetic basis of hybrid inviability in three of these hybrid males: *mel/san*, *mel/sim*, and *mel/mau*. Briefly, this duplication panel was generated by first creating *X* inversions using FLP-FRT recombination on attached-*XY* chromosomes. These inverted *XY* compound chromosomes are then irradiated to induce large internal *X* deletions. The resulting chromosome contains a medial *X* segment flanked by the tip of the *X* (1Lt;1B5), which carrys the *y^+^* allele and the *X* centric heterochromatin region (20F3-h28; h28-h29) adjacent to the fused *Y* [52]. All the used stocks are listed in Table S1.

We first crossed *mel C(1)RM* females to *mel Dp(1;Y)* males and the virgin female progeny [*mel C(1)RM/Dp(1;Y)*] were then crossed to *D. santomea* males. This cross yielded only F_1_ hybrid males harboring an *X^san^* and a [*Y^mel^*, *Dp(1;Y*)] chromosome. Figure 1 (Panel B) shows the crossing scheme. As all the *Dp(1;Y)* chromosomes also carry *y+* this assay only allows the testing of the effect of a *mel* gene when a *y^[mel]+^* gene is also present. The [*C(1)RM, y w f*/*Dp(1;Y)*, *y^+^*] females were used for both interspecific and intraspecific crosses. For all hybrid crosses involving *D. santomea*, we used a synthetic line, SYN2005. This outbred line was constructed by combining isofemale stocks and kept in large numbers since its initiation [92], [93]. For crosses involving *D. simulans*, we used the synthetic line *D. simulans* Florida City [94]. For crosses involving *D. mauritiana*, we used the SYN stock, a synthetic stock generated by O. Kitigawa [95], [96]. For the intraspecific crosses, we used two different *D. melanogaster* inbred lines (37,289: Malawi-6-3 and 30,857: Malawi-9-2; [97], [98]). These two lines make part of the *Drosophila* Population Genomics Project effort to characterize genetic variation in *D. melanogaster* (http://www.dpgp.org/). Figure 1 shows the two generations involved in the described crossing scheme.

We followed an identical crossing scheme for heterospecific crosses involving *C(1)DX, Dp(1;Y)*. Hybrid inviability can be the result of specific mutations in the *D. melanogaster* background of the line used to study inviability. To assess whether this was the case, we repeated all the crosses involving *C(1)RM, Dp(1;Y)* but instead we used an alternative attached-*X* chromosome stock: *C(1)DX*. The rationale behind these experiments is that if the lethality induced in the hybrid males is due to the duplication of the *X^mel^* alleles attached to *Y^mel^*, and not an artifact in the background, then the lethality should be reproducible in a different genetic background, namely, the results should be equivalent, or at least similar in experiments using the two types of attached-*X* chromosome. *C(1)RM, Dp(1;Y)* and *C(1)DX, DP(1;Y)* carrying the same duplication have in average only 25% of genetic material.

All *D. melanogaster* lines (homo- and hetero-compound chromosomes, mutant stocks, and natural lines from DPGP) were obtained from the Bloomington Stock Center (http://flystocks.bio.indiana.edu/) and are listed in Tables S1 and S2.

### Embryonic lethality

*D. melanogaster* females were housed for three days with males from each of the studied species in 8-drams corn-meal vials to allow for insemination. At the end of this period the flies were transferred without anesthesia to a lightly yeasted apple juice plate collection cup. Females were allowed to oviposit for 24 hours and then they were changed to a new plate. After removal from the collection cup, the plate was incubated for 24 hours at 25°C and scored for hatched vs. dead embryos in a protocol similar to Gavin-Smyth and Matute [53].

### Metafemale embryonic phenotypes and cuticle preparation

To distinguish the embryonic lethal phenotypes of the *mel C(1)DX/Y^san^* females from the *C(1)DX/X^san^* metafemales, we constructed a *C(1)DX*, *y^1^ w^1^ f^1^* stock homozygous for a *Sxl::GFP* reporter construct on the third chromosome (Stock number: 24105; w*; P{Sxl-Pe-EGFP.G}G78b, [99]). These females were crossed to *D. santomea* males. Overnight depositions of these crosses were collected and sorted for *GFP* expression after four hours of incubation. The *Sxl::GFP^+^* (female embryos) were then incubated for a further 24 hours. Embryos that failed to hatch were prepared for cuticle mounting as described in Gavin-Smyth and Matute [53]. Since *y^1^* alleles of the homo-compound *X^mel^* chromosomes (either *C(1)RM* or *C(1)DX*) are rescued by the wild-type *D. santomea yellow*, we could identify female and male embryos. Metafemale cuticles have wild-type pigmentation of the larval mouth hooks, while the *X^san^Y^mel^* remain *yellow^−^*.

### Larval and pupae lethality

We transferred L_1_ larvae from the deposition apple juice plates to 8-dram corn-meal fly food vials. All these larvae were males as evidenced by the color of their mouthparts. We then counted how many larvae molted to pupae, and how many pupae eclosed into adults and how many failed to eclose. Pupae were only assigned as dead once they had been formed for at least 14 days and had started to necrotize.

### Relative density of dominant lethals

Once the whole *X^mel^* was tiled for the five crosses, we determined the minimal number of lethal alleles in *X^mel^* for each cross. We fitted a general linear model for each developmental stage for each species in which the relative viability was the response, and the genotype (identity of the duplication) and the genetic background (i.e., the type of attached-*X* stock used in the crosses) were the fixed effects. Nonetheless, the residuals from these all these models deviate from the normality assumptions required for linear models (Figure S7). These deviations from normality persisted after multiple attempts of transformation and precluded the possibility of using linear models. We then resorted to nonparametric tests and used a Kruskal-Wallis test. We tested the efficacy of these tests using the crosses between *mel* females and *sim* males. This cross was chosen because it has been previously established that an allele residing in the *X*-chromosome (precisely in 9D4) causes lethality at the larval stage. We compared the viability of each of these *C(1)RM, Dp(1;Y)* crosses with that of control crosses (*C(1)RM* results pooled with *C(1)DX* results). We detected no regions that significantly reduced the viability in any developmental stage indicating that our experimental design has not enough power to detect lethal alleles using nonparametric tests even though there were *X^mel^* regions that clearly cause hybrid lethality. We thus had to resort to a qualitative approach and classified lethal alleles as those that caused lethality on more than 90% of their carriers. Once lethal duplications were identified, we determined the minimal number of segments that lead to lethality. If two lethal duplications overlapped, then we assumed that the cause for lethality was shared between the two duplications. This approach, also used in deficiency mapping [22], [38], [48], is conservative and tends to underestimate the number of hybrid incompatibilities. For the sake of clarity, we only report crosses which produced progeny for the three interspecific hybridizations. All the attempted intraspecific crosses produced progeny.

### Viability and longevity

To measure viability in different interspecific crosses, we set up collection cups and counted all the fertilized embryos (both dead and hatched). We used *C(1)DX*, *y^1^ w^1^ f^1^, +/+, Sxl::GFP/Sxl::GFP* and *C(1)RM*, *y^1^ w^1^ f^1^, +/+, Sxl::GFP/Sxl::GFP* females and crossed them to males from the five assayed stocks (three intraspecific and two intraspecific crosses). After overnight depositions, we collected the *Sxl::GFP^−^* (male embryos), transfer them to a 1 cm×1 cm filter paper square, which in turn was transferred to an eight-dram vial with corn-meal food. Vials were tended daily and for each vial (replicate), we counted how many adults emerged. Male viability was calculated as the proportion of *Sxl::GFP^−^* that reached adulthood. The effects of the *mel* attached-*X* genetic background viability, and of the presence of *Y^mel^* within each set of interspecific crosses were analyzed with a two-way ANOVA that took the form:
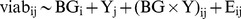
Where viab_ij_ is the viability per replicate, *BG_i_* was the genetic background of the *mel* attached-*X* stock of the mother, *Y_j_* was whether the males carried a *Y^mel^* or not, *(BG×Y)_ij_* was the interaction between the two fixed effects, and E_ij_ was the error term. All statistical analyses were done using R [100]. P-values were corrected to control for multiple comparisons following a Sidak's multiple comparison correction [101].

We also compared the longevity of three interspecific hybrid males (*mel/san*, *mel/sim* and *mel/mau*) with virgin males from four pure species (*D. melanogaster*, *D. mauritiana*, *D. santomea*, and *D. simulans*). We measured the longevity of 120 males per genotype, split into ten different vials (12 males per vial). We fitted a linear mixed model for each set of interspecific crosses which took the form:
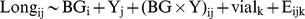
where *Long_ij_* was the longevity of each individual, *BG_i_* was the genetic background of the *D. melanogaster* attached-*X* stock of the mother, *Y_j_* was whether the males carried a *Y^mel^* or not, *(BG×Y)_ij_* was the interaction between the two fixed effects, vial was a random effect, and E_ijk_ was the error term. P-values were corrected for multiple comparisons in the same manner as described above (viability linear models). (Nonparametric tests showed similar results to the linear model.)

Finally, we assessed the effect of the *Y^mel^* chromosome on the number of sex comb teeth in six kinds of interspecific hybrid males: *mel/san X^san^/0*, *mel/san X^san^/Y^mel^*, *mel/sim X^sim^/0*, *mel/sim X^sim^/Y^mel^*, *mel/mau X^mau^/0*, and *mel/mau X^mau^/Y^mel^*. For this analysis we only used hybrid males produced in crosses with *C(1)RM/Y^mel^*, or *C(1)RM/0* females. There were three comparisons per trait, one for each paternal species, for a total of six comparisons. All raw data and analytical software are available from the Dryad Digital Depository (https://datadryad.org/): doi:10.5061/dryad.ft6r5.

## Supporting Information

Figure S1 Morphological characters of *mel/san* hybrid males with an *X^san^*. A. Sex combs in *X^san^*/0 males. B. Sex combs in *X^san^*/*Y^mel^* males C. Distribution of number of sex comb teeth in *D. melanogaster*, *D. santomea*, and the hybrid males (both *X^san^/0* and *X^san^/Y^mel^*). The differences between pure species, and between pure species and F_1_ hybrids are significant (Wilcoxon rank sum test with continuity correction data: *D. melanogaster* vs. *D.santomea*, W = 38,696, P<2.2×10^−16^; *D. melanogaster* vs. F_1_ hybrids: W>23,813.5, P<1.145×10^−4^; *D. santomea* vs. F_1_ hybrids: W>4,585.5, P<2.2×10^−16^). The differences between F_1_s are not significant (Wilcoxon rank sum test with continuity correction data: *X^san^/0* vs. *X^san^Y^mel^*, W = 21,687, P = 0.113). D. Abdominal pigmentation in *X^san^*/0 males. E. Abdominal pigmentation in *X^san^*/*Y^mel^* males.
(PDF)

Figure S2 Longevity and viability of *mel/san* (*X^san^/0*) hybrid males compared to other *Drosophila* hybrid males and to virgin males of the parental species. A. Viability of hybrid males is equivalent in all the hybrid crosses. (Viability of pure species was calculated for both sexes combined.) B. Hybrid males from the three types of interspecific crosses can live as long as virgin males from their parental species and the presence/absence of a *Y^mel^* chromosome has no effect in any of the two traits. Similarly, the genetic background of the attached-*X* stock used for the crosses had no effect in either trait in none of the three interspecific types of crosses. White: pure species; red: *mel/san* hybrid males; blue: *mel/sim* hybrids; yellow: *mel/mau* hybrids. A. *mel*, B. *san*, C. *mau*, D. *sim*, E. *C(1)RM: X^san^/Y^mel^*, F. *C(1)RM*: *X^san^*/0, G. *C(1)DX*: *X^san^*/*Y^mel^*, H. *C(1)DX*: *X^san^*/0, I. *C(1)RM*: *X^mau^*/*Y^mel^*, J. *C(1)RM*: *X^mau^*/0, K. *C(1)DX*: *X^mau^*/*Y^mel^*, L. *C(1)DX*: *X^mau^*/0, M. *C(1)RM*: *X^sim^*/*Y^mel^*, N. *C(1)RM*: *X^sim^*/0, O. *C(1)DX*: *X^sim^*/*Y^mel^*, P. *C(1)DX*: *X^sim^*/0. All linear models shown in Table S7.
(PDF)

Figure S3 Relative frequency of developmental defects in *mel C(1)RM*×*san* crosses. The *mel/san* hybrid males from the *mel C(1)RM*×*san* cross carry a *X* ^san^ chromosome and the vast majority of them are viable. Females from this cross carry two fused *X^mel^* chromosomes (homocompound chromosome), do not survive embryogenesis and show abdominal defects similar to those observed in *mel/san* hybrid males that carry a *X^mel^* chromosome [53].
(PDF)

Figure S4 Developmental defects show different frequencies in the nine different lethal *mel/san* hybrid male genotypes. Each barplot corresponds to one of the nine regions (i.e., *Dp(1;Y)* duplications) that causes hybrid inviability in hybrid males and shows the relative frequency of abdominal ablations in hybrid individuals from both sexes. We measured three replicates for each *Dp(1;Y)* genotype (each replicate consisted of 20 *y^+^* and 20 *y^−^* cuticles). *y^−^* are dead female individuals, while y^+^ can be either hybrid males or hybrid metafemales. We assessed whether the *Dp(1;Y)* cause a deviation from the expected frequency of cuticular defects by comparing the average number of individuals that show abdominal ablations in each cross in each category (*y^−^* or *y^+^*) of the cross with the average frequency observed in *mel C(1)RM*×san crosses (controls, χ^2^ test, df = 1, Figure S3). We corrected for 18 comparisons comparisons using a Sidak's adjustment, (Significant P<2.8×10^−3^). The only significant P-value is highlighted with a red box. The cytological location of the chromosomal duplication is shown in the top of each histogram. A. *Dp(1;Y)BSC75* (*X*:2C1-3E4). B. *Dp(1;Y)BSC159* (*X*:4A5-4D7). C. *Dp(1;Y)BSC289* (*X*:5E1-6C7). D. *Dp(1;Y)BSC176* (*X*:7B2-7D18). E. *Dp(1;Y)BSC126* (*X*:11C2-11D1). F. *Dp(1;Y)BSC327* (*X*:11D5-11E8). G. *Dp(1;Y)BSC186* (*X*:12C1-12F4). H. *Dp(1;Y)BSC269* (*X*:12E9-13C5). I. *Dp(1;Y)BSC11* (*X*:16F6-18A7).
(PDF)

Figure S5 The number of *X^mel^*-linked alleles causing hybrid inviability is higher in the most divergent cross and follows the expectations of the snowball effect theory of hybrid incompatibilities. We used Ks (number of synonymous substitutions per site) as a proxy of divergence time and fitted the best linear (red) and the best quadratic model (blue) to the data. Overlapping points were jittered for clarity. We find the quadratic model has a much better fit than the linear model as evidenced by its lower AIC value.
(PDF)

Figure S6 Comparison of the deficiency mapping results from Matute et al. [22] with our results using duplication mapping. The developmental stage at which the *X^san^*-linked recessive alleles cause inviability has yet not been determined.
(PDF)

Figure S7 Quatile-quantile plots of the residuals of each of the nine attempted linear models (one for each developmental stage per interspecific cross: 3×3 = 9). Observed values (x-axis) are compared to the values that would be predicted in a normal distribution (y-axis). Dashed red lines give a point-wise 95% confidence interval around the fitted solid red line. Since all the cases showed strong deviations from normality, which in turn precluded the possibility of using linear models, we used a qualitative cut-off to detect lethal alleles.
(PDF)

Table S1 Viability and longevity of hybrid males in three interspecific crosses involving *D. melanogaster* using two different attached-*X* genetic backgrounds.
(DOCX)

Table S2 Neither the presence of a *Y^mel^* or the *D. melanogaster* genetic background of the attached-*X* stock significantly affected hybrid male viability or hybrid male longevity. Viability was analyzed with a full-factorial linear model, while longevity was analyzed with a linear mixed model (vial as a random effect, See Methods). Since there were six linear models, P-values were adjusted with a Sidak's multiple comparison correction; required P for significance <8.512×10^−3^).
(DOCX)

Table S3 Mutant lines used for this study. The details of the *D. santomea*, *D. simulans* and *D. mauritiana* are listed in the text. List of all the stocks (other than the *Y*-linked *X* duplication stocks) used in this study.
(DOCX)

Table S4 Duplication stocks used in this study. The table lists panel of *Y*-linked *X* duplication chromosome stocks obtained from the Bloomington Stock Center, stock number and genotype for all crosses attempted. Only 52% of the attempted crosses produced progeny in five attempts (shown in Table S6).
(DOCX)

Table S5 Insemination rates in all interspecific and intraspecific crosses. Insemination rates were measured by dissecting groups of 20 females and observing whether females had sperm in their reproductive tract (3 replicates per genotype). The mean number of mated females and the standard error are shown for each cross.
(DOCX)

Table S6 Viability rates for each developmental transition in the five crosses presented in this report (3 interspecific+2 intraspecific) in crosses involving *mel C(1)RM*. Averages were calculated with 3 replicates per cross. These data were used to generate Figures 3, 6, and 7. None of the *C(1)RM, Dp(1;Y)*×*mel* crosses showed decreases in viability at any developmental stage. The decrease in viability at the larval stage is consistent with the inviability of pure-species metafemales at the late larval stage.
(DOCX)

Table S7 Viability rates for each developmental transition in the three interspecific crosses presented in this report in crosses involving *mel C(1)DX*. These data, along with the data presented in Table S6, were used to generate Figures 3, 6, and 7. None of the *C(1)DX, Dp(1;Y)*×*mel* crosses showed decreases in viability at any developmental stage. The decrease in viability at the larval stage is consistent with the inviability of pure-species metafemales at the late larval stage.
(DOCX)

Table S8 Male and female progeny counts produced in crosses between *mel C(1)RM/Dp(1;Y)*×*mel* Malawi-6-3 and *mel* Malawi-9-2. No significant deviations from the 1∶1 ratio were observed in any of the assayed *Dp(1;Y)* duplications.
(DOCX)
