# Correction: Fine Mapping of Dominant X-Linked Incompatibility Alleles in *Drosophila* Hybrids

**DOI:** 10.1371/journal.pgen.1005558

**Published:** 2015-10-07

**Authors:** Daniel R. Matute, Jackie Gavin-Smyth

The legend for Fig 4 is incorrect. The correct legend is included here.

**Fig 4:**
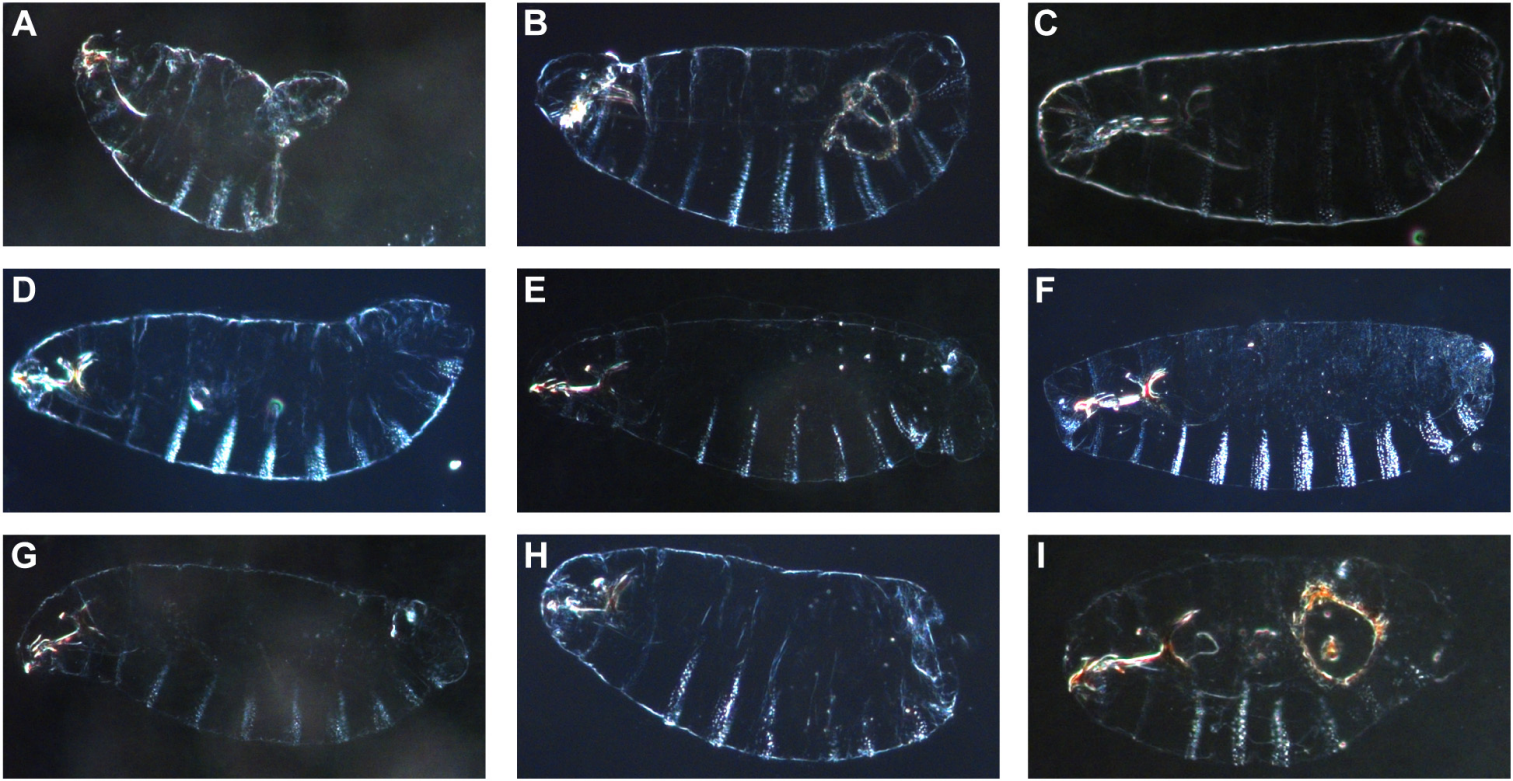
Cuticular defects observed in the nine lethal regions from the *D. melanogaster X*-chromosome. A. Males carrying the 3A–3D region show the abdominal ablation observed in *X^mel^/Y^san^* hybrid males and *X^mel^X^mel^/Y^san^* hybrid females. B–I. Hybrid males that carry any of the other eight embryonic lethal *X^mel^*-linked regions show different phenotypes and the abdominal ablation is more rare than in *X^mel^/Y^san^* males. The genotypes (and the cytological bands of the duplication) of each of the shown males are: A. *Dp(1;Y)BSC75* (X:2C1-3E4). B. *Dp(1;Y)BSC176* (X:7B2-7D18). C. *Dp(1;Y)BSC186* (X:12C1-12F4). D. *Dp(1;Y)BSC159* (X:4A5-4D7). E. *Dp(1;Y)BSC126* (X:11C2-11D1). F. *Dp(1;Y)BSC269* (X:12E9-13C5). G. *Dp(1;Y)BSC289* (X:5E1-6C7). H. *Dp(1;Y)BSC327* (X:11D5-11E8). I. *Dp(1;Y)BSC11* (X:16F6-18A7).

## References

[1] Matute DR, Gavin-Smyth J (2014) Fine Mapping of Dominant X-Linked Incompatibility Alleles in *Drosophila* Hybrids. PLoS Genet 10(4): e1004270 doi:10.1371/journal.pgen.1004270 24743238 10.1371/journal.pgen.1004270 PMC3990725

