# Supplementary figures and images for: Fine Mapping of Dominant X-Linked Incompatibility Alleles in Drosophila Hybrids
Source: PLoS Genet. 2014 Apr 17;10(4):e1004270. doi: 10.1371/journal.pgen.1004270 (PMC3990725; doi:10.1371/journal.pgen.1004270)

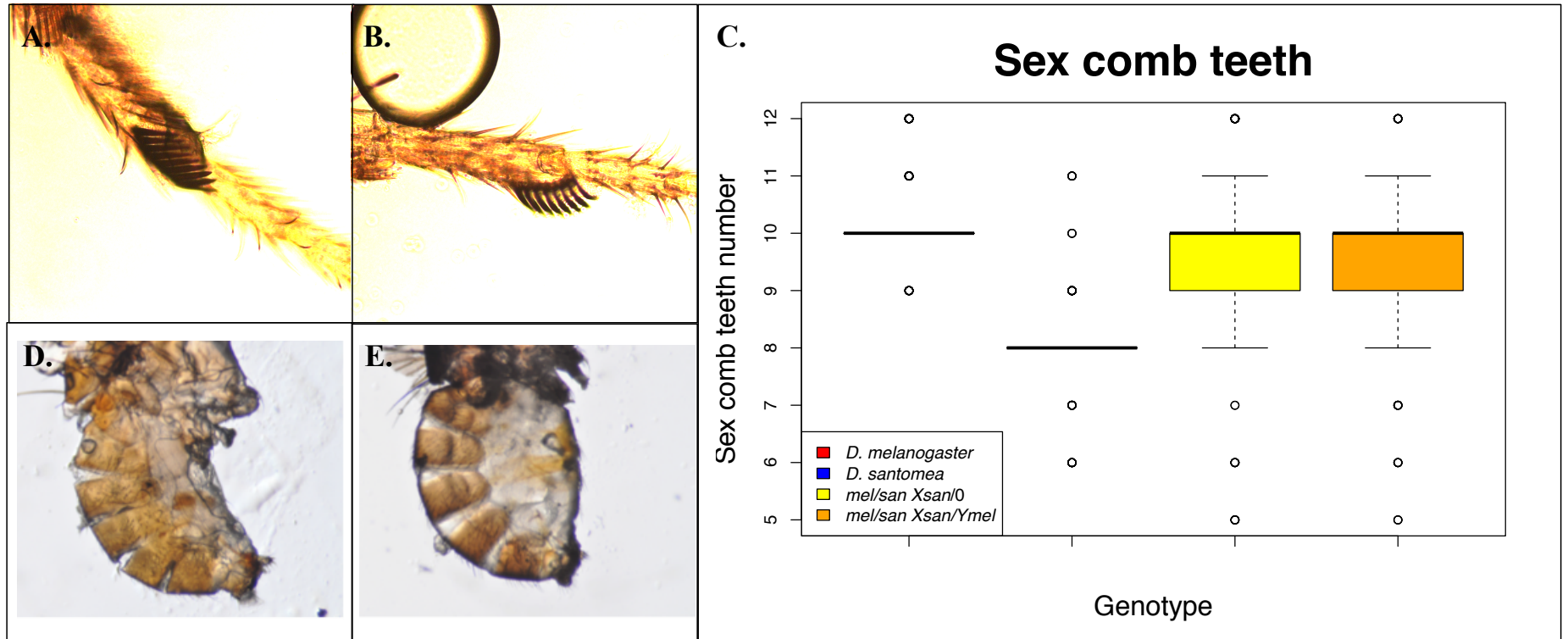

**SUPPLEMENTARY FIGURE 1**

Supplement: Figure S1 — Morphological characters of mel/san hybrid males with an Xsan. A. Sex combs in Xsan/0 males. B. Sex combs in Xsan/Ymel males C. Distribution of number of sex comb teeth in D. melanogaster, D. santomea, and the hybrid males (both Xsan/0 and Xsan/Ymel). The differences between pure species, and between pure species and F1 hybrids are significant (Wilcoxon rank sum test with continuity correction data: D. melanogaster vs. D.santomea, W = 38,696, P<2.2×10^−16^; D. melanogaster vs. F1 hybrids: W>23,813.5, P<1.145×10^−4^; D. santomea vs. F1 hybrids: W>4,585.5, P<2.2×10^−16^). The differences between F1s are not significant (Wilcoxon rank sum test with continuity correction data: Xsan/0 vs. XsanYmel, W = 21,687, P = 0.113). D. Abdominal pigmentation in Xsan/0 males. E. Abdominal pigmentation in Xsan/Ymel males. (PDF) [file pgen.1004270.s001.pdf]

**A. Viability**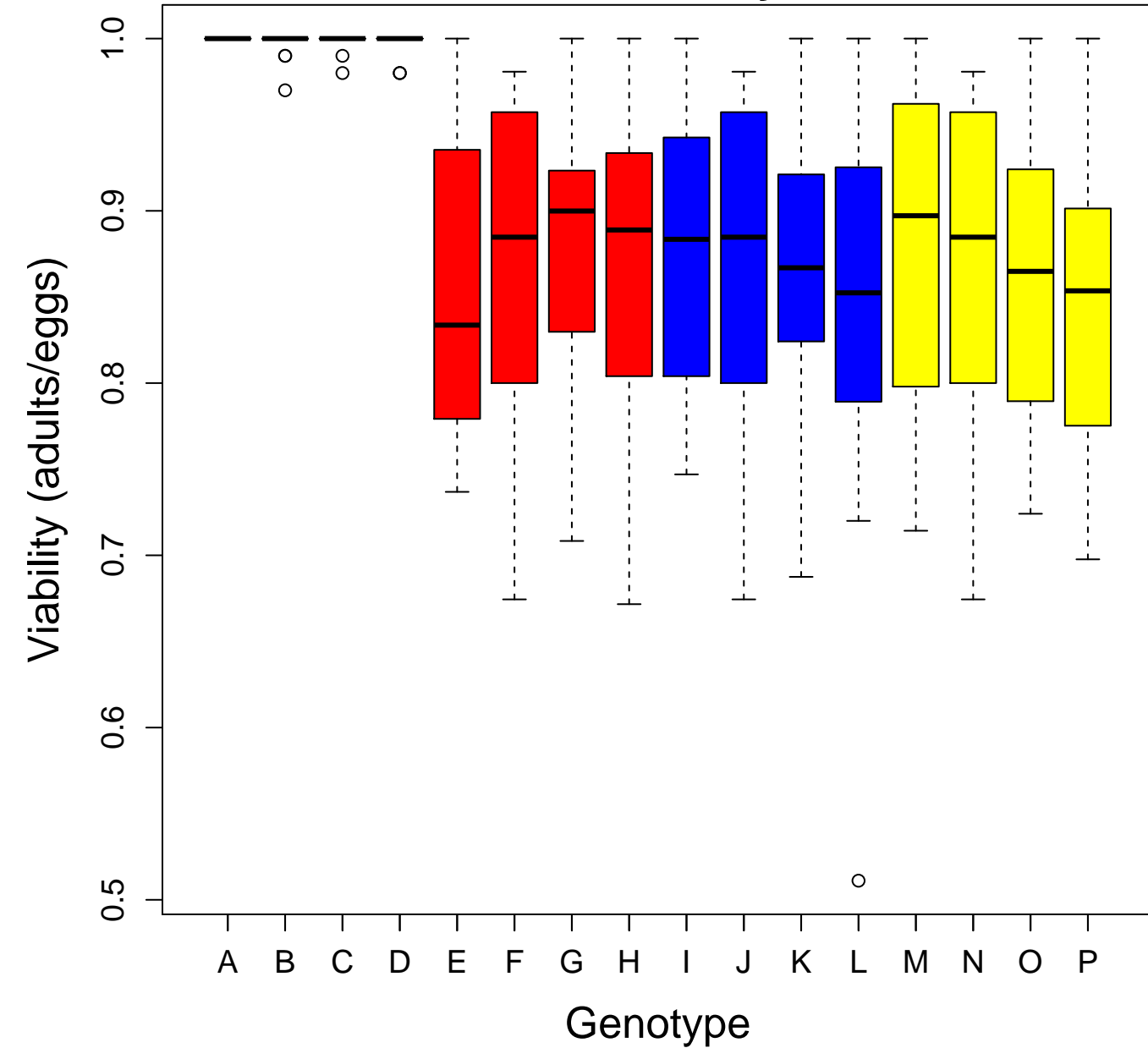**B. Longevity**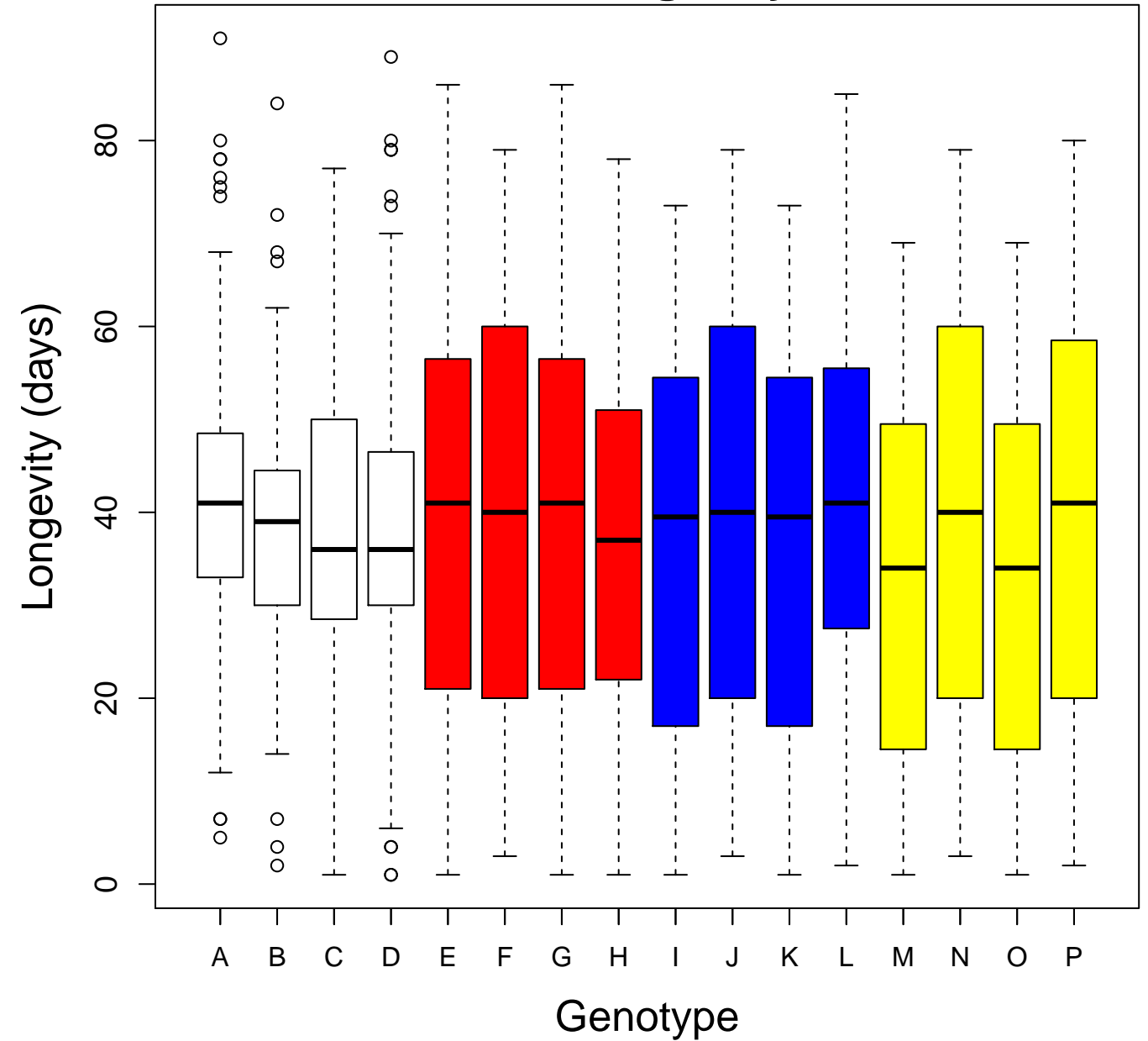

Supplement: Figure S2 — Longevity and viability of mel/san (Xsan/0) hybrid males compared to other Drosophila hybrid males and to virgin males of the parental species. A. Viability of hybrid males is equivalent in all the hybrid crosses. (Viability of pure species was calculated for both sexes combined.) B. Hybrid males from the three types of interspecific crosses can live as long as virgin males from their parental species and the presence/absence of a Ymel chromosome has no effect in any of the two traits. Similarly, the genetic background of the attached-X stock used for the crosses had no effect in either trait in none of the three interspecific types of crosses. White: pure species; red: mel/san hybrid males; blue: mel/sim hybrids; yellow: mel/mau hybrids. A. mel, B. san, C. mau, D. sim, E. C(1)RM: Xsan/Ymel, F. C(1)RM: Xsan/0, G. C(1)DX: Xsan/Ymel, H. C(1)DX: Xsan/0, I. C(1)RM: Xmau/Ymel, J. C(1)RM: Xmau/0, K. C(1)DX: Xmau/Ymel, L. C(1)DX: Xmau/0, M. C(1)RM: Xsim/Ymel, N. C(1)RM: Xsim/0, O. C(1)DX: Xsim/Ymel, P. C(1)DX: Xsim/0. All linear models shown in Table S7. (PDF) [file pgen.1004270.s002.pdf]

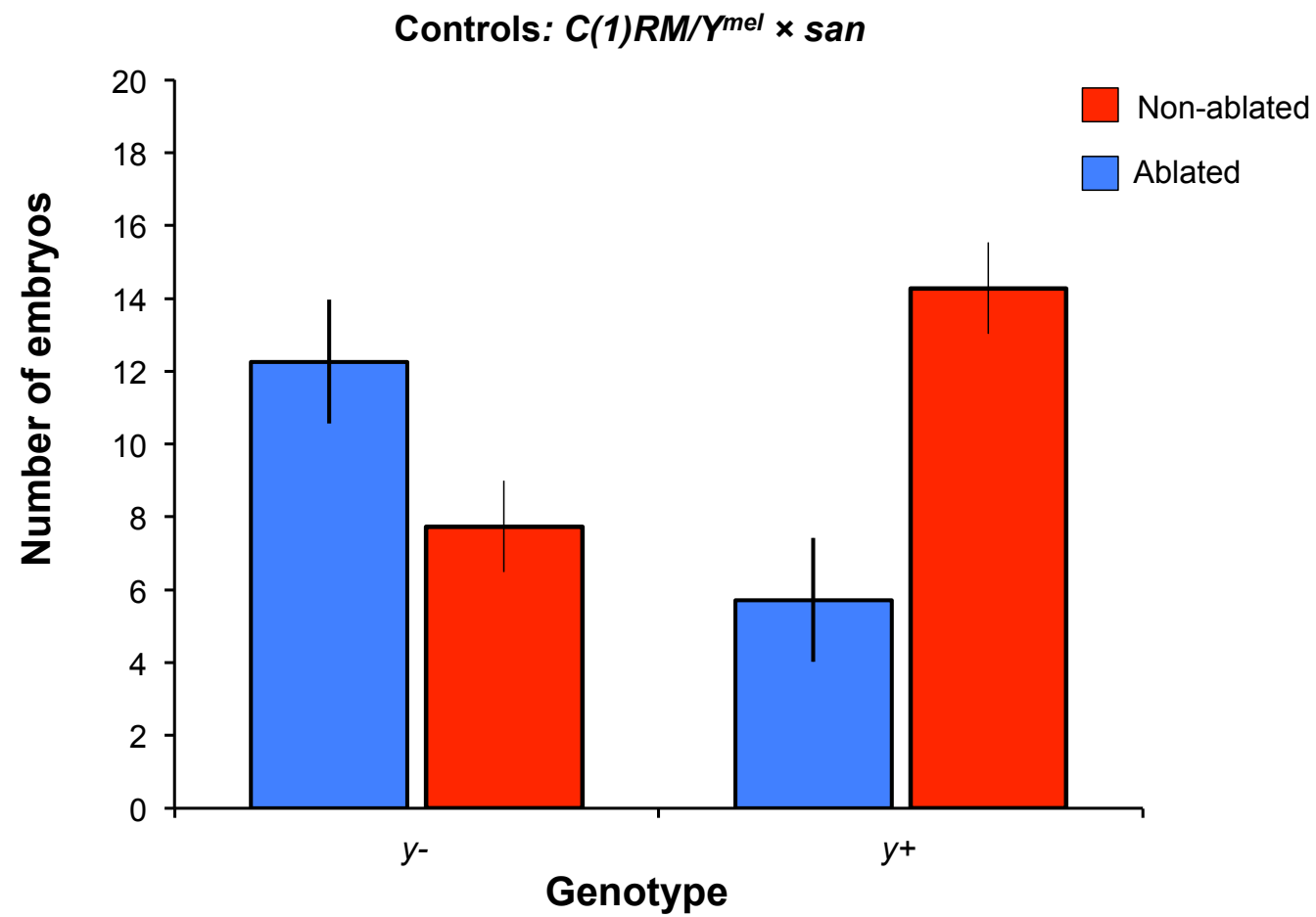

Supplement: Figure S3 — Relative frequency of developmental defects in mel C(1)RM×san crosses. The mel/san hybrid males from the mel C(1)RM×san cross carry a X san chromosome and the vast majority of them are viable. Females from this cross carry two fused Xmel chromosomes (homocompound chromosome), do not survive embryogenesis and show abdominal defects similar to those observed in mel/san hybrid males that carry a Xmel chromosome [53]. (PDF) [file pgen.1004270.s003.pdf]

Number of embryos

Non-ablated  
Ablated

**A. X:2C1-3E4**

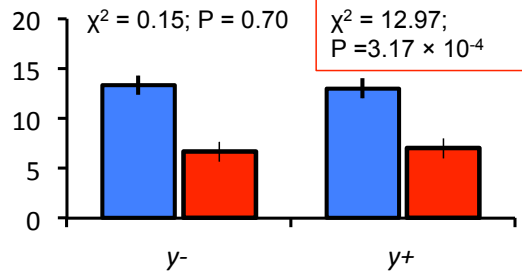

**B. X:4A5-4D7**

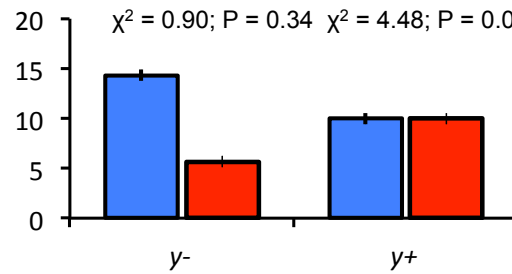

**C. X:5E1-6C7**

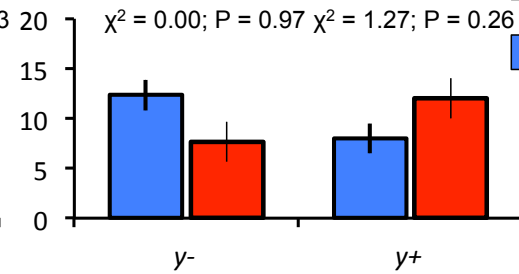

**D. X:7B2-7D18**

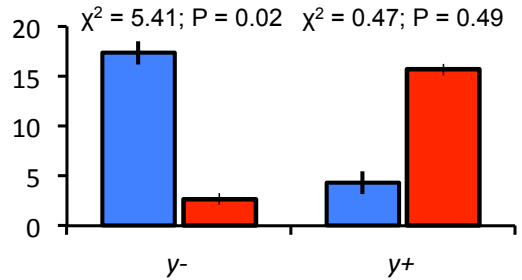

**E. X:11C2-11D1**

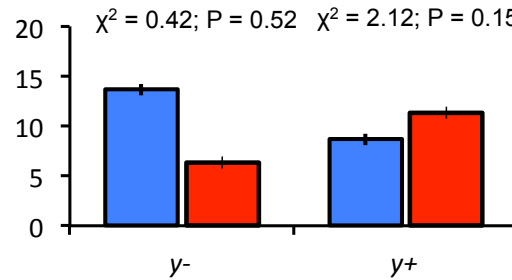

**F. X:11D5--11E8**

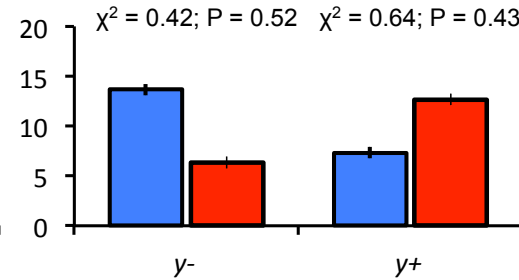

**G. X:12C1-12F4**

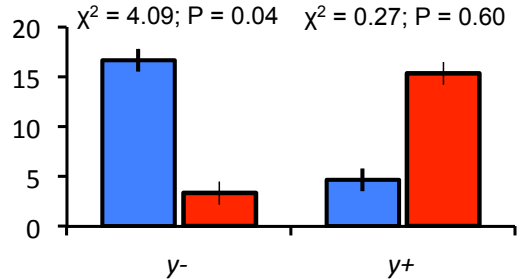

**H. X:12E9-13C5**

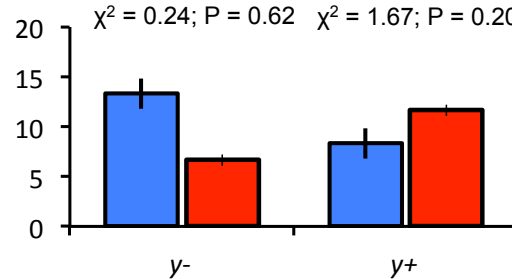

**I. X:16F6-18A7**

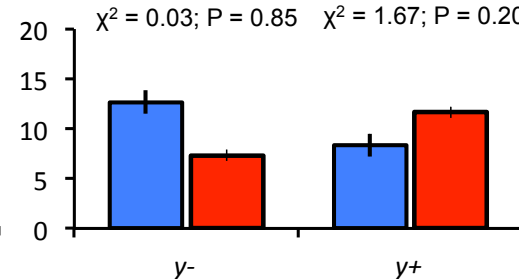

Genotype

Supplement: Figure S4 — Developmental defects show different frequencies in the nine different lethal mel/san hybrid male genotypes. Each barplot corresponds to one of the nine regions (i.e., Dp(1;Y) duplications) that causes hybrid inviability in hybrid males and shows the relative frequency of abdominal ablations in hybrid individuals from both sexes. We measured three replicates for each Dp(1;Y) genotype (each replicate consisted of 20 y+ and 20 y− cuticles). y− are dead female individuals, while y+ can be either hybrid males or hybrid metafemales. We assessed whether the Dp(1;Y) cause a deviation from the expected frequency of cuticular defects by comparing the average number of individuals that show abdominal ablations in each cross in each category (y− or y+) of the cross with the average frequency observed in mel C(1)RM×san crosses (controls, χ2 test, df = 1, Figure S3). We corrected for 18 comparisons comparisons using a Sidak's adjustment, (Significant P<2.8×10^−3^). The only significant P-value is highlighted with a red box. The cytological location of the chromosomal duplication is shown in the top of each histogram. A. Dp(1;Y)BSC75 (X:2C1-3E4). B. Dp(1;Y)BSC159 (X:4A5-4D7). C. Dp(1;Y)BSC289 (X:5E1-6C7). D. Dp(1;Y)BSC176 (X:7B2-7D18). E. Dp(1;Y)BSC126 (X:11C2-11D1). F. Dp(1;Y)BSC327 (X:11D5-11E8). G. Dp(1;Y)BSC186 (X:12C1-12F4). H. Dp(1;Y)BSC269 (X:12E9-13C5). I. Dp(1;Y)BSC11 (X:16F6-18A7). (PDF) [file pgen.1004270.s004.pdf]

Number of incompatibilites

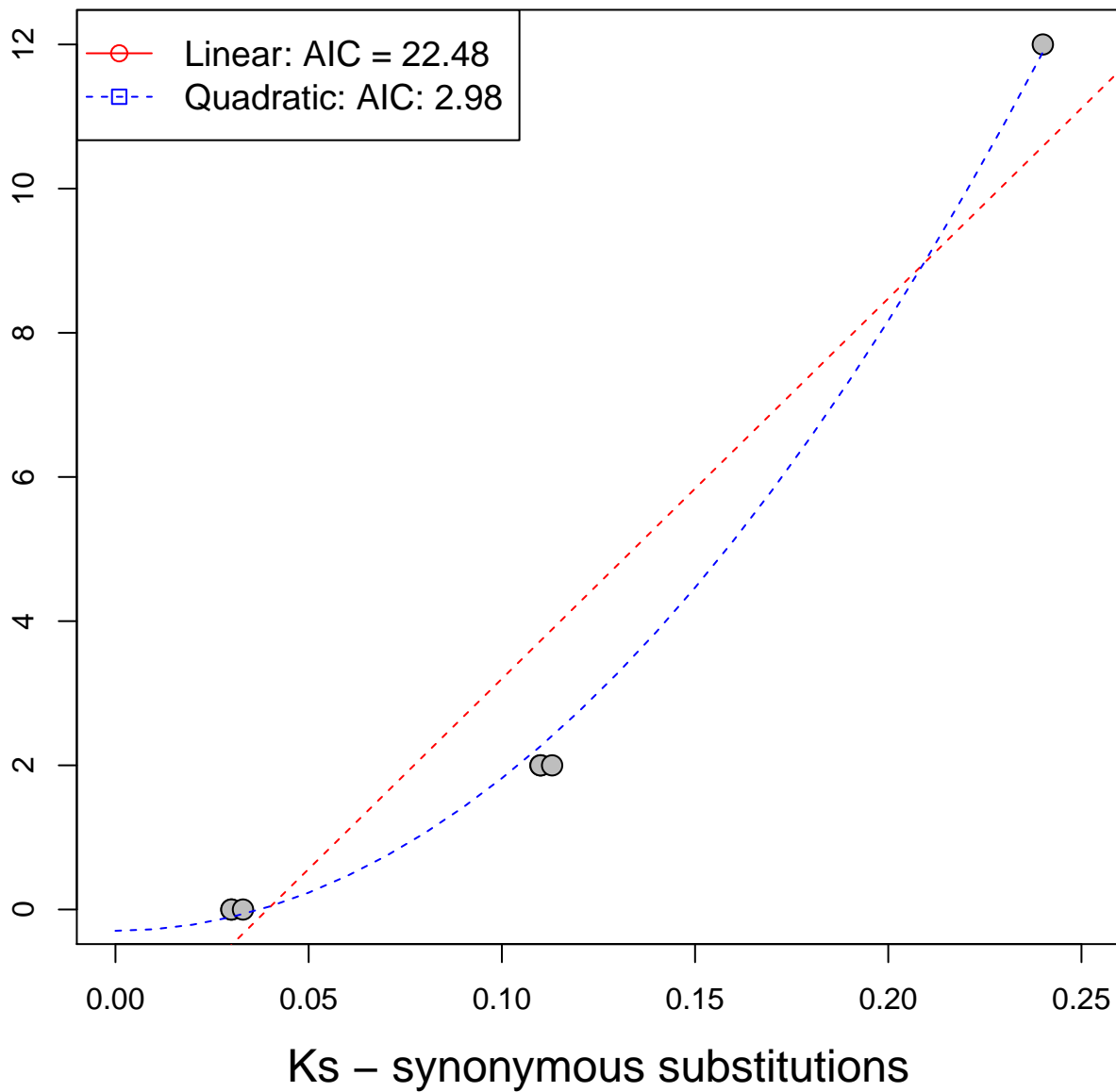

Supplement: Figure S5 — The number of Xmel-linked alleles causing hybrid inviability is higher in the most divergent cross and follows the expectations of the snowball effect theory of hybrid incompatibilities. We used Ks (number of synonymous substitutions per site) as a proxy of divergence time and fitted the best linear (red) and the best quadratic model (blue) to the data. Overlapping points were jittered for clarity. We find the quadratic model has a much better fit than the linear model as evidenced by its lower AIC value. (PDF) [file pgen.1004270.s005.pdf]

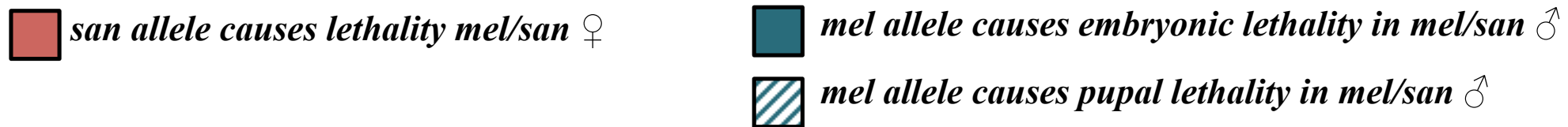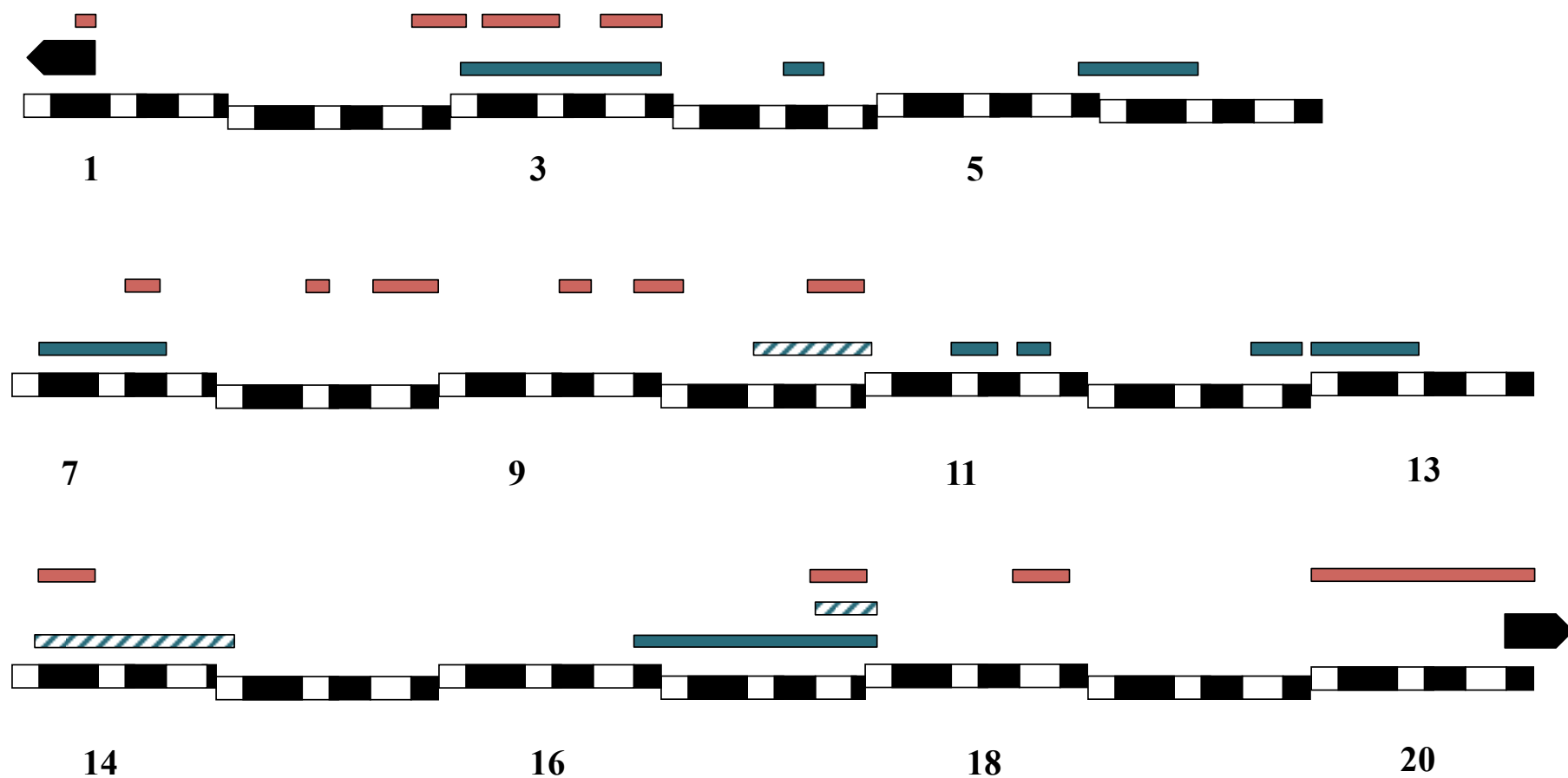

SUPPLEMENTARY FIGURE 5

Supplement: Figure S6 — Comparison of the deficiency mapping results from Matute et al. [22] with our results using duplication mapping. The developmental stage at which the Xsan-linked recessive alleles cause inviability has yet not been determined. (PDF) [file pgen.1004270.s006.pdf]

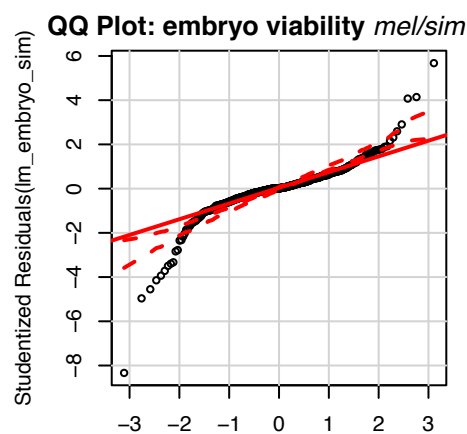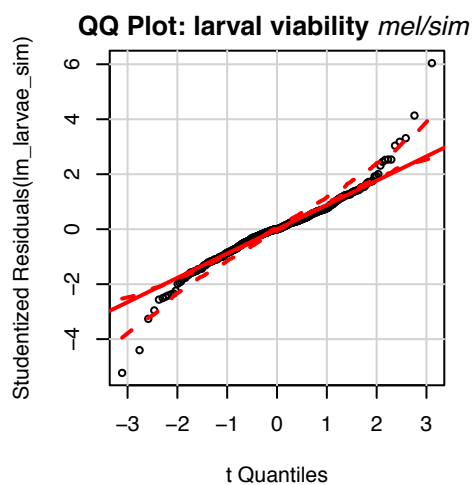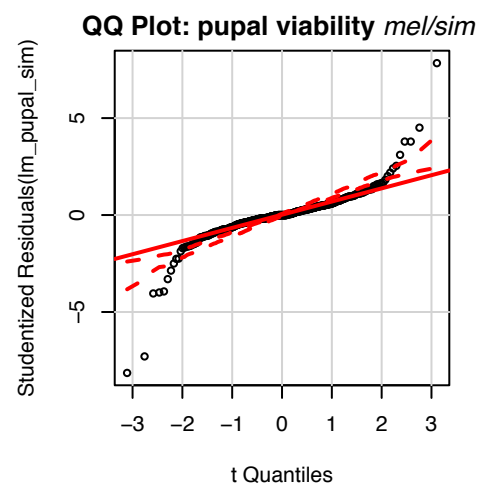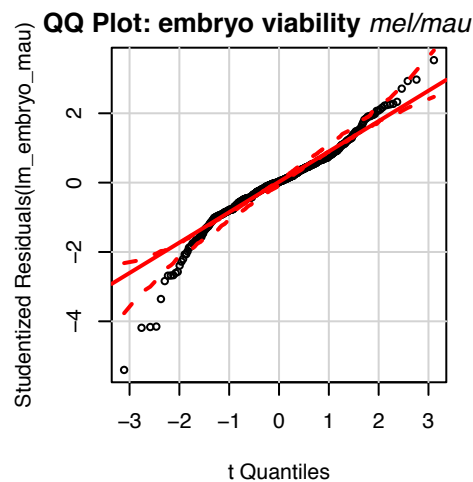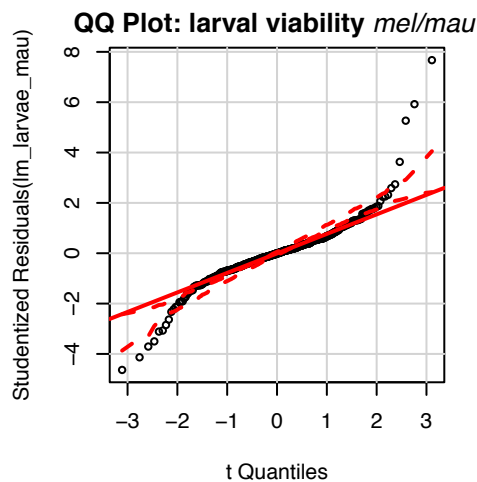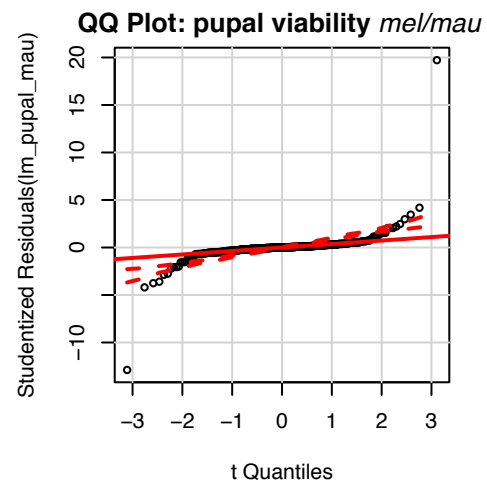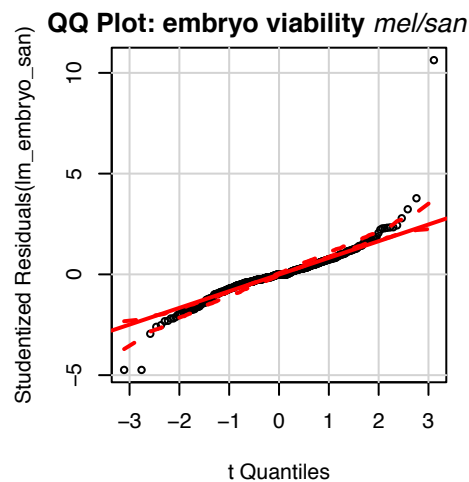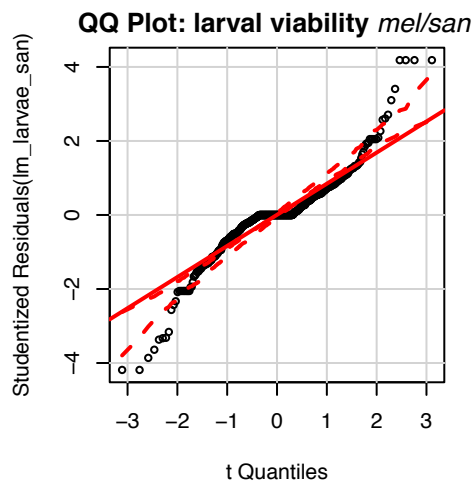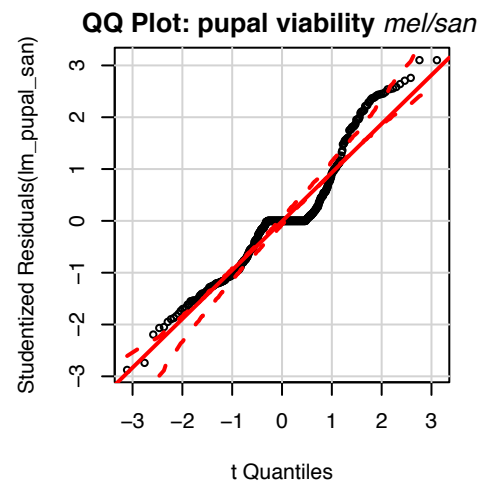

Supplement: Figure S7 — Quatile-quantile plots of the residuals of each of the nine attempted linear models (one for each developmental stage per interspecific cross: 3×3 = 9). Observed values (x-axis) are compared to the values that would be predicted in a normal distribution (y-axis). Dashed red lines give a point-wise 95% confidence interval around the fitted solid red line. Since all the cases showed strong deviations from normality, which in turn precluded the possibility of using linear models, we used a qualitative cut-off to detect lethal alleles. (PDF) [file pgen.1004270.s007.pdf]
