# Supplementary material for: Fine Mapping of Dominant X-Linked Incompatibility Alleles in Drosophila Hybrids
Source: PLoS Genet. 2014 Apr 17;10(4):e1004270. doi: 10.1371/journal.pgen.1004270 (PMC3990725; doi:10.1371/journal.pgen.1004270)
Supplement: Table S1 — Viability and longevity of hybrid males in three interspecific crosses involving D. melanogaster using two different attached-X genetic backgrounds. (DOCX) [file pgen.1004270.s008.docx]

**TABLE S1.**

|  | **Genotype** | ***X/0*** | | | | ***X/Y^mel^*** | | | |
| --- | --- | --- | --- | --- | --- | --- | --- | --- | --- |
|  |  | **Viability** | | **Longevity** | | **Viability** | | **Longevity** | |
|  |  | **Mean (days)** | **Standard deviation** | **Mean (days)** | **Standard deviation** | **Mean (days)** | **Standard deviation** | **Mean (days)** | **Standard deviation** |
| ***C(1)RM*** | ***mel/san*** | 0.876 | 0.080 | 39.683 | 22.100 | 0.852 | 0.082 | 39.683 | 22.100 |
|  | ***mel/sim*** | 0.873 | 0.071 | 39.192 | 20.944 | 0.876 | 0.082 | 36.192 | 20.944 |
|  | ***mel/mau*** | 0.864 | 0.087 | 33.325 | 19.322 | 0.879 | 0.091 | 33.325 | 19.322 |
| ***C(1)DX*** | ***mel/san*** | 0.861 | 0.096 | 36.233 | 18.987 | 0.870 | 0.092 | 38.975 | 22.303 |
|  | ***mel/sim*** | 0.846 | 0.110 | 41.267 | 18.814 | 0.859 | 0.070 | 37.567 | 22.065 |
|  | ***mel/mau*** | 0.844 | 0.083 | 38.750 | 21.647 | 0.902 | 0.088 | 36.642 | 19.582 |
