# Supplementary material for: Fine Mapping of Dominant X-Linked Incompatibility Alleles in Drosophila Hybrids
Source: PLoS Genet. 2014 Apr 17;10(4):e1004270. doi: 10.1371/journal.pgen.1004270 (PMC3990725; doi:10.1371/journal.pgen.1004270)
Supplement: Table S2 — Neither the presence of a Ymel or the D. melanogaster genetic background of the attached-X stock significantly affected hybrid male viability or hybrid male longevity. Viability was analyzed with a full-factorial linear model, while longevity was analyzed with a linear mixed model (vial as a random effect, See Methods). Since there were six linear models, P-values were adjusted with a Sidak's multiple comparison correction; required P for significance <8.512×10^−3^). (DOCX) [file pgen.1004270.s009.docx]

**TABLE S2.**

|  |  | ***Y^mel^*** | | **Background** | | ***Y^mel^* × background** | |
| --- | --- | --- | --- | --- | --- | --- | --- |
|  |  | **F_1,75_** | **P** | **F_1,75_** | **P** | **F_1,75_** | **P** |
| **Viability** | ***mel/san*** | 0.084 | 0.773 | 0.000 | 0.9911 | 0.869 | 0.354 |
|  | ***mel/sim*** | 0.261 | 0.611 | 1.661 | 0.201 | 0.143 | 0.707 |
|  | ***mel/mau*** | 3.314 | 0.073 | 0.010 | 0.919 | 1.159 | 0.285 |
|  |  | **F_1,467_** | **P** | **F_1,467_** | **P** | **F_1,467_** | **P** |
| **Longevity** | ***mel/san*** | 0.502 | 0.479 | 1.155 | 0.283 | 0.502 | 0.479 |
|  | ***mel/sim*** | 0.965 | 0.326 | 2.933 | 0.087 | 0.965 | 0.326 |
|  | ***mel/mau*** | 0.334 | 0.564 | 5.736 | 0.017 | 0.334 | 0.564 |
