# Supplementary material for: Fine Mapping of Dominant X-Linked Incompatibility Alleles in Drosophila Hybrids
Source: PLoS Genet. 2014 Apr 17;10(4):e1004270. doi: 10.1371/journal.pgen.1004270 (PMC3990725; doi:10.1371/journal.pgen.1004270)
Supplement: Table S3 — Mutant lines used for this study. The details of the D. santomea, D. simulans and D. mauritiana are listed in the text. List of all the stocks (other than the Y-linked X duplication stocks) used in this study. (DOCX) [file pgen.1004270.s010.docx]

**TABLE S3.**

| **Genotype** | **Stock Number** |
| --- | --- |
| *dor^4^/C(1)RM, y^1^ w^1^ f^1^* | 35 |
| *C(1)DX, y^1^ w^1^ f^1^/winscy, P{hs-hid}^5^* | 24643 |
| *w*; P{Sxl-Pe-EGFP.G}^G78b^* | 24105 |
| *C(1)RM/C(1;Y)^6^, y^1^ w^1^ f^1^/0* | 9460 |
