# Supplementary material for: Fine Mapping of Dominant X-Linked Incompatibility Alleles in Drosophila Hybrids
Source: PLoS Genet. 2014 Apr 17;10(4):e1004270. doi: 10.1371/journal.pgen.1004270 (PMC3990725; doi:10.1371/journal.pgen.1004270)
Supplement: Table S4 — Duplication stocks used in this study. The table lists panel of Y-linked X duplication chromosome stocks obtained from the Bloomington Stock Center, stock number and genotype for all crosses attempted. Only 52% of the attempted crosses produced progeny in five attempts (shown in Table S6). (DOCX) [file pgen.1004270.s011.docx]

**TABLE S4.**

| **Stock Number** | **Name** | **Cytology** |
| --- | --- | --- |
| **33866** | Dp(1;Y)BSC302 | [1Lt; 1B5] [1D2-1D4; 2B17] [20F3-h28; h28-h29] |
| **29799** | Dp(1;Y)BSC75 | [1Lt; 1B5] [2C1-2C8; 3E4] [20F3-h28; h28-h29] |
| **29801** | Dp(1;Y)BSC77 | [1Lt; 1B5] [3A2-3A6; 3E4] [20F3-h28; h28-h29] |
| **29802** | Dp(1;Y)BSC78 | [1Lt; 1B5] [3A2-3A6; 3E4] [20F3-h28; h28-h29] |
| **29803** | Dp(1;Y)BSC79 | [1Lt; 1B5] [3A2-3A6; 3E4] [20F3-h28; h28-h29] |
| **29807** | Dp(1;Y)BSC83 | [1Lt; 1B5] [3B3-3B4; 3E4] [20F3-h28; h28-h29] |
| **29808** | Dp(1;Y)BSC84 | [1Lt; 1B5] [3C2-3C3; 3E4] [20F3-h28; h28-h29] |
| **29809** | Dp(1;Y)BSC85 | [1Lt; 1B5] [3C2-3C3; 3E4] [20A3-20C1; h28-h29] |
| **29811** | Dp(1;Y)BSC87 | [1Lt; 1B5] [3C3-3C6; 3E4] [20F3-h28; h28-h29] |
| **29812** | Dp(1;Y)BSC88 | [1Lt; 1B5] [3C6-3D2; 3E4] [20F3-h28; h28-h29] |
| **29814** | Dp(1;Y)BSC90 | [1Lt; 1B5] [3D5-3E4; 3E4 [19E7-19F2; h28-h29] |
| **30568** | Dp(1;Y)BSC159 | [1Lt; 1B5] [4A5-4B1; 4D7] [20F3-h28; h28-h29] |
| **30570** | Dp(1;Y)BSC161 | [1Lt; 1B5] [4B5-4C3; 4D7] [20F3-h28; h28-h29] |
| **30571** | Dp(1;Y)BSC162 | [1Lt; 1B5] [4C3-4C8; 4D7] [20F3-h28; h28-h29] |
| **30574** | Dp(1;Y)BSC165 | [1Lt; 1B5] [4C12-4D2; 4D7] [20F3-h28; h28-h29] |
| **30577** | Dp(1;Y)BSC168 | [1Lt; 1B5] [4C12-4D2; 4D7] [20F3-h28; h28-h29] |
| **30576** | Dp(1;Y)BSC167 | [1Lt; 1B5] [4C12-4D2; 4D7] [20F3-h28; h28-h29] |
| **30571** | Dp(1;Y)BSC162 | [1Lt; 1B5] [4C3-4C8; 4D7] [20F3-h28; h28-h29] |
| **29815** | Dp(1;Y)BSC91 | [1Lt; 1B5] [4D1-4D1; 5D1] |
| **29816** | Dp(1;Y)BSC92 | [1Lt; 1B5] [4D6-4D7; 5D1] |
| **29817** | Dp(1;Y)BSC93 | [1Lt; 1B5] [4E2-4E2; 5D1] |
| **29818** | Dp(1;Y)BSC94 | [1Lt; 1B5] [4E2-4F1; 5D1] |
| **29819** | Dp(1;Y)BSC95 | [1Lt; 1B5] [4F4-4F4; 5D1] |
| **29820** | Dp(1;Y)BSC96 | [1Lt; 1B5] [4F9-4F10; 5D1] |
| **33841** | Dp(1;Y)BSC277 | [1Lt; 1B5] [4F10-5A8; 6C7] [20F3-h28; h28-h29] |
| **33845** | Dp(1;Y)BSC281 | [1Lt; 1B5] [5A8-5A12; 6C7] [20F3-h28; h28-h29] |
| **29821** | Dp(1;Y)BSC97 | [1Lt; 1B5] [5A8-5A9; 5D1] |
| **33845** | Dp(1;Y)BSC281 | [1Lt; 1B5] [5A8-5A12; 6C7] [20F3-h28; h28-h29] |
| **33844** | Dp(1;Y)BSC280 | [1Lt; 1B5] [5A8-5A12; 6C7] [20F3-h28; h28-h29] |
| **29822** | Dp(1;Y)BSC98 | [1Lt; 1B5] [5A10-5A11; 5D1] |
| **33846** | Dp(1;Y)BSC282 | [1Lt; 1B5] [5B1-5B6; 6C7] [20F3-h28; h28-h29] |
| **33848** | Dp(1;Y)BSC284 | [1Lt; 1B5] [5C2-5C4; 6C7] [20F3-h28; h28-h29] |
| **33849** | Dp(1;Y)BSC285 | [1Lt; 1B5] [5C4-5C7; 6C7] [20F3-h28; h28-h29] |
| **29823** | Dp(1;Y)BSC99 | [1Lt; 1B5] [5C6-5C6; 5D1] |
| **33853** | Dp(1;Y)BSC289 | [1Lt; 1B5] [5E1-5E4; 6C7] [20F3-h28; h28-h29] |
| **33854** | Dp(1;Y)BSC290 | [1Lt; 1B5] [5E4-5E6; 6C7] [20F3-h28; h28-h29] |
| **33856** | Dp(1;Y)BSC292 | [1Lt; 1B5] [5F4-6B1; 6C7] [20F3-h28; h28-h29] |
| **32128** | Dp(1;Y)BSC172 | [1Lt; 1B5] [7A3-7B1; 7D18] [20F3-h28; h28-h29] |
| **32132** | Dp(1;Y)BSC176 | [1Lt; 1B5] [7B2-7B6; 7D18] [20F3-h28; h28-h29] |
| **32130** | Dp(1;Y)BSC174 | [1Lt; 1B5] [7B2-7B6; 7D18] [20F3-h28; h28-h29] |
| **32136** | Dp(1;Y)BSC180 | [1Lt; 1B5] [7D5-7D6; 7D18] [20F3-h28; h28-h29] |
| **29757** | Dp(1;Y)BSC33 | [1Lt; 1B5] [7D12-7D18; 8C3] [20F3-h28; h28-h29] |
| **32140** | Dp(1;Y)BSC184 | [1Lt; 1B5] [7D16-7D17; 7D18] |
| **29758** | Dp(1;Y)BSC34 | [1Lt; 1B5] [7D18-7E1; 8C3] [20F3-h28; h28-h29] |
| **29759** | Dp(1;Y)BSC35 | [1Lt; 1B5] [7D18-7E1; 8C3] [20F3-h28; h28-h29] |
| **29760** | Dp(1;Y)BSC36 | [1Lt; 1B5] [7E1-7E6; 8C3] [20F3-h28; h28-h29] |
| **29761** | Dp(1;Y)BSC37 | [1Lt; 1B5] [7E1-7E6; 8C3] [20F3-h28; h28-h29] |
| **29762** | Dp(1;Y)BSC38 | [1Lt; 1B5] [7E11-7F2; 8C3] [20F3-h28; h28-h29] |
| **29763** | Dp(1;Y)BSC39 | [1Lt; 1B5] [7F2-7F7; 8C3] [20A3-20C1; h28-h29] |
| **29764** | Dp(1;Y)BSC40 | [1Lt; 1B5] [7F7-8A2; 8C3] |
| **32117** | Dp(1;Y)BSC170 | [1Lt; 1B5] [7F7-8A2; 8F9] [20F3-h28; h28-h29] |
| **29765** | Dp(1;Y)BSC41 | [1Lt; 1B5] [8A2-8A2; 8C3] [20F3-h28; h28-h29] |
| **29767** | Dp(1;Y)BSC43 | [1Lt; 1B5] [8A2-8A2; 8C3] [20A3-20C1; h28-h29] |
| **30520** | Dp(1;Y)BSC144 | [1Lt; 1B5] [8A2-8A2; 8F9] [20F3-h28; h28-h29] |
| **30522** | Dp(1;Y)BSC146 | [1Lt; 1B5] [8A2-8B6; 8F9] [20F3-h28; h28-h29] |
| **32118** | Dp(1;Y)BSC171 | [1Lt; 1B5] [8A2-8A2; 8F9] [20F3-h28; h28-h29] |
| **30531** | Dp(1;Y)BSC155 | [1Lt; 1B5] [8E4-8E12; 8F9] [20F3-h28; h28-h29] |
| **29770** | Dp(1;Y)BSC46 | [1Lt; 1B5] [8B6-8C3; 8C3] [20F3-h28; h28-h29] |
| **29782** | Dp(1;Y)BSC58 | [1Lt; 1B5] [8D9-8E4; 9E2] |
| **29783** | Dp(1;Y)BSC59 | [1Lt; 1B5] [9B1-9B4; 9E2] [20F3-h28; h28-h29] |
| **29784** | Dp(1;Y)BSC60 | [1Lt; 1B5] [9B7-9B14; 9E2] [20A3-20C1; h28-h29] |
| **29785** | Dp(1;Y)BSC61 | [1Lt; 1B5] [9B7-9B14; 9E2] [20F3-h28; h28-h29] |
| **29788** | Dp(1;Y)BSC64 | [1Lt; 1B5] [9D4-9E1; 9E2] [19F2--19F4--h28--h29 |
| **29790** | Dp(1;Y)BSC66 | [1Lt; 1B5] [9D4-9E1; 9E2] [20F3-h28; h28-h29] |
| **33029** | Dp(1;Y)BSC220 | [1Lt; 1B5] [9A4-9B1; 10B14] [20F3-h28; h28-h29] |
| **33031** | Dp(1;Y)BSC221 | [1Lt; 1B5] [9C4-9D4; 10B14] [20F3-h28; h28-h29] |
| **29771** | Dp(1;Y)BSC47 | [1Lt; 1B5] [10B3-10B3; 11A1] [19F4-20A1; h28-h29] |
| **29773** | Dp(1;Y)BSC49 | [1Lt; 1B5] [10B10-10B13; 11A1] [20F3-h28; h28-h29] |
| **29824** | Dp(1;Y)BSC100 | [1Lt; 1B5] [10B14-10C5; 11D1] [20A3-20C1; h28-h29] |
| **29825** | Dp(1;Y)BSC101 | [1Lt; 1B5] [10C5-10C7; 11D1] [20F3-h28; h28-h29] |
| **29775** | Dp(1;Y)BSC51 | [1Lt; 1B5] [10C5-10C7; 11A1] [20F3-h28; h28-h29] |
| **29776** | Dp(1;Y)BSC52 | [1Lt; 1B5] [10C5-10C7; 11A1] [20A3-20C1; h28-h29] |
| **29826** | Dp(1;Y)BSC102 | [1Lt; 1B5] [10C7-10D5; 11D1] [20F3-h28; h28-h29] |
| **29827** | Dp(1;Y)BSC103 | [1Lt; 1B5] [10C7-10D5; 11D1] |
| **29778** | Dp(1;Y)BSC54 | [1Lt; 1B5] [10C7-10D5; 11A1] [20A3-20C1; h28--h29] |
| **29779** | Dp(1;Y)BSC55 | [1Lt; 1B5] [10D5-10E2; 11A1] [20F3-h28; h28-h29] |
| **29828** | Dp(1;Y)BSC104 | [1Lt; 1B5] [10F3-10F7; 11D1] [20F3-h28; h28-h29] |
| **29829** | Dp(1;Y)BSC105 | [1Lt; 1B5] [10F3-10F7; 11D1] [20A3-20C1; h28-h29] |
| **29781** | Dp(1;Y)BSC57 | [1Lt; 1B5] [11A1-11A1; 11A1] [20F3-h28; h28-h29] |
| **29831** | Dp(1;Y)BSC107 | [1Lt; 1B5] [11A4-11A9; 11D1] [20F3-h28; h28-h29] |
| **29832** | Dp(1;Y)BSC108 | [1Lt; 1B5] [11A4-11A9; 11D1] [20F3-h28; h28-h29] |
| **29833** | Dp(1;Y)BSC109 | [1Lt; 1B5] [11A4-11A9; 11D1] [20F3-h28; h28-h29] |
| **29834** | Dp(1;Y)BSC110 | [1Lt; 1B5] [11A4-11A9; 11D1] [20F3-h28; h28-h29] |
| **29835** | Dp(1;Y)BSC111 | [1Lt; 1B5] [11A4-11A9; 11D1] [20F3-h28; h28-h29] |
| **29836** | Dp(1;Y)BSC112 | [1Lt; 1B5] [11A4-\-11A9; 11D1] [20F3-h28; h28-h29] |
| **36380** | Dp(1;Y)BSC322 | [1Lt; 1B5] [11A9-11A11; 11E8] [20F3-h28; h28-h29] |
| **36376** | Dp(1;Y)BSC318 | [1Lt; 1B5] [11A4-11A9; 11E8] [20F3-h28; h28-h29] |
| **29837** | Dp(1;Y)BSC113 | [1Lt; 1B5] [11A9-11A11; 11D1] [20F3-h28; h28-h29] |
| **29838** | Dp(1;Y)BSC114 | [1Lt; 1B5] [11A9-11A11; 11D1] [20F3-h28; h28-h29] |
| **29841** | Dp(1;Y)BSC117 | [1Lt; 1B5] [11A9-11A11; 11D1] [20F3-h28; h28-h29] |
| **29842** | Dp(1;Y)BSC118 | [1Lt; 1B5] [11A9-11A11; 11D1] [20A3-20C1; h28-h29] |
| **29843** | Dp(1;Y)BSC119 | [1Lt; 1B5] [11A11-11B1; 11D1] |
| **29844** | Dp(1;Y)BSC120 | [1Lt; 1B5] [11B1-11B7; 11D1] [19F4-20A1; h28-h29] |
| **29845** | Dp(1;Y)BSC121 | [1Lt; 1B5] [11B1-11B7; 11D1] [20F3-h28; h28-h29] |
| **29846** | Dp(1;Y)BSC122 | [1Lt; 1B5] [11B1-11B7; 11D1] [20F3-h28; h28-h29] |
| **29847** | Dp(1;Y)BSC123 | [1Lt; 1B5] [11B7-11B14; 11D1] [20F3-h28; h28-h29] |
| **29848** | Dp(1;Y)BSC124 | [1Lt; 1B5] [11B7-11B14; 11D1] [20F3-h28; h28-h29] |
| **29849** | Dp(1;Y)BSC125 | [1Lt; 1B5] [11C2-11D1; 11D1] |
| **29850** | Dp(1;Y)BSC126 | [1Lt; 1B5] [11C2-11D1; 11D1] [20F3-h28; h28-h29] |
| **29851** | Dp(1;Y)BSC127 | [1Lt; 1B5] [11C2-11D1; 11D1] [20F3-h28; h28-h29] |
| **29852** | Dp(1;Y)BSC128 | [1Lt; 1B5] [11C2-11D1; 11D1] [20F3-h28; h28-h29] |
| **36385** | Dp(1;Y)BSC327 | [1Lt; 1B5] [11D5-11D10; 11E8] [20F3-h28; h28-h29] |
| **32141** | Dp(1;Y)BSC185 | [1Lt; 1B5] [12A4-12A9; 12F4] [20F3-h28; h28-h29] |
| **32142** | Dp(1;Y)BSC186 | [1Lt; 1B5] [12C1-12C6; 12F4] [20F3-h28; h28-h29] |
| **32143** | Dp(1;Y)BSC187 | [1Lt; 1B5] [12C6-12E2; 12F4] [20F3-h28; h28-h29] |
| **32135** | Dp(1;Y)BSC179 | [1Lt; 1B5] [12C6-12E2; 12F4] [20A3-20C1; h28-h29] |
| **32147** | Dp(1;Y)BSC191 | [1Lt; 1B5] [12E2-12E3; 12F4] [20A3-20C1; h28-h29] |
| **32149** | Dp(1;Y)BSC193 | [1Lt; 1B5] [12E7-12E9; 12F4] [20F3-h28; h28-h29] |
| **33252** | Dp(1;Y)BSC269 | [1Lt; 1B5] [12E9-12F2; 13C5] [20F3-h28; h28-h29] |
| **32154** | Dp(1;Y)BSC198 | [1Lt; 1B5] [12F2-12F4; 12F4] [20F3-h28; h28-h29] |
| **33256** | Dp(1;Y)BSC273 | [1Lt; 1B5] [13A1-13A9; 13C5]] [20F3-h28; h28-h29] |
| **33243** | Dp(1;Y)BSC266 | [1Lt; 1B5] [13C5-13D3; 14A9] [20F3-h28; h28-h29] |
| **33245** | Dp(1;Y)BSC224 | [1Lt; 1B5] [13C5-13D3; 14A9] [20F3-h28; h28-h29] |
| **32529** | Dp(1;Y)BSC240 | [1Lt; 1B5] [14A1-14A5; 15A8] [20A3-20C1; h28-h29] |
| **32156** | Dp(1;Y)BSC200 | [1Lt; 1B5] [14E1-14F2; 16C1] [20A3-20C1; h28-h29] |
| **32158** | Dp(1;Y)BSC202 | [1Lt; 1B5] [15A11-15C4; 16C1] |
| **32167** | Dp(1;Y)BSC211 | [1Lt; 1B5] [16B7-16C1; 16C1] [20A3-20C1; h28-h29] |
| **32530** | Dp(1;Y)BSC241 | [1Lt; 1B5] [14A9-14B9; 15A8] [20F3-h28; h28-h29] |
| **32532** | Dp(1;Y)BSC243 | [1Lt; 1B5] [14C1-14C4; 15A8] [20F3-h28; h28-h29] |
| **32533** | Dp(1;Y)BSC244 | [1Lt; 1B5] [14C4-14E1; 15A8] [20F3-h28; h28-h29] |
| **32538** | Dp(1;Y)BSC249 | [1Lt; 1B5] [14F5-15A1; 15A8] [20F3-h28; h28-h29] |
| **29791** | Dp(1;Y)BSC67 | [1Lt; 1B5] [15F4-15F9; 17C1] [20F3-h28; h28-h29] |
| **29792** | Dp(1;Y)BSC68 | [1Lt; 1B5] [16C1-16E1; 17C1] [20F3-h28; h28-h29] |
| **32528** | Dp(1;Y)BSC239 | [1Lt; 1B5] [14F2-14F5; 14F5] [20F3-h28; h28-h29] |
| **29736** | Dp(1;Y)BSC11 | [1Lt; 1B5] [16F6-17A1; 18A7] [20F3-h28; h28-h29] |
| **29737** | Dp(1;Y)BSC12 | [1Lt; 1B5] [16F6-17A1; 18A7] |
| **29738** | Dp(1;Y)BSC13 | [1Lt; 1B5] [17A2-17A8; 18A7] [20F3-h28; h28-h29] |
| **29739** | Dp(1;Y)BSC14 | [1Lt; 1B5] [17A2-17A8; 18A7] [20F3-h28; h28-h29] |
| **29741** | Dp(1;Y)BSC16 | [1Lt; 1B5] [17C1-17C6; 18A7] [20F3-h28; h28-h29] |
| **29743** | Dp(1;Y)BSC18 | [1Lt; 1B5] [17C6-17D4; 18A7] [20F3-h28; h28-h29] |
| **29744** | Dp(1;Y)BSC19 | [1Lt; 1B5] [17C6-17D4; 18A7] [20F3-h28; h28-h29] |
| **29745** | Dp(1;Y)BSC20 | [1Lt; 1B5] [17D4-17E1; 18A7] [20F3-h28; h28-h29] |
| **29747** | Dp(1;Y)BSC22 | [1Lt; 1B5] [17E1-17F3; 18A7] [20A3-20C1; h28-h29] |
| **29748** | Dp(1;Y)BSC23 | [1Lt; 1B5] [17E1-17F3; 18A7] [20F3-h28; h28-h29] |
| **29749** | Dp(1;Y)BSC24 | [1Lt; 1B5] [17E1-17F3; 18A7] [20F3-h28; h28-h29] |
| **29752** | Dp(1;Y)BSC27 | [1Lt; 1B5] [17F3-18A3; 18A7] [20F3-h28; h28-h29] |
| **29754** | Dp(1;Y)BSC29 | [1Lt; 1B5] [17F3-18A3; 18A7] [20A3-20C1; h28-h29] |
| **29755** | Dp(1;Y)BSC30 | [1Lt; 1B5] [18A3-18A6; 18A7 [20A3-20C1; h28-h29] |
| **29756** | Dp(1;Y)BSC31 | [1Lt; 1B5] [18A3-18A6; 18A7] [19E2-19E5; h28-h29] |
| **29794** | Dp(1;Y)BSC70 | [1Lt; 1B5] [17A1-17A2; 17C1] [20F3-h28; h28-h29] |
| **29795** | Dp(1;Y)BSC71 | [1Lt; 1B5] [17A1-17A2; 17C1] [20F3-h28; h28-h29] |
| **29797** | Dp(1;Y)BSC73 | [1Lt; 1B5] [17A8-17B3; 17C1] [20F3-h28; h28-h29] |
| **30450** | Dp(1;Y)BSC129 | [1Lt; 1B5] [17C1-17C6; 19A2] [20F3-h28; h28-h29] |
| **30451** | Dp(1;Y)BSC130 | [17F3; 19A2] [20F3-h28; h28-h29] |
| **30452** | Dp(1;Y)BSC131 | [1Lt; 1B5] [17F3-18A3; 19A2] [20F3-h28; h28-h29] |
| **30453** | Dp(1;Y)BSC132 | [1Lt; 1B5] [17F3-18A3; 19A2] [20F3-h28; h28-h29] |
| **30454** | Dp(1;Y)BSC133 | [1Lt; 1B5] [18A3-18A6; 19A2] [20F3-h28; h28-h29] |
| **30455** | Dp(1;Y)BSC134 | [1Lt; 1B5] [18B6-18C2; 19A2] [20F3-h28; h28-h29] |
| **30456** | Dp(1;Y)BSC135 | [1Lt; 1B5] [18B6-18C2; 19A2] [20F3-h28; h28-h29] |
| **30457** | Dp(1;Y)BSC136 | [1Lt; 1B5] [18C2-18C7; 19A2] [20C1-20C3; h28-h29] |
| **30458** | Dp(1;Y)BSC137 | [1Lt; 1B5] [18D7-18D13; 19A2] [20F3-h28; h28-h29] |
| **30459** | Dp(1;Y)BSC138 | [1Lt; 1B5] [18D7-18D13; 19A2] [20F3-h28; h28-h29] |
| **30460** | Dp(1;Y)BSC139 | [1Lt; 1B5] [18D13-18E3; 19A2] [20F3-h28; h28-h29] |
| **30461** | Dp(1;Y)BSC140 | [1Lt; 1B5] [18E3-18F1; 19A2] [20A3-20C1; h28-h29] |
| **30462** | Dp(1;Y)BSC141 | [1Lt; 1B5] [18F4-19A2; 19A2] [20F3-h28; h28-h29] |
| **30463** | Dp(1;Y)BSC142 | [1Lt; 1B5] [18F4-19A2; 19A2] [20F3-h28; h28-h29] |
| **30464** | Dp(1;Y)BSC143 | [1Lt; 1B5] [18F4-19A2; 19A2] [20A3-20C1; h28-h29] |
| **36471** | Dp(1;Y)BSC330 | [1Lt; 1B5] [18F4-19B1; 20C3] [20F3-h28; h28-h29] |
