# Supplementary material for: Fine Mapping of Dominant X-Linked Incompatibility Alleles in Drosophila Hybrids
Source: PLoS Genet. 2014 Apr 17;10(4):e1004270. doi: 10.1371/journal.pgen.1004270 (PMC3990725; doi:10.1371/journal.pgen.1004270)
Supplement: Table S5 — Insemination rates in all interspecific and intraspecific crosses. Insemination rates were measured by dissecting groups of 20 females and observing whether females had sperm in their reproductive tract (3 replicates per genotype). The mean number of mated females and the standard error are shown for each cross. (DOCX) [file pgen.1004270.s012.docx]

**TABLE S5.**

| **Stock Number** | **Mean *D. simulans*** | **SD**  ***D. simulans*** | **Mean *D. mauritiana*** | **SD**  ***D. mauritiana*** | **Mean**  ***D. santomea*** | **SD**  ***D. santomea*** | **Mean *mel* Malawi-6-3** | **SD *mel* Malawi-6-3** | **Mean *mel* Malawi-9-2** | **SD *mel* Malawi-9-2** |
| --- | --- | --- | --- | --- | --- | --- | --- | --- | --- | --- |
| **33866** | 0.0500 | 0.0500 | 0.1667 | 0.0289 | 0.0167 | 0.0289 | 1.000 | 0.000 | 1 | 0.000 |
| **29799** | 0.1167 | 0.0289 | 0.1167 | 0.0764 | 0.0333 | 0.0577 | 1.000 | 0.000 | 1 | 0.000 |
| **29801** | 0.1167 | 0.0764 | 0.1500 | 0.0500 | 0.0167 | 0.0289 | 1.000 | 0.000 | 1 | 0.000 |
| **29802** | 0.1500 | 0.0866 | 0.1167 | 0.1258 | 0.0000 | 0.0000 | 1.000 | 0.000 | 1 | 0.000 |
| **29803** | 0.1333 | 0.0289 | 0.2000 | 0.0500 | 0.0333 | 0.0289 | 1.000 | 0.000 | 1 | 0.000 |
| **29804** | 0.1667 | 0.0764 | 0.1167 | 0.0289 | 0.0333 | 0.0577 | 1.000 | 0.000 | 1 | 0.000 |
| **29806** | 0.1333 | 0.1041 | 0.1000 | 0.1000 | 0.0500 | 0.0500 | 1.000 | 0.000 | 1 | 0.000 |
| **29808** | 0.1000 | 0.0866 | 0.1000 | 0.0000 | 0.0167 | 0.0289 | 1.000 | 0.000 | 1 | 0.000 |
| **29811** | 0.1333 | 0.0289 | 0.1000 | 0.0500 | 0.0000 | 0.0000 | 1.000 | 0.000 | 1 | 0.000 |
| **30568** | 0.1167 | 0.0764 | 0.1167 | 0.0289 | 0.0000 | 0.0000 | 1.000 | 0.000 | 1 | 0.000 |
| **30570** | 0.1500 | 0.0500 | 0.1167 | 0.0764 | 0.0333 | 0.0289 | 1.000 | 0.000 | 1 | 0.000 |
| **30571** | 0.1500 | 0.0500 | 0.1167 | 0.0764 | 0.0333 | 0.0577 | 1.000 | 0.000 | 1 | 0.000 |
| **30576** | 0.1000 | 0.0500 | 0.1833 | 0.0764 | 0.0167 | 0.0289 | 1.000 | 0.000 | 1 | 0.000 |
| **30571** | 0.2167 | 0.0289 | 0.1333 | 0.0577 | 0.0500 | 0.0866 | 1.000 | 0.000 | 1 | 0.000 |
| **29815** | 0.2000 | 0.0500 | 0.1833 | 0.0577 | 0.0333 | 0.0577 | 1.000 | 0.000 | 1 | 0.000 |
| **29816** | 0.0833 | 0.0289 | 0.2167 | 0.0289 | 0.0167 | 0.0289 | 1.000 | 0.000 | 1 | 0.000 |
| **29817** | 0.1333 | 0.1155 | 0.1500 | 0.0500 | 0.0167 | 0.0289 | 1.000 | 0.000 | 1 | 0.000 |
| **29818** | 0.1333 | 0.0577 | 0.0833 | 0.0577 | 0.0167 | 0.0289 | 1.000 | 0.000 | 1 | 0.000 |
| **29820** | 0.1833 | 0.0764 | 0.1500 | 0.0500 | 0.0000 | 0.0000 | 1.000 | 0.000 | 1 | 0.000 |
| **33845** | 0.1500 | 0.0866 | 0.1667 | 0.0289 | 0.0500 | 0.0500 | 1.000 | 0.000 | 1 | 0.000 |
| **33844** | 0.1667 | 0.0289 | 0.1333 | 0.0577 | 0.0333 | 0.0289 | 1.000 | 0.000 | 1 | 0.000 |
| **29822** | 0.1667 | 0.0577 | 0.0833 | 0.0289 | 0.0333 | 0.0577 | 1.000 | 0.000 | 1 | 0.000 |
| **33846** | 0.1167 | 0.0764 | 0.1000 | 0.0500 | 0.0333 | 0.0577 | 1.000 | 0.000 | 1 | 0.000 |
| **33848** | 0.0833 | 0.0764 | 0.1167 | 0.0289 | 0.1000 | 0.0866 | 1.000 | 0.000 | 1 | 0.000 |
| **33849** | 0.1500 | 0.0500 | 0.1000 | 0.0500 | 0.0333 | 0.0577 | 1.000 | 0.000 | 1 | 0.000 |
| **29823** | 0.1500 | 0.1323 | 0.1000 | 0.0500 | 0.0000 | 0.0000 | 1.000 | 0.000 | 1 | 0.000 |
| **33853** | 0.1167 | 0.0289 | 0.0833 | 0.0289 | 0.0500 | 0.0866 | 1.000 | 0.000 | 1 | 0.000 |
| **33854** | 0.1500 | 0.0500 | 0.0833 | 0.0289 | 0.0167 | 0.0289 | 1.000 | 0.000 | 1 | 0.000 |
| **33856** | 0.1000 | 0.0500 | 0.1167 | 0.1041 | 0.0500 | 0.0866 | 1.000 | 0.000 | 1 | 0.000 |
| **32128** | 0.0667 | 0.0764 | 0.1000 | 0.0500 | 0.0333 | 0.0577 | 1.000 | 0.000 | 1 | 0.000 |
| **32132** | 0.1333 | 0.0577 | 0.1667 | 0.1041 | 0.0500 | 0.0000 | 1.000 | 0.000 | 1 | 0.000 |
| **32130** | 0.0667 | 0.0764 | 0.0833 | 0.0289 | 0.0333 | 0.0577 | 1.000 | 0.000 | 1 | 0.000 |
| **32136** | 0.0500 | 0.0866 | 0.1000 | 0.1000 | 0.0167 | 0.0289 | 1.000 | 0.000 | 1 | 0.000 |
| **29757** | 0.1167 | 0.0289 | 0.1167 | 0.1041 | 0.0000 | 0.0000 | 1.000 | 0.000 | 1 | 0.000 |
| **29758** | 0.1500 | 0.0500 | 0.1333 | 0.1041 | 0.0333 | 0.0577 | 1.000 | 0.000 | 1 | 0.000 |
| **29759** | 0.1333 | 0.1155 | 0.0833 | 0.0289 | 0.0167 | 0.0289 | 1.000 | 0.000 | 1 | 0.000 |
| **29760** | 0.1500 | 0.0500 | 0.1167 | 0.0577 | 0.0000 | 0.0000 | 1.000 | 0.000 | 1 | 0.000 |
| **29761** | 0.1333 | 0.1041 | 0.0833 | 0.0289 | 0.0500 | 0.0500 | 1.000 | 0.000 | 1 | 0.000 |
| **29764** | 0.0833 | 0.0577 | 0.1167 | 0.1041 | 0.0000 | 0.0000 | 1.000 | 0.000 | 1 | 0.000 |
| **29765** | 0.0500 | 0.0866 | 0.1500 | 0.0500 | 0.0333 | 0.0577 | 1.000 | 0.000 | 1 | 0.000 |
| **30520** | 0.1333 | 0.0289 | 0.0833 | 0.0289 | 0.0000 | 0.0000 | 1.000 | 0.000 | 1 | 0.000 |
| **30522** | 0.1333 | 0.0764 | 0.0833 | 0.1443 | 0.0000 | 0.0000 | 1.000 | 0.000 | 1 | 0.000 |
| **30531** | 0.1500 | 0.0500 | 0.1500 | 0.0866 | 0.0500 | 0.0866 | 1.000 | 0.000 | 1 | 0.000 |
| **29782** | 0.1167 | 0.0289 | 0.1333 | 0.0764 | 0.0333 | 0.0577 | 1.000 | 0.000 | 1 | 0.000 |
| **29785** | 0.0833 | 0.0764 | 0.0667 | 0.0577 | 0.0333 | 0.0577 | 1.000 | 0.000 | 1 | 0.000 |
| **29788** | 0.1333 | 0.0577 | 0.1000 | 0.0866 | 0.0000 | 0.0000 | 1.000 | 0.000 | 1 | 0.000 |
| **29790** | 0.1500 | 0.0500 | 0.1000 | 0.0500 | 0.0000 | 0.0000 | 1.000 | 0.000 | 1 | 0.000 |
| **33029** | 0.1000 | 0.0500 | 0.1500 | 0.0500 | 0.0500 | 0.0500 | 1.000 | 0.000 | 1 | 0.000 |
| **33031** | 0.1333 | 0.0289 | 0.1000 | 0.0000 | 0.0167 | 0.0289 | 1.000 | 0.000 | 1 | 0.000 |
| **29773** | 0.1500 | 0.0500 | 0.0667 | 0.0764 | 0.0000 | 0.0000 | 1.000 | 0.000 | 1 | 0.000 |
| **29775** | 0.1833 | 0.0764 | 0.1333 | 0.0289 | 0.0333 | 0.0577 | 1.000 | 0.000 | 1 | 0.000 |
| **29776** | 0.1167 | 0.0577 | 0.0667 | 0.0764 | 0.0333 | 0.0577 | 1.000 | 0.000 | 1 | 0.000 |
| **29826** | 0.0833 | 0.0764 | 0.1167 | 0.1258 | 0.0000 | 0.0000 | 1.000 | 0.000 | 1 | 0.000 |
| **29778** | 0.1500 | 0.1323 | 0.1000 | 0.0500 | 0.0833 | 0.1041 | 1.000 | 0.000 | 1 | 0.000 |
| **29779** | 0.1500 | 0.0500 | 0.1000 | 0.0500 | 0.0333 | 0.0289 | 1.000 | 0.000 | 1 | 0.000 |
| **29828** | 0.1333 | 0.0764 | 0.1333 | 0.0577 | 0.0000 | 0.0000 | 1.000 | 0.000 | 1 | 0.000 |
| **29829** | 0.1167 | 0.0289 | 0.1000 | 0.0866 | 0.0000 | 0.0000 | 1.000 | 0.000 | 1 | 0.000 |
| **29832** | 0.1667 | 0.0764 | 0.1667 | 0.0764 | 0.0000 | 0.0000 | 1.000 | 0.000 | 1 | 0.000 |
| **29836** | 0.1333 | 0.0764 | 0.1500 | 0.0500 | 0.0000 | 0.0000 | 1.000 | 0.000 | 1 | 0.000 |
| **36376** | 0.2000 | 0.0500 | 0.1667 | 0.1041 | 0.0000 | 0.0000 | 1.000 | 0.000 | 1 | 0.000 |
| **29837** | 0.1500 | 0.0500 | 0.1500 | 0.0866 | 0.0500 | 0.0866 | 1.000 | 0.000 | 1 | 0.000 |
| **29841** | 0.1500 | 0.0500 | 0.1500 | 0.1000 | 0.0000 | 0.0000 | 1.000 | 0.000 | 1 | 0.000 |
| **29844** | 0.1500 | 0.0500 | 0.1000 | 0.0500 | 0.0000 | 0.0000 | 1.000 | 0.000 | 1 | 0.000 |
| **29845** | 0.1500 | 0.0500 | 0.1667 | 0.1041 | 0.0000 | 0.0000 | 1.000 | 0.000 | 1 | 0.000 |
| **29848** | 0.1500 | 0.0500 | 0.1667 | 0.0764 | 0.0000 | 0.0000 | 1.000 | 0.000 | 1 | 0.000 |
| **29850** | 0.1167 | 0.0289 | 0.1500 | 0.0500 | 0.0167 | 0.0289 | 1.000 | 0.000 | 1 | 0.000 |
| **29851** | 0.1667 | 0.0764 | 0.1333 | 0.0764 | 0.0000 | 0.0000 | 1.000 | 0.000 | 1 | 0.000 |
| **29852** | 0.1500 | 0.0866 | 0.1167 | 0.1258 | 0.0500 | 0.0866 | 1.000 | 0.000 | 1 | 0.000 |
| **36385** | 0.1333 | 0.0764 | 0.1167 | 0.1258 | 0.0333 | 0.0577 | 1.000 | 0.000 | 1 | 0.000 |
| **32143** | 0.1333 | 0.0289 | 0.2000 | 0.1500 | 0.0333 | 0.0577 | 1.000 | 0.000 | 1 | 0.000 |
| **32135** | 0.1000 | 0.0500 | 0.1667 | 0.1041 | 0.0000 | 0.0000 | 1.000 | 0.000 | 1 | 0.000 |
| **32147** | 0.1333 | 0.0577 | 0.1333 | 0.0764 | 0.0500 | 0.0500 | 1.000 | 0.000 | 1 | 0.000 |
| **32149** | 0.1667 | 0.0764 | 0.1000 | 0.0500 | 0.0167 | 0.0289 | 1.000 | 0.000 | 1 | 0.000 |
| **33252** | 0.1333 | 0.0289 | 0.1500 | 0.1323 | 0.0167 | 0.0289 | 1.000 | 0.000 | 1 | 0.000 |
| **33256** | 0.1333 | 0.0764 | 0.1000 | 0.0000 | 0.0333 | 0.0577 | 1.000 | 0.000 | 1 | 0.000 |
| **33243** | 0.1333 | 0.0764 | 0.1000 | 0.1000 | 0.0167 | 0.0289 | 1.000 | 0.000 | 1 | 0.000 |
| **32529** | 0.1333 | 0.0764 | 0.1833 | 0.0764 | 0.0500 | 0.0866 | 1.000 | 0.000 | 1 | 0.000 |
| **32167** | 0.1000 | 0.0500 | 0.1333 | 0.0577 | 0.0500 | 0.0500 | 1.000 | 0.000 | 1 | 0.000 |
| **32530** | 0.1667 | 0.1041 | 0.1333 | 0.0764 | 0.0000 | 0.0000 | 1.000 | 0.000 | 1 | 0.000 |
| **32533** | 0.1000 | 0.0500 | 0.1833 | 0.0289 | 0.0833 | 0.0764 | 1.000 | 0.000 | 1 | 0.000 |
| **32538** | 0.1167 | 0.0764 | 0.1000 | 0.0500 | 0.0667 | 0.0577 | 1.000 | 0.000 | 1 | 0.000 |
| **29791** | 0.1500 | 0.1000 | 0.1500 | 0.0500 | 0.0000 | 0.0000 | 1.000 | 0.000 | 1 | 0.000 |
| **29792** | 0.1000 | 0.0500 | 0.1667 | 0.0764 | 0.0000 | 0.0000 | 1.000 | 0.000 | 1 | 0.000 |
| **29736** | 0.1500 | 0.0500 | 0.1167 | 0.0289 | 0.1500 | 0.0500 | 1.000 | 0.000 | 1 | 0.000 |
| **29737** | 0.1500 | 0.0500 | 0.1000 | 0.0500 | 0.1500 | 0.0866 | 1.000 | 0.000 | 1 | 0.000 |
| **29741** | 0.1667 | 0.0289 | 0.1167 | 0.0289 | 0.0667 | 0.0289 | 1.000 | 0.000 | 1 | 0.000 |
| **29743** | 0.1167 | 0.0764 | 0.1333 | 0.0577 | 0.0833 | 0.1041 | 1.000 | 0.000 | 1 | 0.000 |
| **29745** | 0.1667 | 0.0289 | 0.1333 | 0.0577 | 0.0667 | 0.0764 | 1.000 | 0.000 | 1 | 0.000 |
| **29747** | 0.1500 | 0.0500 | 0.1167 | 0.1258 | 0.0167 | 0.0289 | 1.000 | 0.000 | 1 | 0.000 |
| **29749** | 0.1000 | 0.0500 | 0.1500 | 0.0866 | 0.0167 | 0.0289 | 1.000 | 0.000 | 1 | 0.000 |
| **29752** | 0.1333 | 0.0764 | 0.1000 | 0.1323 | 0.0333 | 0.0289 | 1.000 | 0.000 | 1 | 0.000 |
| **29754** | 0.1000 | 0.0500 | 0.1000 | 0.1323 | 0.0333 | 0.0577 | 1.000 | 0.000 | 1 | 0.000 |
| **29756** | 0.1500 | 0.0500 | 0.1167 | 0.1155 | 0.0000 | 0.0000 | 1.000 | 0.000 | 1 | 0.000 |
| **29794** | 0.1000 | 0.0500 | 0.1000 | 0.0866 | 0.0000 | 0.0000 | 1.000 | 0.000 | 1 | 0.000 |
| **29795** | 0.1167 | 0.0289 | 0.0833 | 0.0577 | 0.0500 | 0.0500 | 1.000 | 0.000 | 1 | 0.000 |
| **29797** | 0.1000 | 0.0500 | 0.1167 | 0.0764 | 0.0667 | 0.0577 | 1.000 | 0.000 | 1 | 0.000 |
| **30451** | 0.1333 | 0.0577 | 0.1167 | 0.0289 | 0.0000 | 0.0000 | 1.000 | 0.000 | 1 | 0.000 |
| **30454** | 0.1333 | 0.0289 | 0.1500 | 0.0866 | 0.0000 | 0.0000 | 1.000 | 0.000 | 1 | 0.000 |
| **30457** | 0.1333 | 0.0577 | 0.0833 | 0.0577 | 0.0000 | 0.0000 | 1.000 | 0.000 | 1 | 0.000 |
| **30459** | 0.1167 | 0.0764 | 0.1667 | 0.1041 | 0.0167 | 0.0289 | 1.000 | 0.000 | 1 | 0.000 |
| **30460** | 0.1333 | 0.0289 | 0.0667 | 0.0289 | 0.0667 | 0.0764 | 1.000 | 0.000 | 1 | 0.000 |
| **30461** | 0.1500 | 0.0500 | 0.1333 | 0.0764 | 0.0667 | 0.0289 | 1.000 | 0.000 | 1 | 0.000 |
| **30462** | 0.1167 | 0.0764 | 0.1000 | 0.0500 | 0.0000 | 0.0000 | 1.000 | 0.000 | 1 | 0.000 |
| **30463** | 0.1333 | 0.0764 | 0.1000 | 0.0500 | 0.0500 | 0.0000 | 1.000 | 0.000 | 1 | 0.000 |
