# Supplementary material for: Fine Mapping of Dominant X-Linked Incompatibility Alleles in Drosophila Hybrids
Source: PLoS Genet. 2014 Apr 17;10(4):e1004270. doi: 10.1371/journal.pgen.1004270 (PMC3990725; doi:10.1371/journal.pgen.1004270)
Supplement: Table S6 — Viability rates for each developmental transition in the five crosses presented in this report (3 interspecific+2 intraspecific) in crosses involving mel C(1)RM. Averages were calculated with 3 replicates per cross. These data were used to generate Figures 3, 6, and 7. None of the C(1)RM, Dp(1;Y)×mel crosses showed decreases in viability at any developmental stage. The decrease in viability at the larval stage is consistent with the inviability of pure-species metafemales at the late larval stage. (DOCX) [file pgen.1004270.s013.docx]

**TABLE S6.**

| **Stock Number** | ***D. santomea*** | | | ***D. simulans*** | | | ***D. mauritiana*** | | | ***D. melanogaster* Malawi 6.3** | | | ***D. melanogaster* Malawi 9.2** | | |
| --- | --- | --- | --- | --- | --- | --- | --- | --- | --- | --- | --- | --- | --- | --- | --- |
|  | Embryo | Larval | Pupal | Embryo | Larval | Pupal | Embryo | Larval | Pupal | Embryo | Larval | Pupal | Embryo | Larval | Pupal |
| **33866** | 0.389 | 0.787 | 0.548 | 0.921 | 0.461 | 0.429 | 0.880 | 0.423 | 0.881 | 0.960 | 0.725 | 0.951 | 0.860 | 0.701 | 0.917 |
| **29799** | 0.019 | **NA** | **NA** | 0.904 | 0.507 | 0.880 | 0.919 | 0.357 | 0.907 | 0.933 | 0.733 | 0.918 | 0.931 | 0.698 | 0.943 |
| **29801** | 0.072 | **NA** | **NA** | 0.865 | 0.404 | 0.852 | 0.915 | 0.451 | 0.850 | 0.957 | 0.767 | 0.942 | 0.902 | 0.803 | 0.917 |
| **29802** | 0.098 | **NA** | **NA** | 0.904 | 0.391 | 0.915 | 0.900 | 0.421 | 0.817 | 0.957 | 0.798 | 0.910 | 0.950 | 0.715 | 0.950 |
| **29803** | 0.000 | **NA** | **NA** | 0.908 | 0.448 | 0.950 | 0.920 | 0.370 | 0.825 | 0.943 | 0.835 | 0.941 | 0.891 | 0.803 | 0.933 |
| **29808** | 0.237 | 0.767 | 0.500 | 0.939 | 0.433 | 0.774 | 0.929 | 0.423 | 0.903 | 0.950 | 0.965 | 0.953 | 0.967 | 0.699 | 0.941 |
| **30568** | 0.090 | 0.540 | 0.683 | 0.911 | 0.375 | 0.090 | 0.883 | 0.386 | 0.063 | 0.993 | 0.668 | 0.905 | 0.95 | 0.705 | 0.931 |
| **30570** | 0.087 | 0.583 | **NA** | 0.769 | 0.428 | 0.075 | 0.944 | 0.426 | 0.035 | 0.983 | 0.709 | 0.903 | 1.000 | 0.747 | 0.928 |
| **30571** | 0.038 | **NA** | **NA** | 0.848 | 0.461 | 0.087 | 0.927 | 0.438 | 0.097 | 0.970 | 0.701 | 0.907 | 0.935 | 0.751 | 0..941 |
| **30576** | 0.055 | 0.622 | 0.694 | 0.861 | 0.348 | 0.042 | 0.942 | 0.420 | 0.083 | 0.980 | 0.728 | 0.871 | 0.980 | 0.705 | 0.922 |
| **29815** | 0.350 | 0.749 | 0.657 | 0.952 | 0.414 | 0.923 | 0.957 | 0.412 | 0.891 | 0.977 | 0.696 | 0.912 | 0.950 | 0.725 | 0.941 |
| **29816** | 0.398 | 0.624 | 0.552 | 0.876 | 0.468 | 0.837 | 0.921 | 0.426 | 0.867 | 0.980 | 0.756 | 0.912 | 0.899 | 0.699 | 0.954 |
| **29817** | 0.379 | 0.866 | 0.542 | 0.854 | 0.498 | 0.815 | 0.927 | 0.441 | 0.816 | 0.983 | 0.732 | 0.924 | 0.980 | 0.701 | 0.895 |
| **29818** | 0.287 | 0.824 | 0.808 | 0.837 | 0.368 | 0.903 | 0.944 | 0.434 | 0.892 | 0.990 | 0.707 | 0.952 | 0.975 | 0.677 | 0.954 |
| **29820** | 0.376 | 0.559 | 0.775 | 0.845 | 0.422 | 0.857 | 0.946 | 0.402 | 0.876 | 0.983 | 0.733 | 0.927 | 0.996 | 0.789 | 0.986 |
| **33845** | 0.383 | 0.678 | 0.255 | 0.853 | 0.413 | 0.844 | 0.944 | 0.421 | 0.880 | 0.983 | 0.729 | 0.888 | 0.960 | 0.744 | 0.944 |
| **33844** | 0.391 | 0.590 | 0.571 | 0.796 | 0.314 | 0.828 | 0.950 | 0.414 | 0.884 | 0.967 | 0.700 | 0.931 | 1.000 | 0.750 | 0.931 |
| **33846** | 0.368 | 0.590 | 0.715 | 0.872 | 0.380 | 0.867 | 0.960 | 0.620 | 0.865 | 0.973 | 0.695 | 0.941 | 0.966 | 0.696 | 0.911 |
| **33848** | 0.409 | 0.802 | 0.706 | 0.832 | 0.432 | 0.883 | 0.930 | 0.416 | 0.875 | 0.973 | 0.685 | 0.945 | 0.980 | 0.702 | 0.954 |
| **33849** | 0.379 | 0.495 | 0.367 | 0.902 | 0.446 | 0.867 | 0.937 | 0.427 | 0.829 | 0.960 | 0.729 | 0.904 | 0.999 | 0.800 | 0.916 |
| **29823** | 0.302 | 0.533 | 0.818 | 0.894 | 0.413 | 0.725 | 0.936 | 0.419 | 0.859 | 0.977 | 0.717 | 0.904 | 1.000 | 0.698 | 0.917 |
| **33853** | 0.075 | **NA** | **NA** | 0.851 | 0.392 | 0.881 | 0.913 | 0.430 | 0.821 | 0.963 | 0.703 | 0.862 | 0.954 | 0.675 | 0.951 |
| **33854** | 0.000 | **NA** | **NA** | 0.778 | 0.444 | 0.864 | 0.959 | 0.407 | 0.812 | 0.967 | 0.686 | 0.924 | 0.920 | 0.734 | 0.932 |
| **33856** | 0.018 | **NA** | **NA** | 0.869 | 0.363 | 0.866 | 0.966 | 0.372 | 0.811 | 0.967 | 0.693 | 0.872 | 0.912 | 0.679 | 0.917 |
| **32128** | 0.383 | 0.295 | 0.367 | 0.883 | 0.313 | 0.967 | 0.962 | 0.387 | 0.869 | 0.970 | 0.694 | 0.876 | 0.954 | 0.856 | 0.940 |
| **32132** | 0.024 | **NA** | **NA** | 0.880 | 0.436 | 0.856 | 0.942 | 0.402 | 0.880 | 0.963 | 0.775 | 0.874 | 0.976 | 0.81 | 0.915 |
| **32130** | 0.000 | **NA** | **NA** | 0.902 | 0.403 | 0.947 | 0.890 | 0.338 | 0.850 | 0.963 | 0.716 | 0.922 | 0.980 | 0.756 | 0.929 |
| **32136** | 0.355 | 0.352 | 0.381 | 0.741 | 0.468 | 0.873 | 0.889 | 0.383 | 0.835 | 0.973 | 0.712 | 0.957 | 0.971 | 0.875 | 0.940 |
| **29758** | 0.334 | 0.794 | 0.723 | 0.936 | 0.396 | 0.912 | 0.915 | 0.396 | 0.796 | 0.970 | 0.732 | 0.934 | 0.990 | 0.801 | 0.923 |
| **29759** | 0.242 | 0.588 | 0.560 | 0.872 | 0.456 | 0.883 | 0.970 | 0.351 | 0.849 | 0.977 | 0.717 | 0.927 | 0.911 | 0.830 | 0.918 |
| **29760** | 0.349 | 0.871 | 0.817 | 0.781 | 0.330 | 0.884 | 0.923 | 0.472 | 0.846 | 0.963 | 0.689 | 0.925 | 0.927 | 0.789 | 0.909 |
| **29761** | 0.423 | 0.833 | 0.687 | 0.857 | 0.287 | 0.847 | 0.882 | 0.422 | 0.877 | 0.970 | 0.718 | 0.915 | 0.951 | 0.711 | 0.951 |
| **29764** | 0.299 | 0.734 | 0.719 | 0.895 | 0.435 | 0.861 | 0.935 | 0.354 | 0.840 | 0.987 | 0.699 | 0.915 | 0.923 | 0.724 | 0.902 |
| **29765** | 0.288 | 0.763 | 0.716 | 0.856 | 0.444 | 0.949 | 0.945 | 0.438 | 0.785 | 0.973 | 0.716 | 0.904 | 0.914 | 0.699 | 0.933 |
| **30531** | 0.348 | 0.900 | 0.747 | 0.905 | 0.477 | 0.815 | 0.931 | 0.451 | 0.873 | 0.963 | 0.699 | 0.887 | 0.999 | 0.710 | 0.956 |
| **29782** | 0.379 | 0.818 | 0.789 | 0.892 | 0.400 | 0.919 | 0.883 | 0.396 | 0.835 | 0.967 | 0.710 | 0.911 | 1.000 | 0.870 | 0.931 |
| **29785** | 0.369 | 0.862 | 0.748 | 0.906 | 0.037 | **NA** | 0.944 | 0.065 | **NA** | 0.987 | 0.703 | 0.876 | 0.888 | 0.760 | 0.904 |
| **33029** | 0.367 | 0.841 | 0.689 | 0.874 | 0.037 | **NA** | 0.941 | 0.031 | **NA** | 0.967 | 0.710 | 0.923 | 0.910 | 0.800 | 0.929 |
| **33031** | 0.381 | 0.791 | 0.880 | 0.865 | 0.067 | **NA** | 0.891 | 0.094 | **NA** | 0.973 | 0.767 | 0.893 | 0.934 | 0.803 | 0.920 |
| **29775** | 0.324 | 0.916 | 0.740 | 0.889 | 0.470 | 0.911 | 0.864 | 0.414 | 0.842 | 0.970 | 0.701 | 0.956 | 0.907 | 0.799 | 0.900 |
| **29776** | 0.336 | 0.655 | 0.000 | 0.946 | 0.477 | 0.853 | 0.896 | 0.547 | 0.810 | 0.967 | 0.714 | 0.874 | 0.998 | 0.815 | 1.000 |
| **29778** | 0.118 | 0.861 | 0.522 | 0.908 | 0.410 | 0.881 | 0.868 | 0.392 | 0.845 | 0.970 | 0.705 | 0.932 | 0.950 | 0.865 | 0.954 |
| **29779** | 0.361 | 0.891 | 0.479 | 0.880 | 0.395 | 0.860 | 0.899 | 0.409 | 0.862 | 0.960 | 0.705 | 0.926 | 0.917 | 0.713 | 0.981 |
| **29828** | 0.238 | 0.731 | 0.753 | 0.940 | 0.446 | 0.900 | 0.921 | 0.406 | 0.846 | 0.973 | 0.709 | 0.918 | 0.999 | 0.700 | 0.973 |
| **29829** | 0.232 | 0.739 | 0.843 | 0.880 | 0.432 | 0.855 | 0.941 | 0.411 | 0.836 | 0.973 | 0.722 | 0.933 | 1.000 | 0.734 | 0.920 |
| **29837** | 0.187 | 0.848 | 0.925 | 0.719 | 0.442 | 0.849 | 0.866 | 0.399 | 0.835 | 0.977 | 0.689 | 0.927 | 1.000 | 0.689 | 0.940 |
| **29841** | 0.241 | 0.852 | 0.902 | 0.949 | 0.391 | 0.915 | 0.885 | 0.467 | 0.896 | 0.967 | 0.770 | 0.919 | 0.987 | 0.713 | 0.938 |
| **29850** | 0.000 | **NA** | **NA** | 0.924 | 0.387 | 0.867 | 0.937 | 0.411 | 0.857 | 0.967 | 0.735 | 0.931 | 1.000 | 0.705 | 0.928 |
| **29851** | 0.015 | **NA** | **NA** | 0.920 | 0.398 | 0.882 | 0.892 | 0.436 | 0.900 | 0.960 | 0.729 | 0.894 | 0.988 | 0.780 | 0.951 |
| **29852** | 0.000 | **NA** | **NA** | 0.882 | 0.386 | 0.878 | 0.939 | 0.391 | 0.839 | 0.977 | 0.706 | 0.890 | 0.971 | 0.743 | 0.941 |
| **36385** | 0.010 | **NA** | **NA** | 0.901 | 0.390 | 0.912 | 0.886 | 0.361 | 0.914 | 0.967 | 0.714 | 0.917 | 0.999 | 0.718 | 1.000 |
| **32142** | 0.065 | **NA** | **NA** | 0.873 | 0.352 | 0.819 | 0.870 | 0.380 | 0.764 | 0.977 | 0.703 | 0.885 | 0.988 | 0.754 | 0.949 |
| **32143** | 0.011 | **NA** | **NA** | 0.932 | 0.377 | 0.883 | 0.894 | 0.412 | 0.876 | 0.963 | 0.713 | 0.908 | 1.000 | 0.724 | 0.905 |
| **32135** | 0.000 | **NA** | **NA** | 0.883 | 0.412 | 0.856 | 0.923 | 0.416 | 0.836 | 0.960 | 0.719 | 0.919 | 0.965 | 0.755 | 0.953 |
| **32147** | 0.000 | **NA** | **NA** | 0.883 | 0.413 | 0.914 | 0.905 | 0.398 | 0.804 | 0.980 | 0.711 | 0.915 | 0.955 | 0.714 | 0.043 |
| **32149** | 0.378 | 0.736 | 0.847 | 0.933 | 0.411 | 0.925 | 0.912 | 0.422 | 0.869 | 0.987 | 0.700 | 0.890 | 1.000 | 0.705 | 0.951 |
| **33252** | 0.030 | **NA** | **NA** | 0.948 | 0.363 | 0.909 | 0.933 | 0.408 | 0.883 | 0.967 | 0.693 | 0.922 | 0.972 | 0.733 | 0.905 |
| **33256** | 0.000 | **NA** | **NA** | 0.895 | 0.403 | 0.936 | 0.891 | 0.426 | 0.898 | 0.977 | 0.717 | 0.938 | 0.999 | 0.739 | 0.874 |
| **33243** | 0.402 | 0.826 | 0.699 | 0.927 | 0.410 | 0.929 | 0.885 | 0.457 | 0.868 | 0.970 | 0.732 | 0.902 | 0.956 | 0.788 | 0.915 |
| **32529** | 0.403 | 0.827 | 0.090 | 0.927 | 0.395 | 0.949 | 0.851 | 0.438 | 0.829 | 0.960 | 0.722 | 0.914 | 0.966 | 0.728 | 0.981 |
| **32156** | 0.309 | 0.721 | 0.744 | 0.878 | 0.413 | 0.901 | 0.845 | 0.490 | 0.836 | 0.967 | 0.731 | 0.944 | 0.991 | 0.734 | 0.900 |
| **32167** | 0.350 | 0.825 | 0.761 | 0.889 | 0.434 | 0.898 | 0.818 | 0.387 | 0.863 | 0.970 | 0.715 | 0.882 | 0.912 | 0.760 | 0.936 |
| **32530** | 0.352 | 0.913 | 0.087 | 0.931 | 0.404 | 0.864 | 0.935 | 0.454 | 0.825 | 0.963 | 0.731 | 0.915 | 1.000 | 0.777 | 0.904 |
| **32533** | 0.375 | 0.533 | 0.489 | 0.894 | 0.424 | 0.890 | 0.912 | 0.427 | 0.874 | 0.963 | 0.706 | 0.926 | 0.982 | 0.704 | 0.913 |
| **32538** | 0.319 | 0.317 | 0.630 | 0.919 | 0.395 | 0.902 | 0.869 | 0.350 | 0.871 | 0.980 | 0.684 | 0.916 | 0.934 | 0.701 | 0.925 |
| **29736** | 0.024 | **NA** | **NA** | 0.935 | 0.389 | 0.919 | 0.932 | 0.360 | 1.075 | 0.970 | 0.715 | 0.923 | 0.974 | 0.734 | 0.939 |
| **29737** | 0.000 | **NA** | **NA** | 0.866 | 0.407 | 0.882 | 0.908 | 0.462 | 0.870 | 0.970 | 0.698 | 0.922 | 0.937 | 0.699 | 0.918 |
| **29745** | 0.314 | 0.714 | 0.833 | 0.942 | 0.392 | 0.924 | 0.895 | 0.392 | 0.860 | 0.967 | 0.762 | 0.926 | 0.918 | 0.714 | 0.921 |
| **29747** | 0.359 | 0.787 | 0.079 | 0.926 | 0.356 | 0.911 | 0.914 | 0.338 | 0.791 | 0.967 | 0.728 | 0.908 | 0.933 | 0.728 | 0.905 |
| **29749** | 0.367 | 0.690 | 0.000 | 0.910 | 0.396 | 0.854 | 0.864 | 0.349 | 0.841 | 0.970 | 0.712 | 0.923 | 0.911 | 0.745 | 0.933 |
| **29752** | 0.333 | 0.617 | 0.583 | 0.902 | 0.396 | 0.836 | 0.916 | 0.363 | 0.843 | 0.973 | 0.737 | 0.902 | 0.945 | 0.733 | 0.924 |
| **29754** | 0.380 | 0.840 | 0.826 | 0.924 | 0.484 | 0.866 | 0.928 | 0.342 | 0.857 | 0.967 | 0.759 | 0.928 | 0.933 | 0.721 | 1.000 |
| **29794** | 0.367 | 0.819 | 0.586 | 0.916 | 0.437 | 0.924 | 0.883 | 0.420 | 0.883 | 0.973 | 0.740 | 0.921 | 0.962 | 0.774 | 0.900 |
| **29795** | 0.351 | 0.666 | 0.572 | 0.918 | 0.400 | 0.873 | 0.844 | 0.361 | 0.722 | 0.963 | 0.748 | 0.929 | 0.966 | 0.802 | 0.914 |
| **29797** | 0.357 | 0.677 | 0.885 | 0.912 | 0.360 | 0.874 | 0.908 | 0.356 | 0.904 | 0.973 | 0.719 | 0.916 | 1.000 | 0.714 | 0.943 |
| **30459** | 0.323 | 0.901 | 0.770 | 0.975 | 0.445 | 0.902 | 0.829 | 0.468 | 0.796 | 0.960 | 0.726 | 0.879 | 0.964 | 0.723 | 0.914 |
| **30460** | 0.386 | 0.740 | 0.844 | 0.940 | 0.444 | 0.964 | 0.921 | 0.411 | 0.818 | 0.970 | 0.694 | 0.926 | 0.970 | 0.763 | 0.913 |
| **30461** | 0.358 | 0.873 | 0.869 | 0.931 | 0.484 | 0.907 | 0.838 | 0.374 | 0.837 | 0.983 | 0.729 | 0.940 | 0.968 | 0.706 | 0.912 |
| **30462** | 0.446 | 0.715 | 0.786 | 0.894 | 0.403 | 0.893 | 0.909 | 0.444 | 0.859 | 0.967 | 0.761 | 0.914 | 0.933 | 0.735 | 0.911 |
| **30463** | 0.261 | 0.674 | 0.771 | 0.893 | 0.423 | 0.930 | 0.895 | 0.409 | 0.828 | 0.963 | 0.768 | 0.879 | 0.999 | 0.752 | 0.897 |
