# Supplementary material for: Fine Mapping of Dominant X-Linked Incompatibility Alleles in Drosophila Hybrids
Source: PLoS Genet. 2014 Apr 17;10(4):e1004270. doi: 10.1371/journal.pgen.1004270 (PMC3990725; doi:10.1371/journal.pgen.1004270)
Supplement: Table S7 — Viability rates for each developmental transition in the three interspecific crosses presented in this report in crosses involving mel C(1)DX. These data, along with the data presented in Table S6, were used to generate Figures 3, 6, and 7. None of the C(1)DX, Dp(1;Y)×mel crosses showed decreases in viability at any developmental stage. The decrease in viability at the larval stage is consistent with the inviability of pure-species metafemales at the late larval stage. (DOCX) [file pgen.1004270.s014.docx]

**TABLE S7.**

| **Stock Number** | ***D. santomea*** | | | | ***D. simulans*** | | | | ***D. mauritiana*** | | | |
| --- | --- | --- | --- | --- | --- | --- | --- | --- | --- | --- | --- | --- |
|  | **Sample size** | **Embryo** | **Larvae** | **Pupae** | **Sample size** | **Embryo** | **Larvae** | **Pupae** | **Sample size** | **Embryo** | **Larvae** | **Pupae** |
| **33866** | **3** | 0.264 | 0.791 | 0.816 | **3** | 0.942 | 0.366 | 0.906 | 3 | 0.958 | 0.454 | 0.844 |
| **29799** | **3** | 0.015 | NA | NA | **3** | 0.917 | 0.359 | 0.904 | 3 | 0.969 | 0.340 | 0.928 |
| **29801** | **3** | 0.000 | NA | NA | **3** | 0.938 | 0.360 | 0.855 | 3 | 0.937 | 0.489 | 0.886 |
| **29802** | **4** | 0.063 | NA | NA | **3** | 0.776 | 0.461 | 0.794 | 3 | 0.986 | 0.403 | 0.867 |
| **29803** | **3** | 0.029 | NA | NA | **3** | 0.967 | 0.460 | 0.820 | 3 | 0.945 | 0.304 | 0.654 |
| **29808** | **3** | 0.366 | 0.689 | 0.681 | **3** | 0.922 | 0.325 | 0.689 | 3 | 0.961 | 0.360 | 0.928 |
| **30568** | **3** | 0.026 | NA | NA | **3** | 0.958 | 0.091 | NA | 3 | 0.966 | 0.078 | NA |
| **30570** | **3** | 0.020 | NA | NA | **3** | 0.939 | 0.093 | NA | 3 | 0.942 | 0.082 | NA |
| **30571** | **5** | 0.017 | NA | NA | **3** | 0.964 | 0.090 | NA | 3 | 0.965 | 0.048 | NA |
| **30576** | **3** | 0.031 | NA | NA | **3** | 0.987 | 0.076 | NA | 3 | 0.815 | 0.067 | NA |
| **29815** | **3** | 0.319 | 0.483 | 0.769 | **3** | 0.931 | 0.335 | 0.879 | 3 | 0.912 | 0.348 | 0.912 |
| **29816** | **4** | 0.230 | 0.592 | 0.628 | **3** | 0.973 | 0.457 | 0.878 | 3 | 0.928 | 0.425 | 0.939 |
| **29817** | **4** | 0.398 | 0.682 | 0.723 | **3** | 0.917 | 0.413 | 0.875 | 3 | 0.953 | 0.391 | 0.910 |
| **29818** | **3** | 0.269 | 0.828 | 0.882 | **3** | 0.931 | 0.401 | 0.847 | 3 | 0.939 | 0.391 | 0.936 |
| **29820** | **3** | 0.342 | 0.569 | 0.527 | **3** | 0.894 | 0.421 | 0.788 | 3 | 0.967 | 0.445 | 0.891 |
| **33845** | **3** | 0.265 | 0.655 | 0.757 | **3** | 0.921 | 0.485 | 0.744 | 3 | 0.968 | 0.494 | 0.765 |
| **33844** | **3** | 0.319 | 0.268 | 0.976 | **3** | 0.868 | 0.344 | 0.695 | 3 | 0.935 | 0.460 | 0.840 |
| **33846** | **3** | 0.250 | 0.668 | 0.853 | **3** | 0.936 | 0.331 | 0.868 | 3 | 0.910 | 0.486 | 0.885 |
| **33848** | **4** | 0.252 | 0.534 | 0.784 | **3** | 0.931 | 0.394 | 0.867 | 3 | 0.940 | 0.419 | 0.849 |
| **33849** | **3** | 0.355 | 0.221 | 0.929 | **3** | 0.942 | 0.433 | 0.933 | 3 | 0.980 | 0.400 | 2.154 |
| **29823** | **3** | 0.353 | 0.471 | 0.781 | **4** | 0.943 | 0.529 | 0.823 | 3 | 0.931 | 0.386 | 0.842 |
| **33853** | **3** | 0.019 | NA | NA | **3** | 0.922 | 0.317 | 0.725 | 3 | 0.947 | 0.440 | 0.912 |
| **33854** | **4** | 0.018 | NA | NA | **3** | 0.938 | 0.320 | 0.679 | 3 | 0.946 | 0.432 | 0.864 |
| **33856** | **3** | 0.039 | NA | NA | **3** | 0.948 | 0.265 | 0.757 | 3 | 0.954 | 0.413 | 0.915 |
| **32128** | **3** | 0.241 | 0.856 | 0.782 | **4** | 0.919 | 0.370 | 0.830 | 3 | 0.956 | 0.384 | 0.877 |
| **32132** | **3** | 0.046 | NA | NA | **3** | 0.957 | 0.341 | 0.711 | 3 | 0.947 | 0.451 | 0.857 |
| **32130** | **3** | 0.022 | NA | NA | **3** | 0.974 | 0.343 | 0.811 | 3 | 0.969 | 0.433 | 0.829 |
| **32136** | **3** | 0.349 | 0.718 | 0.833 | **3** | 0.955 | 0.426 | 0.921 | 3 | 0.960 | 0.489 | 0.783 |
| **29758** | **3** | 0.344 | 0.699 | 0.648 | **3** | 0.943 | 0.267 | 0.846 | 3 | 0.919 | 0.425 | 0.860 |
| **29759** | **3** | 0.227 | 0.527 | 0.762 | **3** | 0.967 | 0.389 | 0.944 | 3 | 0.949 | 0.333 | 0.915 |
| **29760** | **3** | 0.206 | 0.743 | 0.711 | **3** | 0.897 | 0.416 | 0.806 | 3 | 0.932 | 0.440 | 0.767 |
| **29761** | **3** | 0.274 | 0.787 | 0.746 | **3** | 0.935 | 0.395 | 0.863 | 3 | 0.911 | 0.407 | 0.807 |
| **29764** | **3** | 0.271 | 0.599 | 0.577 | **3** | 0.943 | 0.207 | 0.774 | 3 | 0.934 | 0.447 | 0.897 |
| **29765** | **3** | 0.296 | 0.497 | 0.840 | **3** | 0.935 | 0.380 | 0.788 | 3 | 0.937 | 0.482 | 0.875 |
| **30531** | **4** | 0.216 | 0.532 | 0.621 | **3** | 0.939 | 0.362 | 0.945 | 3 | 0.960 | 0.429 | 0.783 |
| **29782** | **4** | 0.283 | 0.539 | 0.456 | **3** | 0.930 | 0.374 | 0.623 | 3 | 0.940 | 0.393 | 0.909 |
| **29785** | **5** | 0.156 | 0.596 | 0.565 | **3** | 0.671 | 0.042 | NA | 3 | 0.945 | 0.031 | NA |
| **33029** | **4** | 0.250 | 0.601 | 0.671 | **3** | 0.910 | 0.031 | NA | 3 | 0.922 | 0.042 | NA |
| **33031** | **3** | 0.283 | 0.779 | 0.712 | **3** | 0.875 | 0.069 | NA | 3 | 0.984 | 0.027 | NA |
| **29775** | **4** | 0.271 | 0.492 | 0.640 | **3** | 0.955 | 0.335 | 0.864 | 3 | 0.920 | 0.540 | 0.749 |
| **29776** | **4** | 0.302 | 0.611 | 0.074 | **3** | 0.990 | 0.189 | 0.513 | 2 | 0.959 | 0.306 | 0.909 |
| **29778** | **3** | 0.309 | 0.750 | 0.765 | **3** | 1.000 | 0.218 | 0.714 | 3 | 0.978 | 0.203 | 0.516 |
| **29779** | **4** | 0.218 | 0.514 | 0.958 | **3** | 0.930 | 0.439 | 0.932 | 3 | 0.946 | 0.363 | 0.791 |
| **29828** | **3** | 0.313 | 0.784 | 0.790 | **4** | 0.889 | 0.395 | 0.843 | 3 | 1.000 | 0.299 | 0.575 |
| **29829** | **4** | 0.246 | 0.517 | 0.941 | **3** | 0.909 | 0.370 | 0.910 | 3 | 0.928 | 0.374 | 0.933 |
| **29837** | **3** | 0.260 | 0.794 | 0.572 | **3** | 0.944 | 0.443 | 0.935 | 3 | 0.952 | 0.450 | 0.861 |
| **29841** | **3** | 0.249 | 0.812 | 0.525 | **3** | 0.923 | 0.370 | 0.868 | 3 | 0.979 | 0.190 | 0.442 |
| **29850** | **4** | 0.042 | NA | NA | **3** | 0.883 | 0.375 | 0.891 | 3 | 0.940 | 0.464 | 0.863 |
| **29851** | **3** | 0.021 | NA | NA | **3** | 0.963 | 0.326 | 0.835 | 3 | 0.914 | 0.429 | 0.889 |
| **29852** | **4** | 0.028 | NA | NA | **3** | 0.943 | 0.400 | 1.000 | 3 | 0.950 | 0.423 | 0.845 |
| **36385** | **4** | 0.078 | NA | NA | **3** | 0.977 | 0.463 | 0.908 | 3 | 0.900 | 0.391 | 0.904 |
| **32142** | **4** | 0.014 | NA | NA | **3** | 0.952 | 0.358 | 0.817 | 3 | 0.932 | 0.397 | 0.926 |
| **32143** | **4** | 0.039 | NA | NA | **3** | 0.912 | 0.383 | 0.908 | 3 | 0.896 | 0.459 | 0.846 |
| **32135** | **4** | 0.085 | NA | NA | **3** | 0.944 | 0.393 | 0.912 | 3 | 0.951 | 0.310 | 0.891 |
| **32147** | **4** | 0.049 | NA | NA | **3** | 0.925 | 0.384 | 0.860 | 3 | 0.960 | 0.433 | 0.940 |
| **32149** | **4** | 0.295 | 0.542 | 0.852 | **3** | 0.896 | 0.452 | 0.866 | 3 | 0.898 | 0.452 | 0.872 |
| **33252** | **3** | 0.000 | NA | NA | **3** | 0.981 | 0.362 | 0.783 | 3 | 0.950 | 0.410 | 0.864 |
| **33256** | **5** | 0.079 | NA | NA | **3** | 0.880 | 0.403 | 0.852 | 3 | 0.939 | 0.441 | 0.818 |
| **33243** | **3** | 0.268 | 0.729 | 0.844 | **3** | 0.959 | 0.287 | 0.917 | 3 | 0.921 | 0.475 | 0.774 |
| **32529** | **4** | 0.342 | 0.673 | 0.061 | **4** | 0.972 | 0.296 | 0.929 | 3 | 0.952 | 0.435 | 0.850 |
| **32156** | **3** | 0.350 | 0.557 | 0.519 | **3** | 0.915 | 0.427 | 0.888 | 3 | 0.937 | 0.436 | 0.979 |
| **32167** | **3** | 0.464 | 0.540 | 0.868 | **3** | 0.928 | 0.370 | 0.841 | 3 | 0.933 | 0.418 | 0.935 |
| **32530** | **3** | 0.442 | 0.725 | 0.084 | **3** | 0.933 | 0.409 | 0.837 | 3 | 0.962 | 0.442 | 0.817 |
| **32533** | **5** | 0.184 | 0.401 | 0.941 | **3** | 0.875 | 0.320 | 0.895 | 3 | 0.963 | 0.426 | 0.872 |
| **32538** | **4** | 0.219 | 0.776 | 0.547 | **3** | 0.936 | 0.462 | 0.820 | 3 | 0.935 | 0.372 | 0.947 |
| **29736** | **5** | 0.096 | NA | NA | **3** | 0.916 | 0.408 | 0.852 | 3 | 0.924 | 0.435 | 0.846 |
| **29737** | **3** | 0.032 | NA | NA | **3** | 0.956 | 0.410 | 0.842 | 3 | 0.939 | 0.410 | 0.874 |
| **29745** | **3** | 0.293 | 0.802 | 0.842 | **4** | 0.882 | 0.402 | 0.912 | 3 | 0.974 | 0.447 | 0.923 |
| **29747** | **4** | 0.306 | 0.849 | 0.089 | **4** | 0.910 | 0.438 | 0.919 | 3 | 0.916 | 0.422 | 0.904 |
| **29749** | **3** | 0.246 | 0.883 | 0.053 | **4** | 0.887 | 0.340 | 0.944 | 3 | 0.876 | 0.402 | 0.842 |
| **29752** | **5** | 0.214 | 0.441 | 0.121 | **3** | 0.894 | 0.389 | 0.797 | 3 | 0.972 | 0.432 | 0.884 |
| **29754** | **3** | 0.295 | 0.775 | 0.618 | **3** | 0.956 | 0.418 | 0.818 | 3 | 0.938 | 0.422 | 0.758 |
| **29794** | **3** | 0.344 | 0.734 | 0.857 | **4** | 0.926 | 0.350 | 0.925 | 3 | 0.957 | 0.455 | 0.838 |
| **29795** | **3** | 0.363 | 0.745 | 0.762 | **4** | 0.918 | 0.380 | 0.874 | 3 | 0.947 | 0.450 | 0.875 |
| **29797** | **3** | 0.326 | 0.866 | 0.714 | **4** | 0.929 | 0.304 | 0.900 | 3 | 0.953 | 0.363 | 0.901 |
| **30459** | **4** | 0.266 | 0.420 | 0.590 | **3** | 0.865 | 0.437 | 0.907 | 3 | 0.944 | 0.414 | 0.869 |
| **30460** | **3** | 0.254 | 0.793 | 0.921 | **3** | 0.948 | 0.525 | 0.829 | 3 | 0.931 | 0.381 | 0.872 |
| **30461** | **3** | 0.325 | 0.858 | 0.713 | **4** | 0.876 | 0.379 | 0.907 | 3 | 0.971 | 0.396 | 0.937 |
| **30462** | **3** | 0.414 | 0.629 | 0.738 | **3** | 0.903 | 0.352 | 1.000 | 3 | 0.924 | 0.402 | 0.885 |
| **30463** | **4** | 0.022 | NA | NA | **3** | 0.964 | 0.315 | 0.883 | 3 | 0.988 | 0.330 | 0.784 |
