# Supplementary material for: Fine Mapping of Dominant X-Linked Incompatibility Alleles in Drosophila Hybrids
Source: PLoS Genet. 2014 Apr 17;10(4):e1004270. doi: 10.1371/journal.pgen.1004270 (PMC3990725; doi:10.1371/journal.pgen.1004270)
Supplement: Table S8 — Male and female progeny counts produced in crosses between mel C(1)RM/Dp(1;Y)×mel Malawi-6-3 and mel Malawi-9-2. No significant deviations from the 1∶1 ratio were observed in any of the assayed Dp(1;Y) duplications. (DOCX) [file pgen.1004270.s015.docx]

**TABLE S8.**

| **Stock Number** | **Females** | **Males** | **Females (Expected)** | **Males (Expected)** | **χ^2^ (df =1)** | **P** |
| --- | --- | --- | --- | --- | --- | --- |
| **33866** | 89 | 77 | 83 | 83 | 0.867 | 0.352 |
| **29799** | 105 | 100 | 102.5 | 102.5 | 0.122 | 0.727 |
| **29801** | 190 | 177 | 183.5 | 183.5 | 0.460 | 0.497 |
| **29802** | 82 | 93 | 87.5 | 87.5 | 0.691 | 0.406 |
| **29803** | 45 | 56 | 50.5 | 50.5 | 1.198 | 0.274 |
| **29808** | 33 | 29 | 31 | 31 | 0.258 | 0.611 |
| **30568** | 150 | 131 | 140.5 | 140.5 | 1.285 | 0.257 |
| **30570** | 128 | 140 | 134 | 134 | 0.537 | 0.464 |
| **30571** | 144 | 129 | 136.5 | 136.5 | 0.824 | 0.364 |
| **30576** | 118 | 143 | 130.5 | 130.5 | 2.395 | 0.122 |
| **29815** | 56 | 70 | 63 | 63 | 1.556 | 0.212 |
| **29816** | 104 | 110 | 107 | 107 | 0.168 | 0.682 |
| **29817** | 113 | 127 | 120 | 120 | 0.817 | 0.366 |
| **29818** | 150 | 134 | 142 | 142 | 0.901 | 0.342 |
| **29820** | 110 | 99 | 104.5 | 104.5 | 0.579 | 0.447 |
| **33845** | 67 | 80 | 73.5 | 73.5 | 1.150 | 0.284 |
| **33844** | 77 | 89 | 83 | 83 | 0.867 | 0.352 |
| **33846** | 56 | 70 | 63 | 63 | 1.556 | 0.212 |
| **33848** | 48 | 59 | 53.5 | 53.5 | 1.131 | 0.288 |
| **33849** | 86 | 91 | 88.5 | 88.5 | 0.141 | 0.707 |
| **29823** | 105 | 120 | 112.5 | 112.5 | 1.000 | 0.317 |
| **33853** | 144 | 171 | 157.5 | 157.5 | 2.314 | 0.128 |
| **33854** | 102 | 88 | 95 | 95 | 1.032 | 0.310 |
| **33856** | 96 | 77 | 86.5 | 86.5 | 2.087 | 0.149 |
| **32128** | 104 | 120 | 112 | 112 | 1.143 | 0.285 |
| **32132** | 67 | 80 | 73.5 | 73.5 | 1.150 | 0.284 |
| **32130** | 44 | 52 | 48 | 48 | 0.667 | 0.414 |
| **32136** | 23 | 34 | 28.5 | 28.5 | 2.123 | 0.145 |
| **29758** | 71 | 80 | 75.5 | 75.5 | 0.536 | 0.464 |
| **29759** | 79 | 65 | 72 | 72 | 1.361 | 0.243 |
| **29760** | 101 | 99 | 100 | 100 | 0.020 | 0.888 |
| **29761** | 89 | 94 | 91.5 | 91.5 | 0.137 | 0.712 |
| **29764** | 103 | 105 | 104 | 104 | 0.019 | 0.890 |
| **29765** | 40 | 35 | 37.5 | 37.5 | 0.333 | 0.564 |
| **30531** | 98 | 91 | 94.5 | 94.5 | 0.259 | 0.611 |
| **29782** | 105 | 130 | 117.5 | 117.5 | 2.660 | 0.103 |
| **29785** | 102 | 130 | 116 | 116 | 3.379 | 0.066 |
| **33029** | 98 | 92 | 95 | 95 | 0.189 | 0.663 |
| **33031** | 90 | 101 | 95.5 | 95.5 | 0.634 | 0.426 |
| **29775** | 54 | 67 | 60.5 | 60.5 | 1.397 | 0.237 |
| **29776** | 89 | 92 | 90.5 | 90.5 | 0.050 | 0.824 |
| **29778** | 104 | 100 | 102 | 102 | 0.078 | 0.779 |
| **29779** | 45 | 34 | 39.5 | 39.5 | 1.532 | 0.216 |
| **29828** | 33 | 26 | 29.5 | 29.5 | 0.831 | 0.362 |
| **29829** | 29 | 32 | 30.5 | 30.5 | 0.148 | 0.701 |
| **29837** | 100 | 88 | 94 | 94 | 0.766 | 0.381 |
| **29841** | 93 | 90 | 91.5 | 91.5 | 0.049 | 0.824 |
| **29850** | 24 | 21 | 22.5 | 22.5 | 0.200 | 0.655 |
| **29851** | 88 | 99 | 93.5 | 93.5 | 0.647 | 0.421 |
| **29852** | 103 | 132 | 117.5 | 117.5 | 3.579 | 0.059 |
| **36385** | 45 | 56 | 50.5 | 50.5 | 1.198 | 0.274 |
| **32142** | 108 | 123 | 115.5 | 115.5 | 0.974 | 0.324 |
| **32143** | 99 | 76 | 87.5 | 87.5 | 3.023 | 0.082 |
| **32135** | 46 | 54 | 50 | 50 | 0.640 | 0.424 |
| **32147** | 67 | 77 | 72 | 72 | 0.694 | 0.405 |
| **32149** | 70 | 81 | 75.5 | 75.5 | 0.801 | 0.371 |
| **33252** | 83 | 94 | 88.5 | 88.5 | 0.684 | 0.408 |
| **33256** | 101 | 106 | 103.5 | 103.5 | 0.121 | 0.728 |
| **33243** | 189 | 150 | 169.5 | 169.5 | 4.487 | 0.034 |
| **32529** | 166 | 198 | 182 | 182 | 2.813 | 0.093 |
| **32156** | 154 | 167 | 160.5 | 160.5 | 0.526 | 0.468 |
| **32167** | 144 | 122 | 133 | 133 | 1.820 | 0.177 |
| **32530** | 82 | 94 | 88 | 88 | 0.818 | 0.366 |
| **32533** | 99 | 109 | 104 | 104 | 0.481 | 0.488 |
| **32538** | 82 | 71 | 76.5 | 76.5 | 0.791 | 0.374 |
| **29736** | 44 | 57 | 50.5 | 50.5 | 1.673 | 0.196 |
| **29737** | 33 | 40 | 36.5 | 36.5 | 0.671 | 0.413 |
| **29745** | 98 | 105 | 101.5 | 101.5 | 0.241 | 0.623 |
| **29747** | 102 | 123 | 112.5 | 112.5 | 1.960 | 0.162 |
| **29749** | 103 | 88 | 95.5 | 95.5 | 1.178 | 0.278 |
| **29752** | 19 | 31 | 25 | 25 | 2.880 | 0.090 |
| **29754** | 22 | 34 | 28 | 28 | 2.571 | 0.109 |
| **29794** | 88 | 88 | 61.5 | 61.5 | 0.000 | 1.000 |
| **29795** | 67 | 80 | 73.5 | 73.5 | 1.150 | 0.284 |
| **29797** | 21 | 30 | 25.5 | 25.5 | 1.588 | 0.208 |
| **30459** | 22 | 33 | 27.5 | 27.5 | 2.200 | 0.138 |
| **30460** | 19 | 31 | 25 | 25 | 2.880 | 0.090 |
| **30461** | 26 | 33 | 29.5 | 29.5 | 0.831 | 0.362 |
| **30462** | 44 | 60 | 52 | 52 | 2.462 | 0.117 |
| **30463** | 89 | 71 | 80 | 80 | 2.025 | 0.155 |
